# *Cymbopogon proximus* Chiov’s extract improves insulin sensitivity in rats with dexamethasone-induced insulin resistance and underlying mechanisms

**DOI:** 10.1038/s41598-025-02340-0

**Published:** 2025-06-20

**Authors:** Amany M. Hamed, Noha A. Seif-Eldein, Shymaa A. Thabet, Ahmed S. Osman, Rasha Abdeen Refaei, Ahmed R. H. Ahmed, Azza M. A. Abouelella, Walid H. El-Tantawy, Abeer Temraz, Salwa A. Abu El Wafa

**Affiliations:** 1 Chemistry Department, Faculty of Science, Sohag University, Sohag, Egypt; 2 Pharmacognosy and Medicinal Plants Department, Faculty of Pharmacy for Girls, Al Azhar University, Cairo, Egypt; 3 Central Research Center, Faculty of Medicine, Sohag University, Sohag, Egypt; 4 Department of Biochemistry, Faculty of Veterinary Medicine, Sohag University, Sohag, Egypt; 5 Department of Physiology, Faculty of Medicine, Sohag University, Sohag, Egypt; 6 Department of Pathology, Faculty of Medicine, Sohag University, Sohag, Egypt; 7 Department of Clinical Pharmacology, Faculty of Medicine, Sohag University, Sohag, Egypt; 8 National Organization for Drug Control and Research, Cairo, Egypt

**Keywords:** Insulin resistance, Oxidative stress, *Cymbopogon proximus* extract, Type 2 diabetes, Biochemistry, Plant sciences

## Abstract

The worldwide prevalence of type 2 diabetes mellitus (T2DM) is increasing swiftly. *Cymbopogon proximus *(*C. proximus*) is a wild herbaceous plant utilized as a potent remedy in Egyptian folk medicine, sometimes referred to as “Halfabar.” This study examined the hypoglycemic, hypolipidemic, and antioxidant properties of the methanolic extract from the aerial parts of *C. proximus*, as well as its impact on pancreatic tumour necrosis factor-α (TNF-α) and Glucose Transporter-4 (GLUT4) in skeletal muscles within an experimental model of insulin resistance. Additionally, bioactive metabolites in the extract were analyzed via liquid chromatography-mass spectrometry (LC/MS) technology. Insulin resistance was induced by administering 1 mg/kg of dexamethasone to rats over a period of 14 days. The rats received two doses of the extract: a low dose of 100 mg/kg body weight and a high dose of 200 mg/kg body weight, along with the reference drug; Metformin (M) at a dose of 40 mg/kg body weight, supplied once daily by gastric tube for 14 days. The treatment of dexamethasone led to a significant (*P* < 0.05) elevation in serum fasting glucose, fasting insulin, HOMA-IR, and pancreatic TNF-α, along with a significant (*P* < 0.05) reduction in GLUT4 expression in skeletal muscles. Both extract and reference treatments significantly (*P* < 0.05) mitigated these abnormalities. The highest dose of the extract exhibited a significantly (*P* < 0.05) greater antioxidant impact, a more pronounced reduction in insulin levels and HOMA-IR, as well as an enhanced rise in GLUT4 expression and insulin sensitivity index compared to the lowest dose and the M. Histopathological and immunohistochemical analyses corroborate the biochemical results. The LC–ESI–MS/MS profiling resulted in the characterization and tentative identification of 95 metabolites’ structures. Identified substances purported to possess anti-diabetic effect include apigenin, luteolin, tricin flavone glycosides, cyanidin, malvidin anthocyanin glycosides, and caffeic acid. These findings suggest that *C. proximus* can mitigate insulin resistance. Additional clinical trials are necessary to validate these findings and assess the extract’s effectiveness in human insulin resistance.

**Supplementary Information:**

The online version contains supplementary material available at 10.1038/s41598-025-02340-0.

## Introduction

Diabetes mellitus (DM), a significant non-communicable illness of the twenty-first century, impacts persons across all age groups and results in morbidity and mortality^1^. Insulin resistance, often referred to as a prediabetic state, underpins the pathophysiology of Type 2 Diabetes Mellitus (T2DM)^2–4^. This occurs when the metabolic function of insulin to promote glucose absorption is impaired and/or when insulin fails to inhibit hepatic gluconeogenesis and the release of glucose into the bloodstream^5,6^, ultimately leading to malfunction mediated by oxidative stress^7,8^. The prevalence of T2DM is rising globally, with projections indicating that 439 million individuals would be affected by 2030^9^.

Significant advancements in the treatment of T2DM with commercial pharmaceuticals have occurred; nonetheless, these medications exhibit several adverse effects. Furthermore, they are frequently inaccessible to numerous local residents due to their economic circumstances and distribution^10^. Consequently, the researchers focus on investigating novel medication candidates derived from natural compounds that exhibit minimal and mild side effects^11^. Furthermore, WHO advocated for the evaluation of traditional herbal treatments employed in diabetes management due to their efficacy and minimal or negligible toxicity relative to commercial oral anti-diabetic pharmaceuticals^12^.

*Cymbopogon*, a genus of plants with numerous species recognized for their substantial essential oil content, is extensively dispersed across the tropical and subtropical areas of Asia, Africa, and America^13^. *Cymbopogon proximus (Poaceae),* referred to as “Halfabar,” is a wild herbaceous species. The aqueous extract of its desiccated leaves and stems is administered orally as a renal antispasmodic and diuretic in Egyptian traditional medicine^14–17^.

Previous investigations have documented the hypoglycemic effects of various *Cymbopogon* species, including *C. jwarancusa*^18^. Prior scientific investigations have not assessed the antidiabetic properties of *C. proximus*. This study aimed to examine the hypoglycemic effect of methanolic extract from *C. proximus* aerial parts, as well as its impact on pancreatic TNF-α, GLUT4 in skeletal muscles, and proliferating cell nuclear antigen (PCNA) in a dexamethasone-induced model of insulin resistance, while also profiling its bioactive metabolites through UPLC-ESI-MS/MS analysis.

## Results

### UPLC-ESI–MS/MS analysis of bioactive compounds in C. proximus extract

**Fig. 1:**
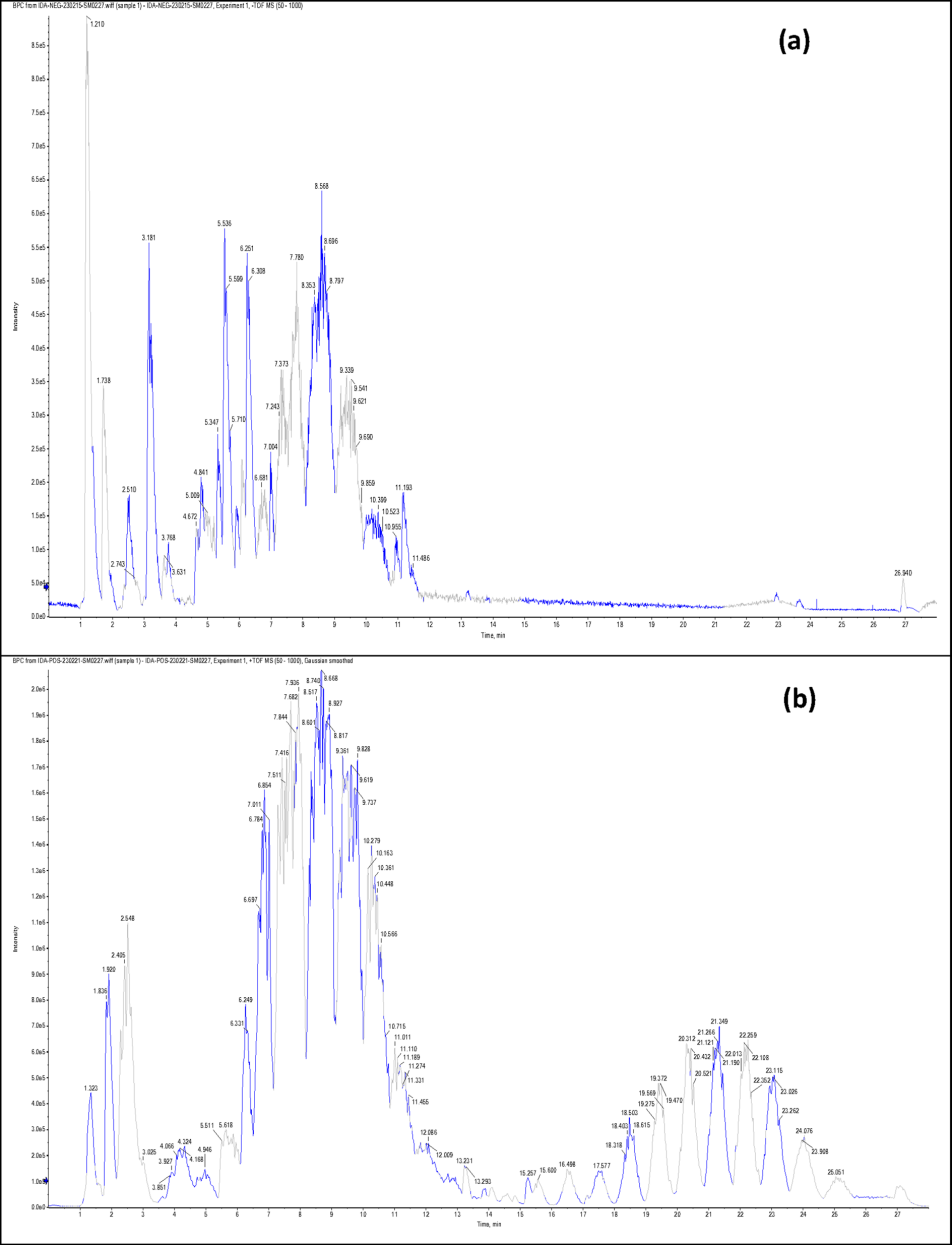
(**a**) Negative-mode– Base Peak Chromatogram (BPC), (**b**) Positive- mode– Base Peak Chromatogram (BPC) of *C. proximus* extract using UPLC-ESI–MS/MS.

**Fig. 2:**
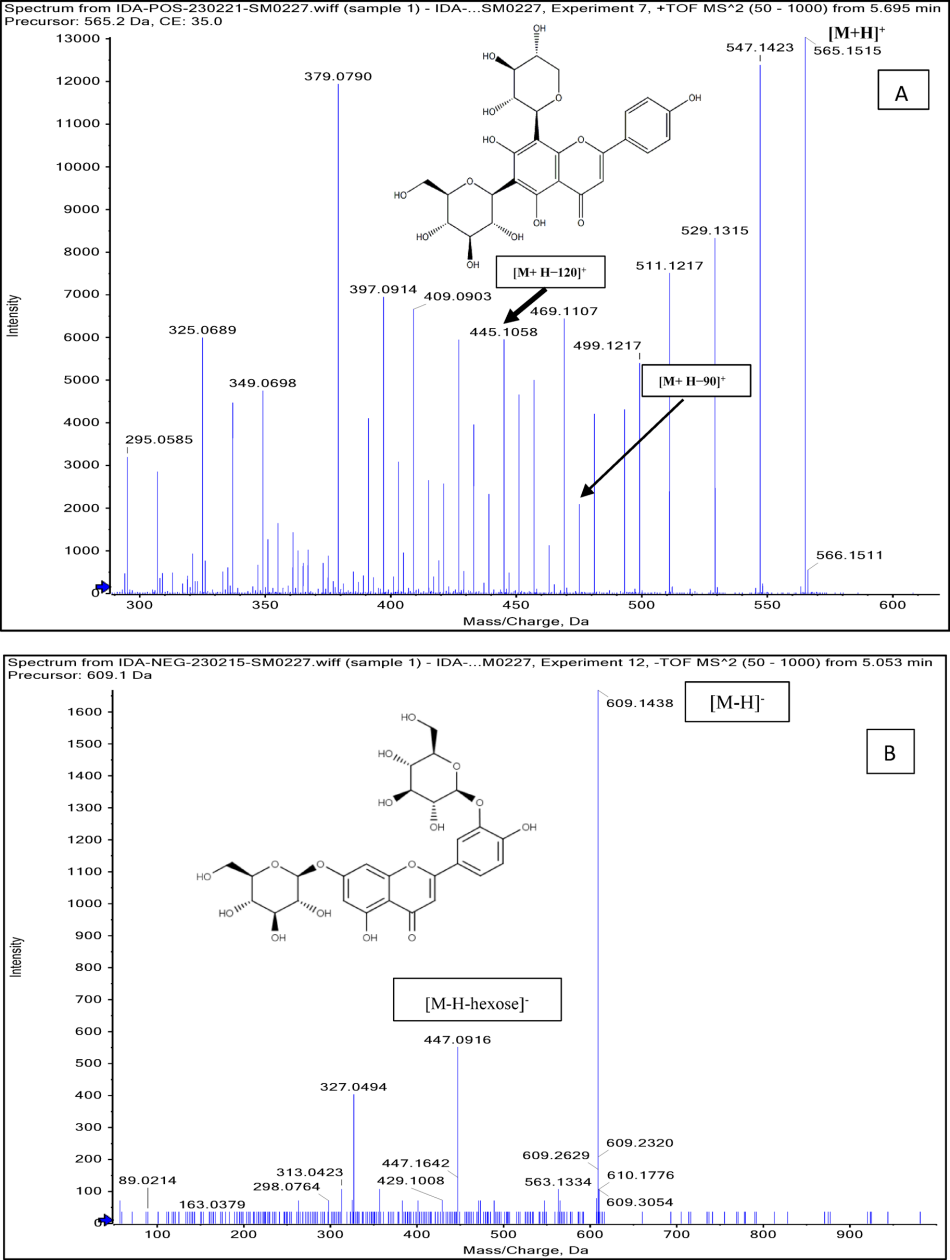
Mass spectra of (**A**) 8-C-xylosyl-6-C-glucosyl apigenin, (**B**) Luteolin 3’, 7-di-*O*-glucoside identified in *C. proximus* extract using UPLC-ESI–MS/MS.

Negative and positive ionization techniques were employed to detect the metabolites in the aerial portions of *C. proximus*. This study is the inaugural publication on the metabolic profiling of *C*. *proximus* extract utilizing the UPLC-MS/MS technology. A total of 95 metabolites from diverse classes were provisionally discovered and classified into flavonoids, phenolic and carboxylic acids, coumarins, stilbenes, and alkaloids in the extract of *C. proximus*. Flavonoids and phenolic acids constituted the predominant category among all detected chemicals. The identification was based on matching the acquired mass data with library databases and previously published literature, accounting for an error margin of (± 10). The assigned metabolites were organized in the order of their elution times (in negative mode) in the analyzed extract among each class and were presented in Table 1. The total ion chromatograms of *C. proximus* extract in positive and negative ion modes were presented in Fig. 1. The predominant group of secondary metabolites was flavonoids, with 55 unique peaks. The delayed elution of flavonoids, in contrast to phenolic acids, corresponds with their relatively polar properties^19^. Several subclasses of flavonoids were identified in UPLC-MS/MS as flavones, flavonols, flavanones, isoflavones, flavanol, flavanonol, and anthocyanins, with flavones amounting to be the major subclass. Apigenin, luteolin, and tricin were the predominant flavone derivatives discovered in the extract of *C. proximus* based on peak area analysis. This aligns with previous reports on Cymbopogon species^20^. Similarly, representatives of Poaceae demonstrated an identical tendency. Four categories of flavone glycosides were found in the *C. proximus* extract: *O*-glycosyl flavonoids, *C*-glycosyl flavonoids, *C,C*-diglycosyl flavonoids, and *O,C*-diglycosyl flavonoids. These were validated by examining their unique fragmentation patterns. The mass spectra of *O*-glycosyl flavonoids typically exhibit ions at m/z [M + H − glycone] + in positive ion mode and [M − H − glycone] − in negative ion mode, possibly resulting from cleavage at the glycosidic O-linkage (Fig. 2). Conversely, in C-glycosyl flavonoids, the majority of fragment ions, including those at m/z [M ± H − 60], [M ± H − 90], and [M ± H − 120] in both ion modes, were associated with cross-ring cleavage in their sugar moieties (Fig. 2). *O*- and *C*-diglycosyl flavonoids exhibited minimal fragment ion presence of aglycone, while simultaneously demonstrating a large abundance of fragmentation for [M − H − glycone] − and [M − H − glycone − H_2_O] − ^21^. O-hexosyl, O-deoxyhexosyl, and O-pentosyl derivatives were identified by the presence of a prominent ion [M + H −162] + , [M + H − 146] + , or [M + H − 132] + ^22^.

**Table 1:**
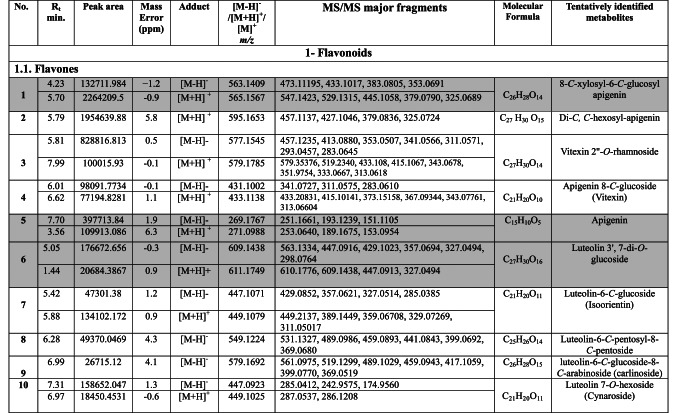

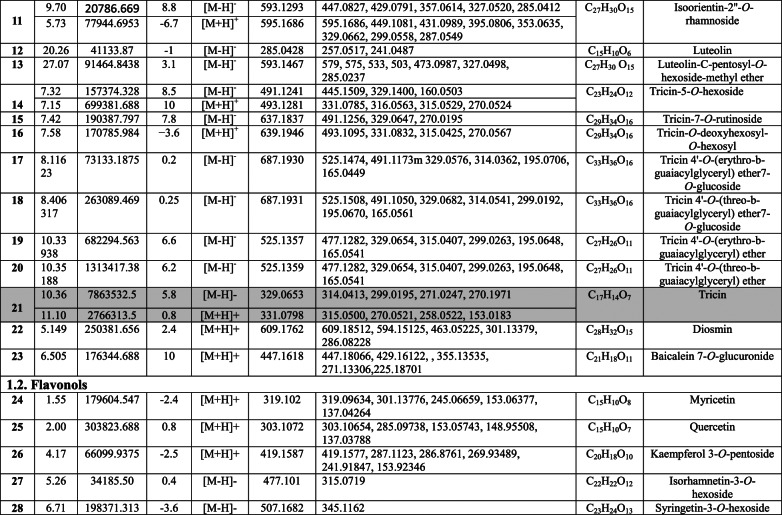

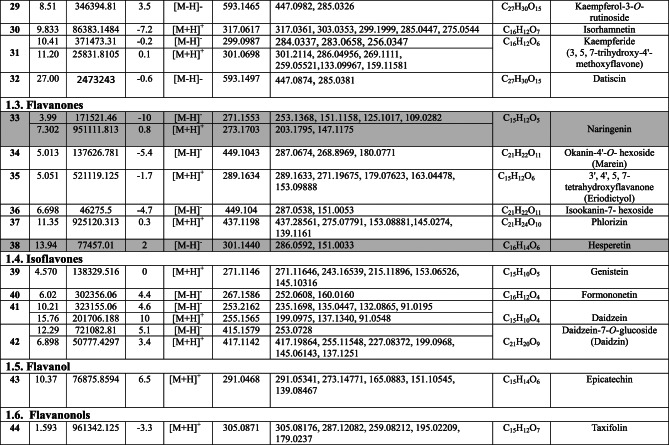

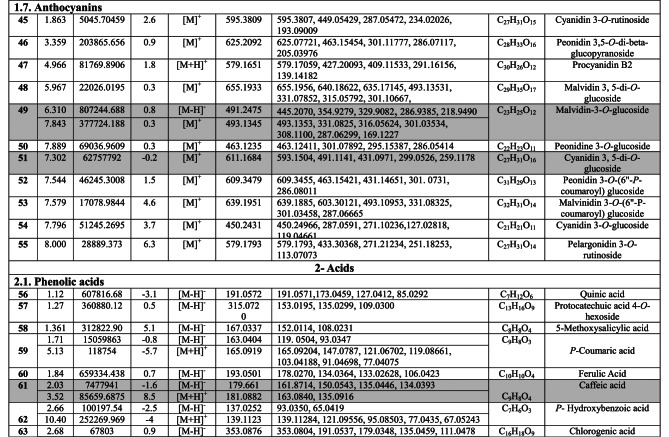

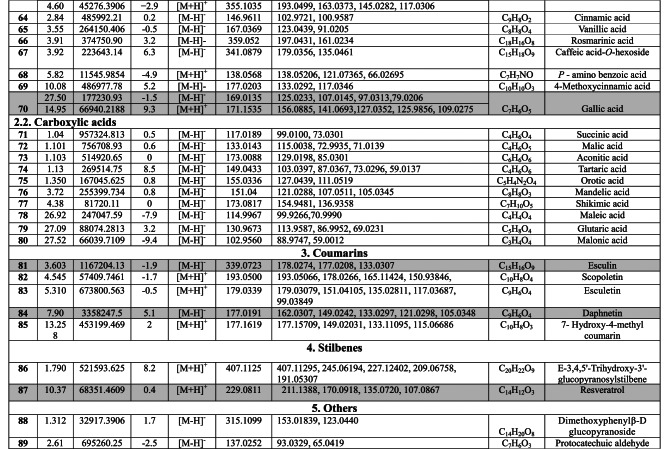

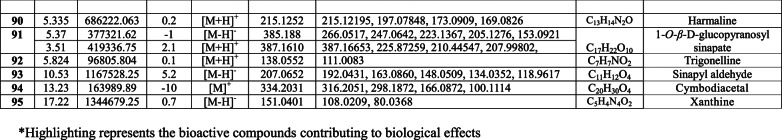
Secondary metabolites tentatively identified in *C. proximus* extract using UPLC-ESI- MS/MS in negative and positive ionization modes.

*Highlighting represents the bioactive compounds contributing to biological effects.

The chromatographic analysis demonstrated a substantial quantity of anthocyanin peaks. The extract of *C. proximus* included four prevalent types of anthocyanins: cyanidin, malvidin, peonidin, and pelargonidin glycosides (Table 1).

Phenolic acids constituted the second most prevalent category of secondary metabolites identified in *C. proximus*. Two subcategories of phenolic acids, mostly categorized as hydroxybenzoic and hydroxycinnamic acids, were recognized. The predominant hydroxycinnamic acid in the extract was *p*-coumaric acid, succeeded by chlorogenic acid and then caffeic acid, but gallic acid was the most abundant benzoic acid derivative (Table 1).

Five coumarins were identified in the extract, with daphnetin being the most prevalent coumarin based on peak area analysis. Resveratrol was among the detected stilbenes in the extract of *C. proximus*. The comprehensive analysis of mass data and fragmentation patterns of the principal detected compounds is detailed in Supplementary 1, whereas the images depicting MS/MS fragmentation of the majority of identified compounds in both ionization modes are provided in Supplementary 2, 3 and 4.

### Biochemical study

#### Effect of treatment on body weight

**Fig. 3:**
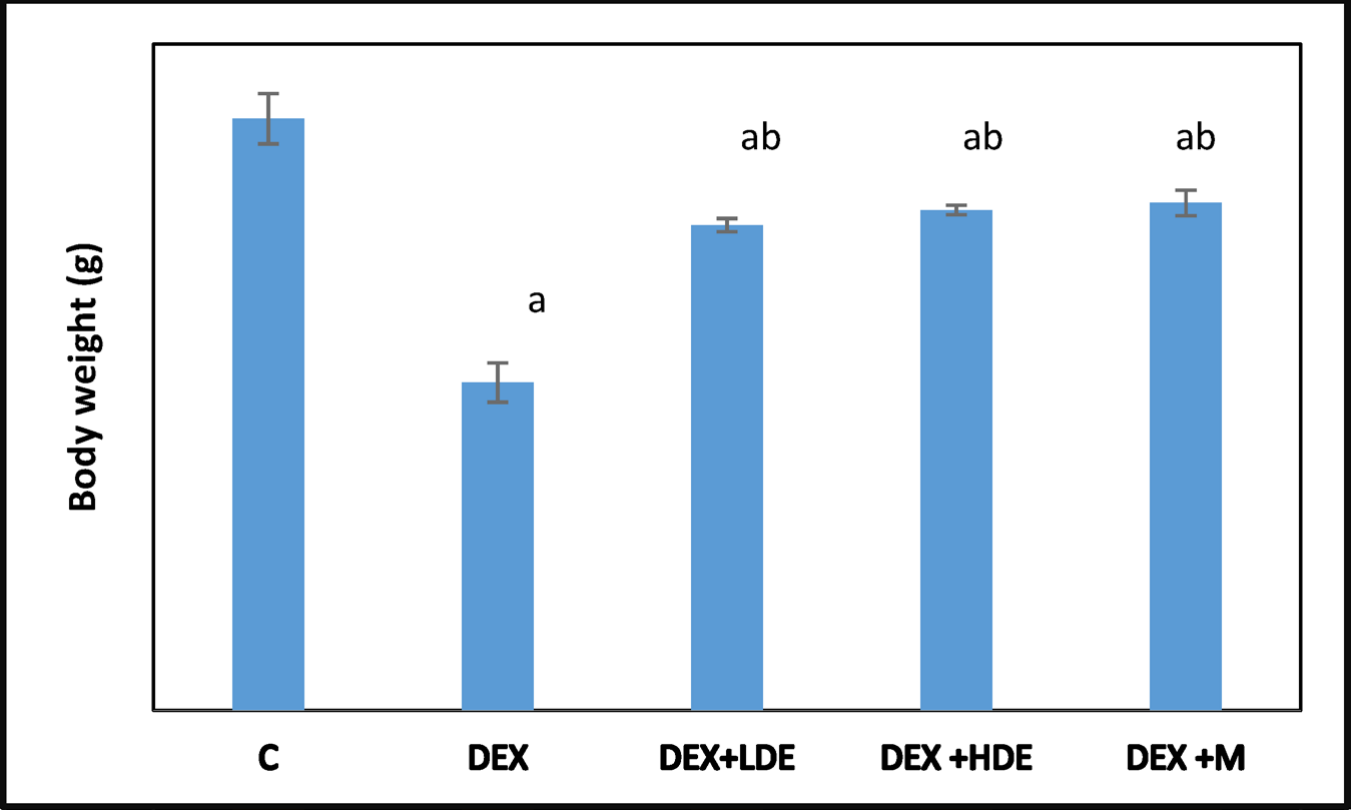
Body weight in different experimental groups. Results are expressed as mean ± S.E.M. ^a^*P* < 0.05: Significantly different from control group. ^b^*P* < 0.05: Significantly different from DEX treated group. C: control group, DEX: dexamethasone-treated group, DEX + LDE: dexamethasone + low dose extract, DEX + HDE: dexamethasone + high dose extract, DEX + M: dexamethasone + metformin.

The DEX-treated group exhibited a significant (*P* < 0.05) reduction in body weight relative to their respective controls. Conversely, administration of both doses of the extract and M (the reference medication utilized in this investigation) resulted in a significant (*P < *0.05) increase in body weight compared to the DEX-treated rats (Fig. 3).

#### Effect of treatment on serum fasting glucose, insulin, and HOMA-IR

**Fig. 4:**
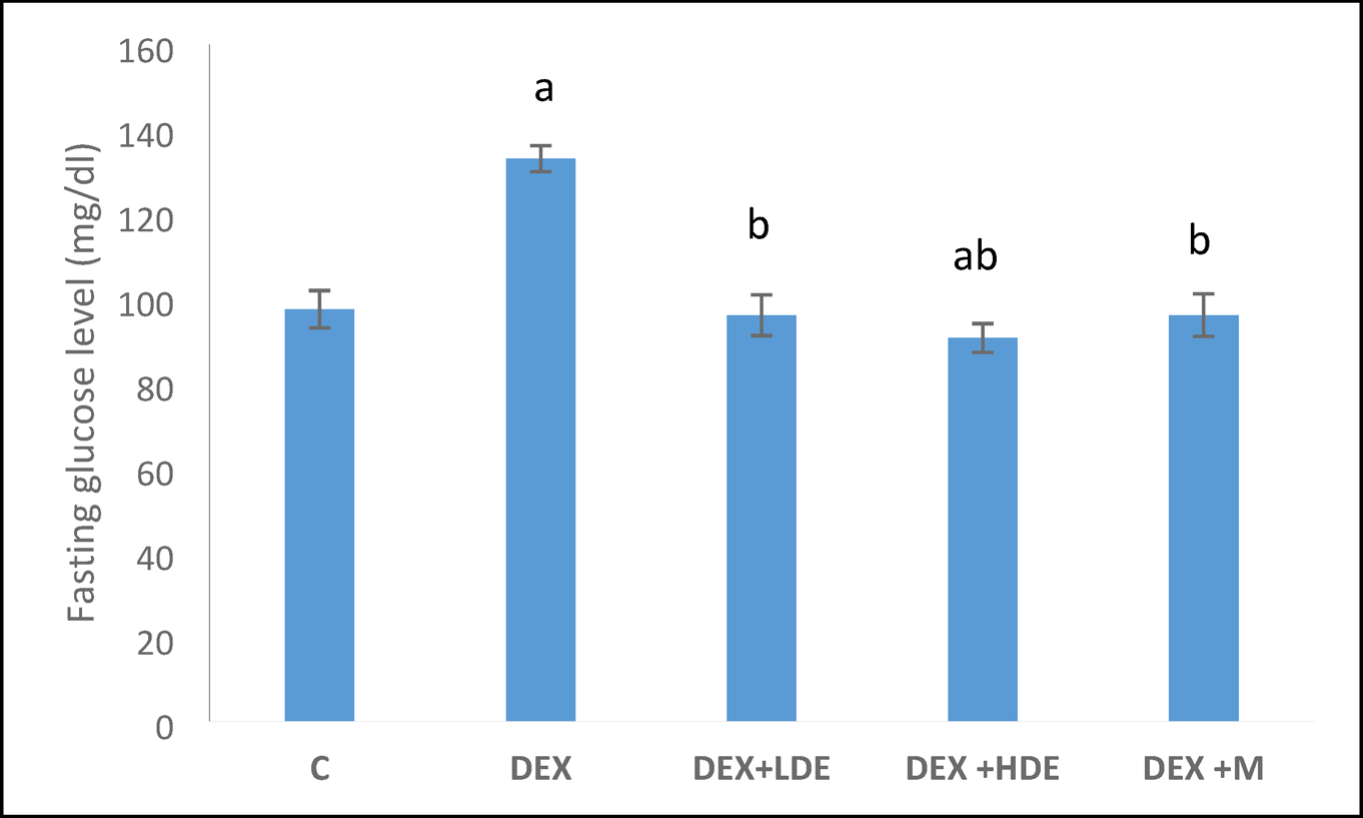
Serum fasting glucose level in different experimental groups. Results are expressed as mean ± S.E.M. ^a^*P* < 0.05: Significantly different from control group. ^b^*P* < 0.05: Significantly different from DEX treated group. C: control group, DEX: dexamethasone-treated group, DEX + LDE: dexamethasone + low dose extract, DEX + HDE: dexamethasone + high dose extract, DEX + M: dexamethasone + metformin.

**Fig. 5:**
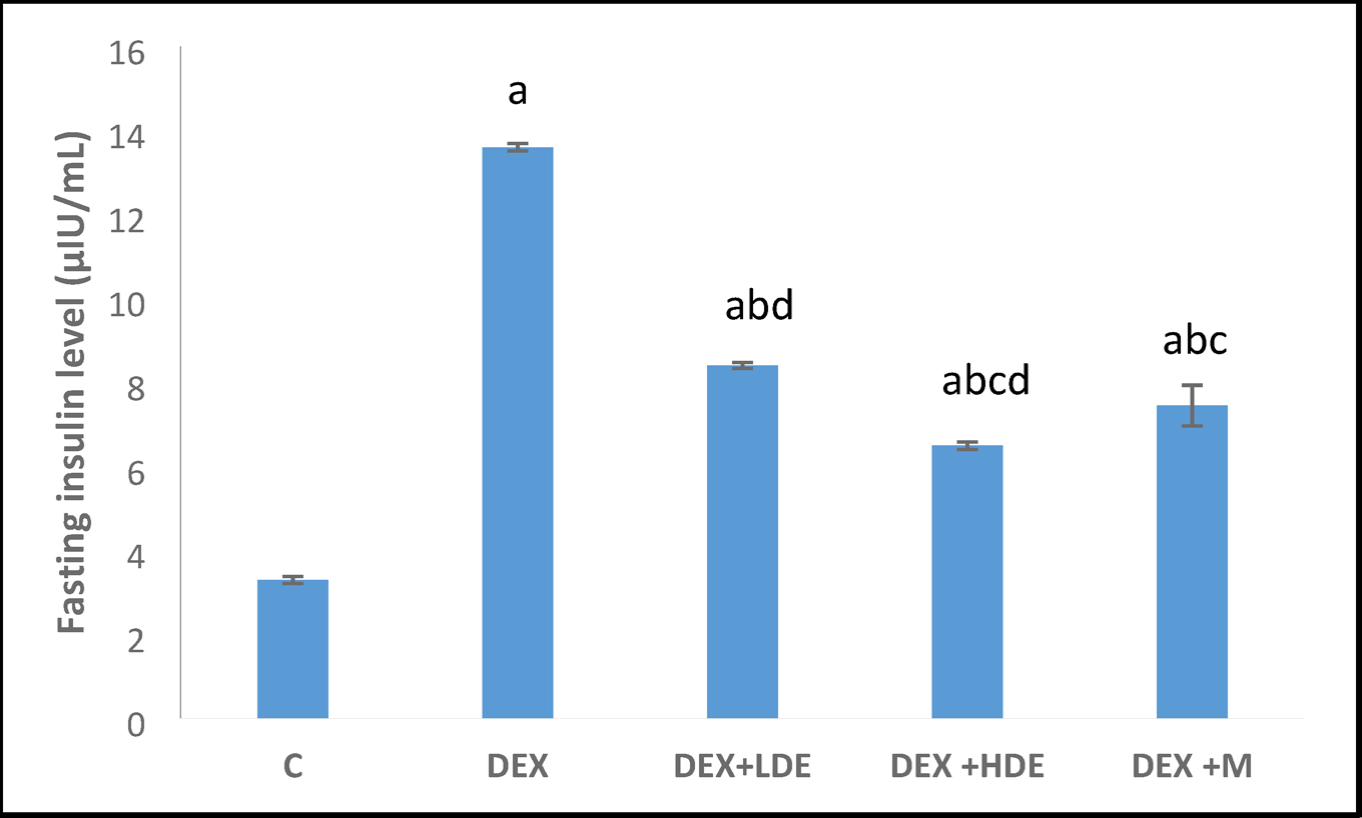
Serum fasting insulin level in different experimental groups. Results are expressed as mean ± S.E.M. ^a^*P* < 0.05: Significantly different from control group. ^b^*P* < 0.05: Significantly different from DEX treated group. ^c^*P* < 0.05: Significantly different from DEX + LDE-treated group. ^d^*P* < 0.05: Significantly different from DEX + M-treated group. C: control group, DEX: dexamethasone-treated group, DEX + LDE: dexamethasone + low dose extract, DEX + HDE: dexamethasone + high dose extract, DEX + M: dexamethasone + metformin.

**Fig. 6:**
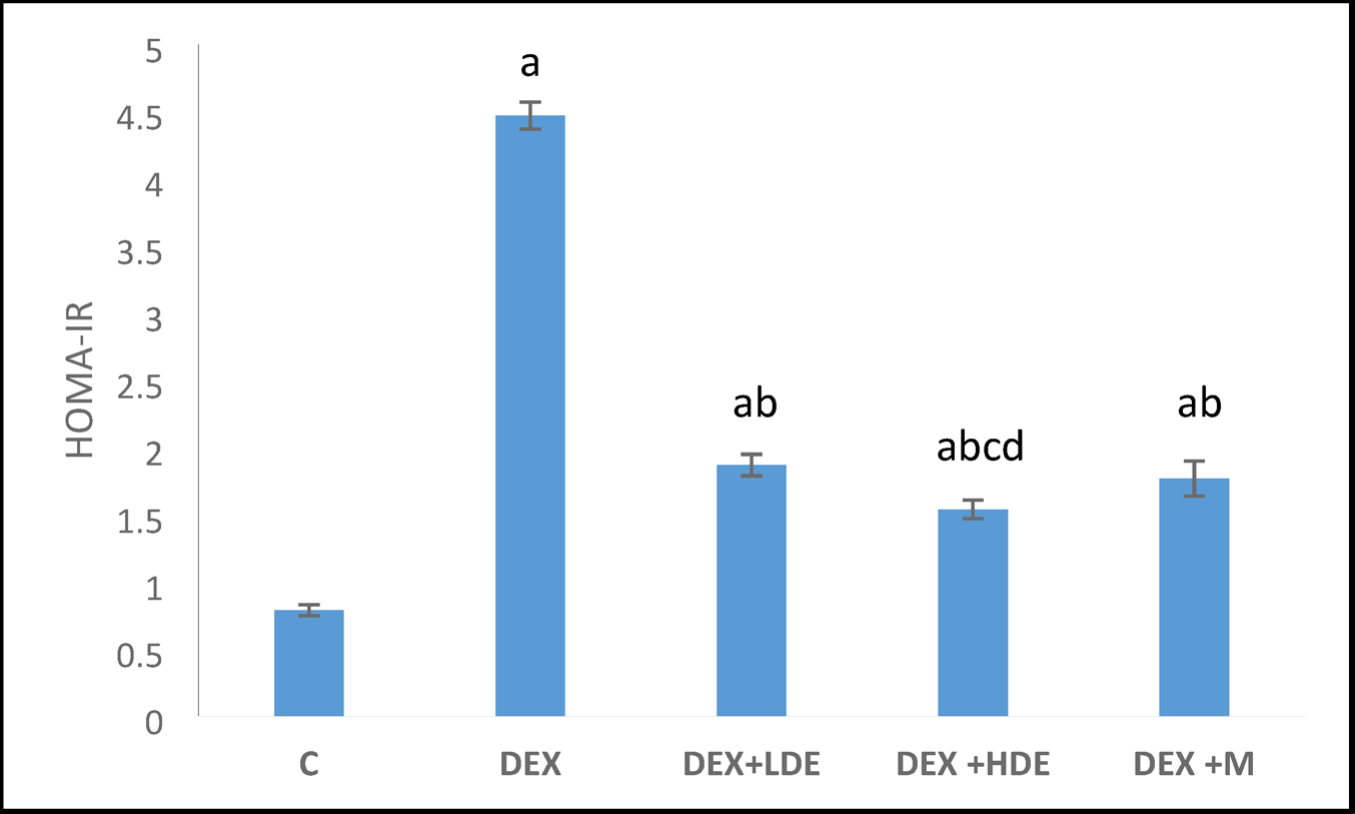
HOMA-IR in different experimental groups. Results are expressed as mean ± S.E.M. ^a^*P* < 0.05: Significantly different from control group. ^b^*P* < 0.05: Significantly different from DEX treated group. ^c^*P* < 0.05: Significantly different from DEX + LDE-treated group. ^d^*P* < 0.05: Significantly different from DEX + M-treated group. C: control group, DEX: dexamethasone-treated group, DEX + LDE: dexamethasone + low dose extract, DEX + HDE: dexamethasone + high dose extract, DEX + M: dexamethasone + metformin.

As illustrated in Figs. 4, 5, and 6, fasting blood glucose, insulin, and HOMA-IR levels in DEX-treated rats were significantly elevated (*P* < 0.05) compared to their corresponding control levels. Nonetheless, the administration of both doses of the extract and the M to DEX-treated rats significantly (*P* < 0.05) diminished these parameters in comparison to the DEX-treated group. The DEX + HDE therapy significantly reduced fasting insulin and HOMA-IR compared to the DEX + LDE and DEX + M treatments (*P* < 0.05).

#### Effect of treatment on insulin sensitivity index

**Fig. 7:**
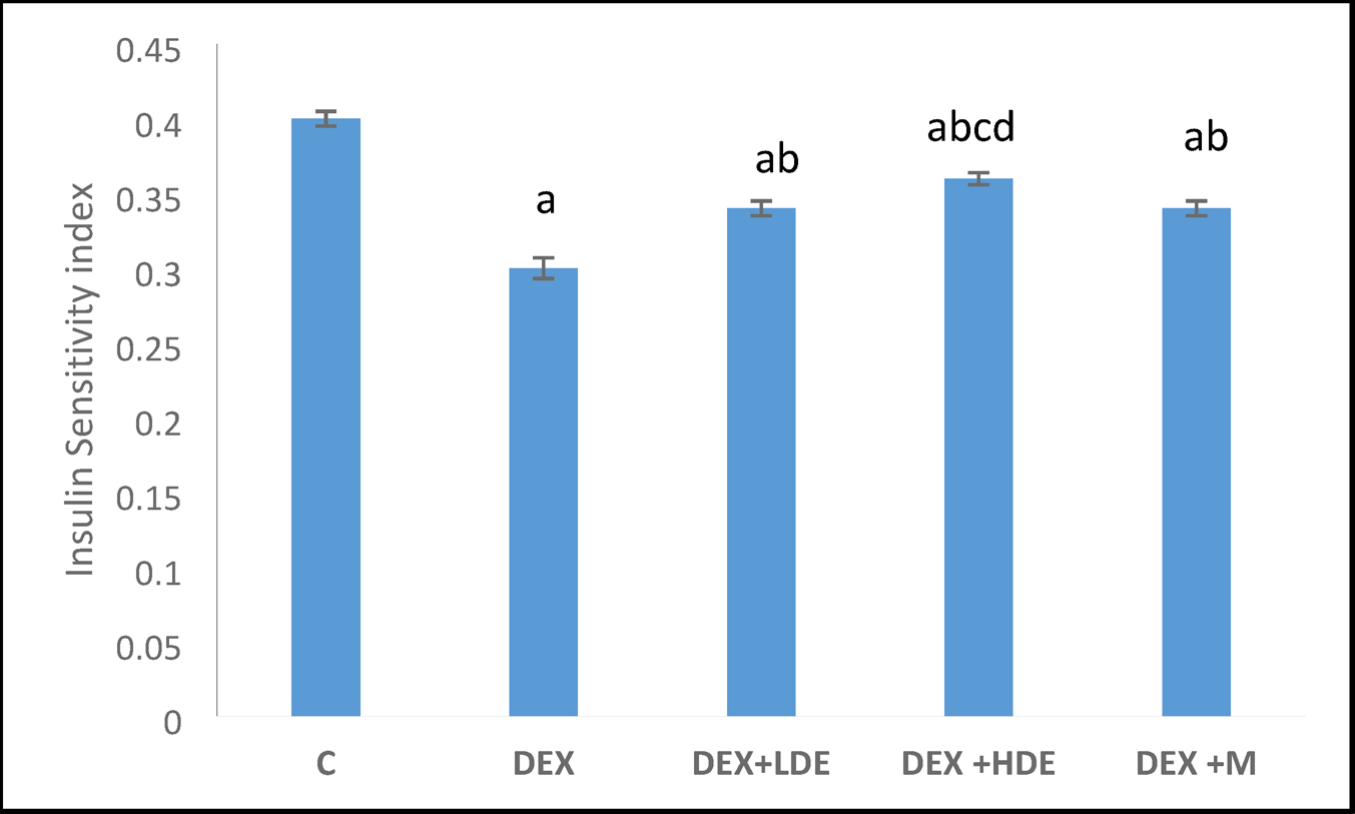
Insulin sensitivity index in different experimental groups. Results are expressed as mean ± S.E.M. ^a^*P* < 0.05: Significantly different from control group. ^b^*P* < 0.05: Significantly different from DEX treated group. ^c^*P* < 0.05: Significantly different from DEX + LDE-treated group. ^d^*P* < 0.05: Significantly different from DEX + M-treated group. C: control group, DEX: dexamethasone-treated group, DEX + LDE: dexamethasone + low dose extract, DEX + HDE: dexamethasone + high dose extract, DEX + M: dexamethasone + metformin.

The administration of DEX significantly (*P* < 0.05) reduced the insulin sensitivity index compared to the matched control group. Treatment with the extract and M exhibited a significant (*P* < 0.05) increase in the insulin sensitivity index relative to the DEX-administered rats. According to our findings in Fig. 7, the HDE treatment exhibited a substantial increase (*P* < 0.05) compared to the LDE and M treatments.

#### Effect of treatment on GLUT4 expression

**Fig. 8:**
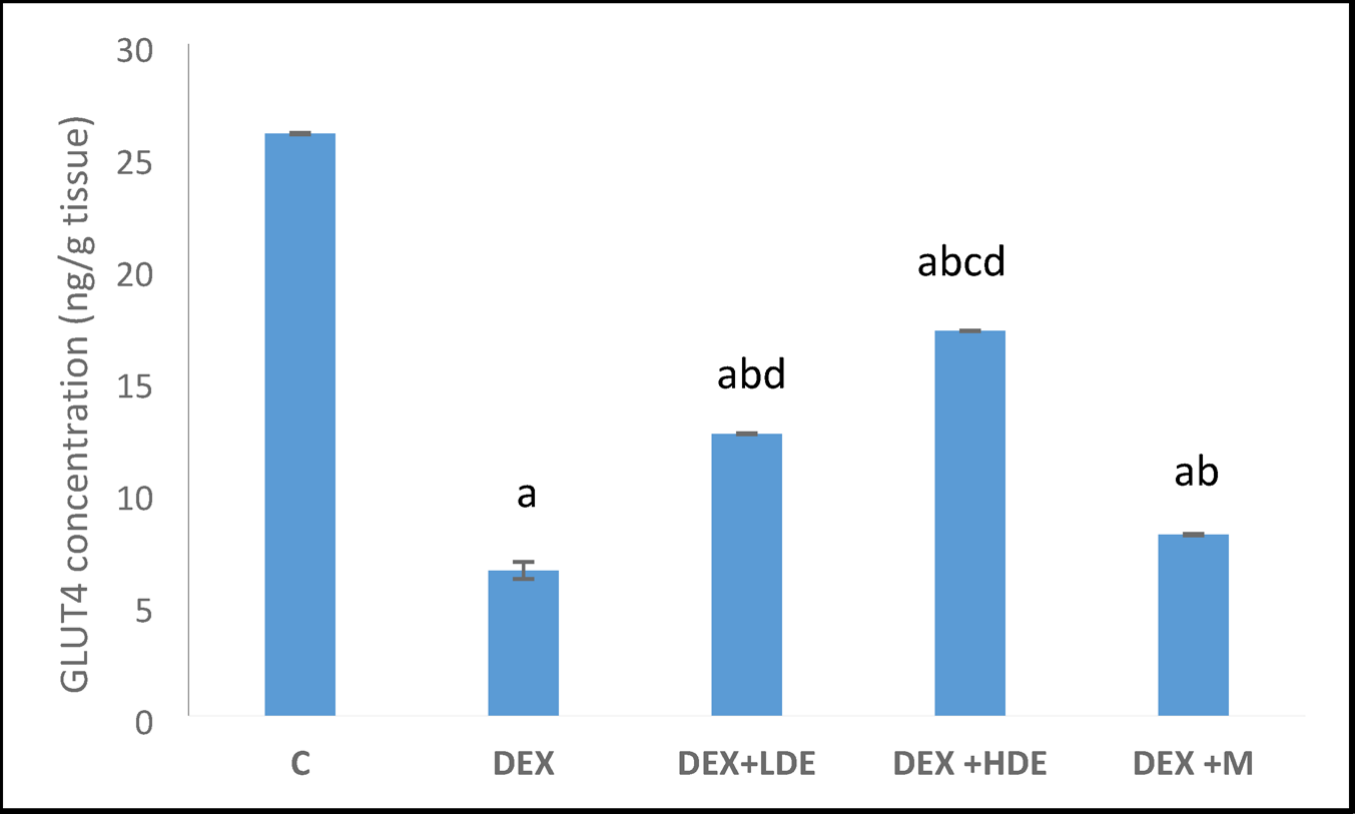
GLUT4 concentration in different experimental groups. Results are expressed as mean ± S.E.M. ^a^*P* < 0.05: Significantly different from control group. ^b^*P* < 0.05: Significantly different from DEX treated group. ^c^*P* < 0.05: Significantly different from DEX + LDE-treated group. ^d^*P* < 0.05: Significantly different from DEX + M-treated group. C: control group, DEX: dexamethasone-treated group, DEX + LDE: dexamethasone + low dose extract, DEX + HDE: dexamethasone + high dose extract, DEX + M: dexamethasone + metformin.

DEX injection demonstrated a substantial (*P* < 0.05) reduction in GLUT4 expression relative to the equivalent controls. Conversely, the administration of both dosages of the extract and M resulted in a significant (*P* < 0.05) increase in GLUT4 concentration compared to DEX-treated rats. Furthermore, the rats administered HDE exhibited a statistically significant increase (*P* < 0.05) in comparison to those given with LDE and M (Fig. 8).

#### Effect of treatment on SOD activity, GSH, and MDA levels in the liver and pancreatic tissues

Treatment of rats with DEX demonstrated a significant reduction in SOD activity and GSH levels, alongside a notable increase in MDA levels compared to their respective controls (*P* < 0.05)*.* Both doses of the extract and the M administration greatly improved these modifications. Furthermore, the DEX + HDE markedly enhanced SOD activity and GSH levels while reducing MDA levels compared to the DEX + LDE and DEX + M treatments (*P* < 0.05) (Table 2).

**Table 2:** MDA, GSH level, and SOD activity of hepatic and pancreatic homogenates in C, DEX, DEX + LDE, DEX + HDE and DEX + M treated groups.

	Hepatic MDA (nmol/g tissue)	Hepatic GSH (Pg/g tissue)	Hepatic SOD (U/g tissue)	Pancreatic MDA (nmol/g tissue)	Pancreatic GSH (Pg/g tissue)	Pancreatic SOD (U/g tissue)
C	7.2 ± 0.06	45 ± 0.07	38.2 ± 0.04	5.7 ± 0.01	35 ± 0.07	29.7 ± 0.02
DEX	34.2 ± 0.07^a^	14.7 ± 0.08^a^	12.2 ± 0.05^a^	23.8 ± 0.02^a^	11.4 ± 0.02^a^	9.4 ± 0.01^a^
DEX + LDE	20.1 ± 0.05^abd^	25.7 ± 0.14^abd^	21.3 ± 0.02^abd^	15.4 ± 0.02^abd^	20.4 ± 0.03^abd^	16.9 ± 0.02^abd^
DEX + HDE	16.4 ± 0.12^abcd^	31.4 ± 0.09^abcd^	26.4 ± 0.02^abcd^	10.2 ± 0.01^abcd^	24.8 ± 0.02^abcd^	20.7 ± 0.02^abcd^
DEX + M	29.7 ± 0.1^ab^	17 ± 1.17^ab^	14.5 ± 0.03^ab^	20.4 ± 0.02^ab^	13.9 ± 0.07^ab^	11.5 ± 0.01^ab^

Results are expressed as mean ± S.E.M

C, control group; DEX, dexamethasone-treated group; DEX + LDE, dexamethasone + low dose extract; DEX + HDE, dexamethasone + high dose extract; DEX + M, dexamethasone + metformin (Reference drug).

^a^*P* < 0.05:Significantly different from control group.

^b^*P* < 0.05:Significantly different from DEX treated group.

^c^*P* < 0.05:Significantly different from DEX + LDE-treated group.

^d^*P* < 0.05:Significantly different from DEX + M-treated group.

#### Effect of treatment on lipid profile

The administration of DEX resulted in a significant (*P* < 0.05) increase in total serum cholesterol, triglycerides, and LDL-cholesterol, along with a notable decrease in HDL-cholesterol when compared to the corresponding controls. Both doses of the extract and the M significantly (*P* < 0.05) improved these anomalies. Regarding blood cholesterol, triglycerides, and LDL-cholesterol, the M elicited a significant decrease in these parameters (*P* < 0.05) in comparison to those administered both dosages of the extract (Table 3).

**Table 3:** Lipid profile in C, DEX, DEX + LDE, DEX + HDE and DEX + M treated groups.

	Cholesterol (mg/dl)	Triglycerides (mg/dl)	HDL-Cholesterol (mg/dl)	LDL-Cholesterol (mg/dl)
C	84 ± 1.32	46 ± 1.26	26.3 ± 0.76	48.5 ± 1.11
DEX	196.2 ± 0.96^a^	163 ± 3.39^a^	5.4 ± 0.5^a^	158.2 ± 1.265^a^
DEX + LDE	119 ± 0.38^abd^	80.8 ± 0.7^abd^	21.3 ± 0.25^abd^	82.1 ± 0.285^abd^
DEX + HDE	121 ± 0.57^abd^	82.6 ± 0.8^abd^	22.9 ± 0.28^abc^	81.4 ± 0.515^abd^
DEX + M	111.3 ± 1^ab^	71.3 ± 0.89^ab^	23.4 ± 0.67^ab^	73.7 ± 1.65^ab^

Results are expressed as mean ± S.E.M

C, control group; DEX, dexamethasone-treated group; DEX + LDE, dexamethasone + low dose extract; DEX + HDE, dexamethasone + high dose extract; DEX + M, dexamethasone + metformin (Reference drug).

^a^*P* < 0.05:Significantly different from control group.

^b^*P* < 0.05:Significantly different from DEX treated group.

^c^*P* < 0.05:Significantly different from DEX + LDE-treated group.

^d^*P* < 0.05:Significantly different from DEX + M-treated group.

#### Effect of treatment on serum albumin level, ALT, and AST activities

DEX treatment led to a marked reduction in serum albumin and a notable increase in serum ALT and AST activity relative to their respective controls (*P* < 0.05)*.* Treatment with the extract and the M significantly (*P* < 0.05) mitigated these alterations. Concerning ALT and AST, the DEX + HDE significantly (*P* < 0.05) reduced their activities compared to the DEX + LDE and DEX + M treatments (Table 4).

**Table 4:** Serum albumin level, ALT, and AST activities in C, DEX, DEX + LDE, DEX + HDE and DEX + M treated groups.

	Albumin g/dl	ALT U/L	AST U/L
C	3.6 ± 0.1	39.3 ± 0.34	55.2 ± 0.2
DEX	2.1 ± 0.08^a^	182.1 ± 0.19^a^	150.4 ± 0.29^a^
DEX + LDE	3.7 ± 0.2^b^	58.3 ± 0.64^abd^	70.6 ± 0.47^abd^
DEX + HDE	3.8 ± 0.07^abd^	46.4 ± 0.29^abcd^	58.5 ± 0.19^abcd^
DEX + M	3.7 ± 0.08^b^	100.5 ± 0.56^ab^	88.4 ± 0.35^ab^

Results are expressed as mean ± S.E.M

C, control group; DEX, dexamethasone-treated group; DEX + LDE, dexamethasone + low dose extract; DEX + HDE, dexamethasone + high dose extract; DEX + M, dexamethasone + metformin (Reference drug).

^a^*P* < 0.05: Significantly different from control group.

^b^*P* < 0.05: Significantly different from DEX treated group.

^c^*P* < 0.05: Significantly different from DEX + LDE-treated group.

^d^*P* < 0.05: Significantly different from DEX + M-treated group.

#### Histopathological evaluation of liver tissue

**Fig. 9:**
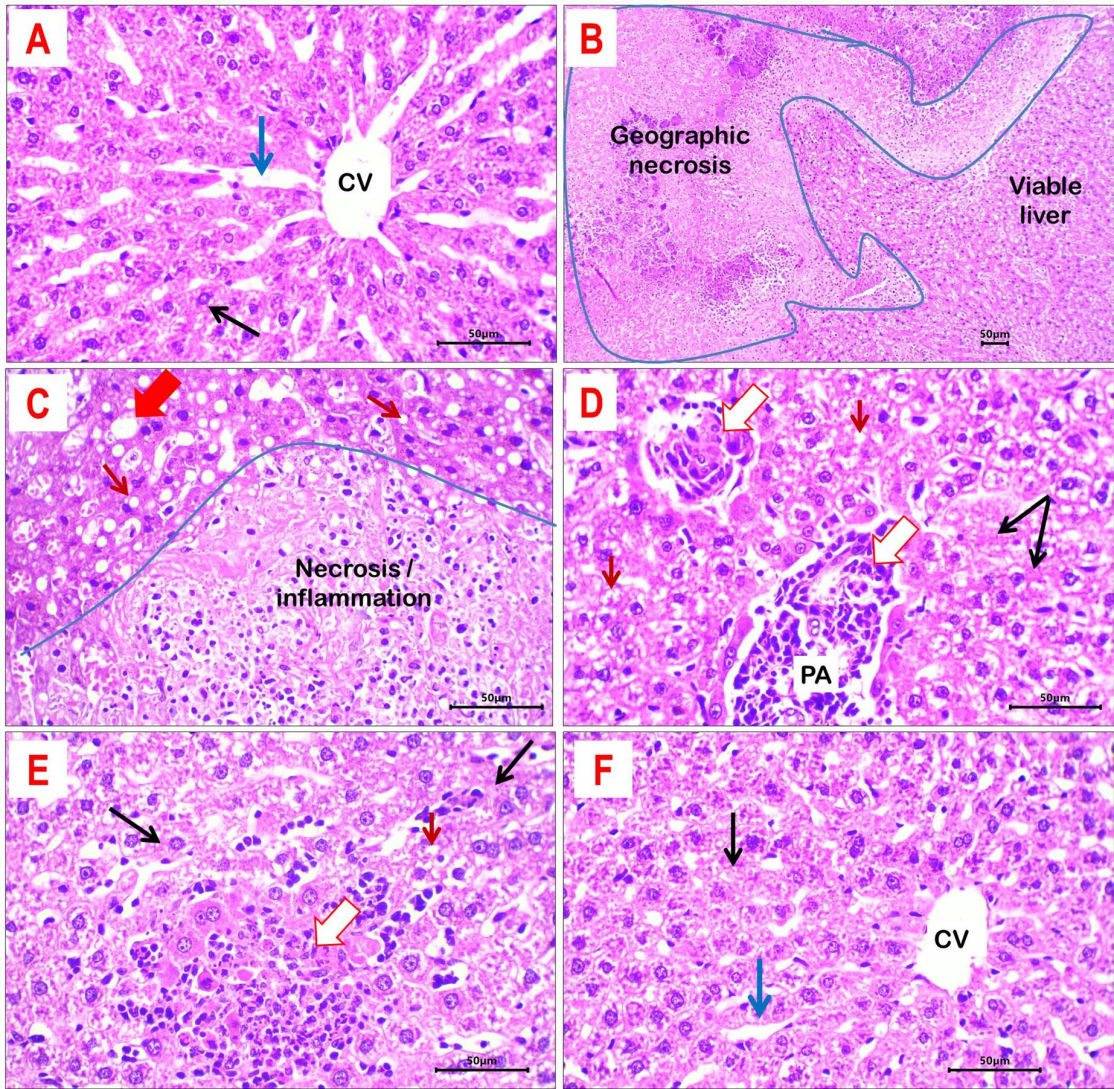
Histological sections of liver tissue from different study groups: (**A**) control group showed normal hepatic morphology with identified central vein (CV) and uniform hepatic cords (black arrow), separated by radiating sinusoids (blue arrow). (**B**, **C**) liver tissue of DEX-treated rats showed large areas of geographic necrosis with dense inflammation. Hepatocytes showed frequent micro-vesicular (thin red arrows) and macro-vesicular (thick red arrow) steatosis. (**D**) DEX + M-treated rats; showed mild degenerative changes (granular cytoplasm and cloudy swelling; black arrows), residual inflammation (white arrows) at portal areas (PA) and frequent micro-vesicular steatosis (red arrows). (**E**) DEX + LDE-treated rats; showed residual lobular necro-inflammatory debris (white arrow). Hepatocytes showed focal granular cytoplasm and cloudy swelling (black arrows) and micro-vesicular steatosis (red arrow). (**F**) DEX + HDE; induced remarkable improvement of hepatic morphology compared to DEX-treated rats. Hepatocytes have almost retained architecture with preserved hepatocyte cording (black arrow) and intervening sinusoids (blue arrow). H&E-stained sections; magnification is × 100 for B and × 400 for others.

In the control group, the liver exhibited intact normal hepatic lobulation, with discernible central venules and portal tracts. Hepatocytes are organized in two-cell-thick plates, with intermediate sinusoids intact. Their morphology is polyhedral, featuring uniformly positioned nuclei on the ventral side (Fig. 9A). No indications of inflammation, hepatocellular degeneration, or necrosis were observed in the control group.

The administration of DEX caused significant hepatic tissue damage (Fig. 9B,C). Numerous loci of geographic necrosis are encircled by areas of inflammation. Central venules and hepatic sinusoids exhibited frequent congestion. Hepatocytes exhibited extensive degenerative alterations, including hazy swelling, granular cytoplasm, and micro- and macro-vesicular steatosis. Furthermore, there is irregular lobular and portal inflammation characterized by an abundance of neutrophils and lymphocytes.

Treatment with M demonstrated an enhancement in hepatic morphological alterations compared to DEX-treated rats, with little necrosis. Residual mild venous and sinusoidal congestion, patchy microvesicular steatosis, and hazy swelling of hepatocytes were present. Portal and lobular inflammation abundant in lymphocytes was also observed (Fig. 9D).

Likewise, administration of LDE resulted in a similar enhancement in liver morphology when compared to M-treated rats (Fig. 9E).

In the DEX + HDE-treated group, liver tissue exhibited preserved normal architecture, with normal hepatocyte arrangement and no necrosis (Fig. 9F).

The main histological alterations of liver tissue across several groups are encapsulated in Table 5.

**Table 5:** Main histopathological findings of liver tissues in different study groups.

Parameter	Study groups				
	C	DEX	DEX + M	DEX + LDE	DEX + HDE
Necrosis	−	+ + + +	+	+	−
Central veins and sinusoids congestion	−	+ + + +	+ +	+ +	+
Steatosis	−	+ + + +	+ + +	+ +	+
Portal/lobular inflammation	−	+ + + +	+ + +	+ + +	+

Absent (−), minimal (+), mild (+ +), moderate (+ + +), sever (+ +  + +).

C, control group; DEX, dexamethasone-treated group; DEX + LDE, dexamethasone + low dose extract; DEX + HDE, dexamethasone + high dose extract; DEX + M, dexamethasone + metformin (Reference drug).

#### Histopathological evaluation of pancreatic tissue

**Fig. 10:**
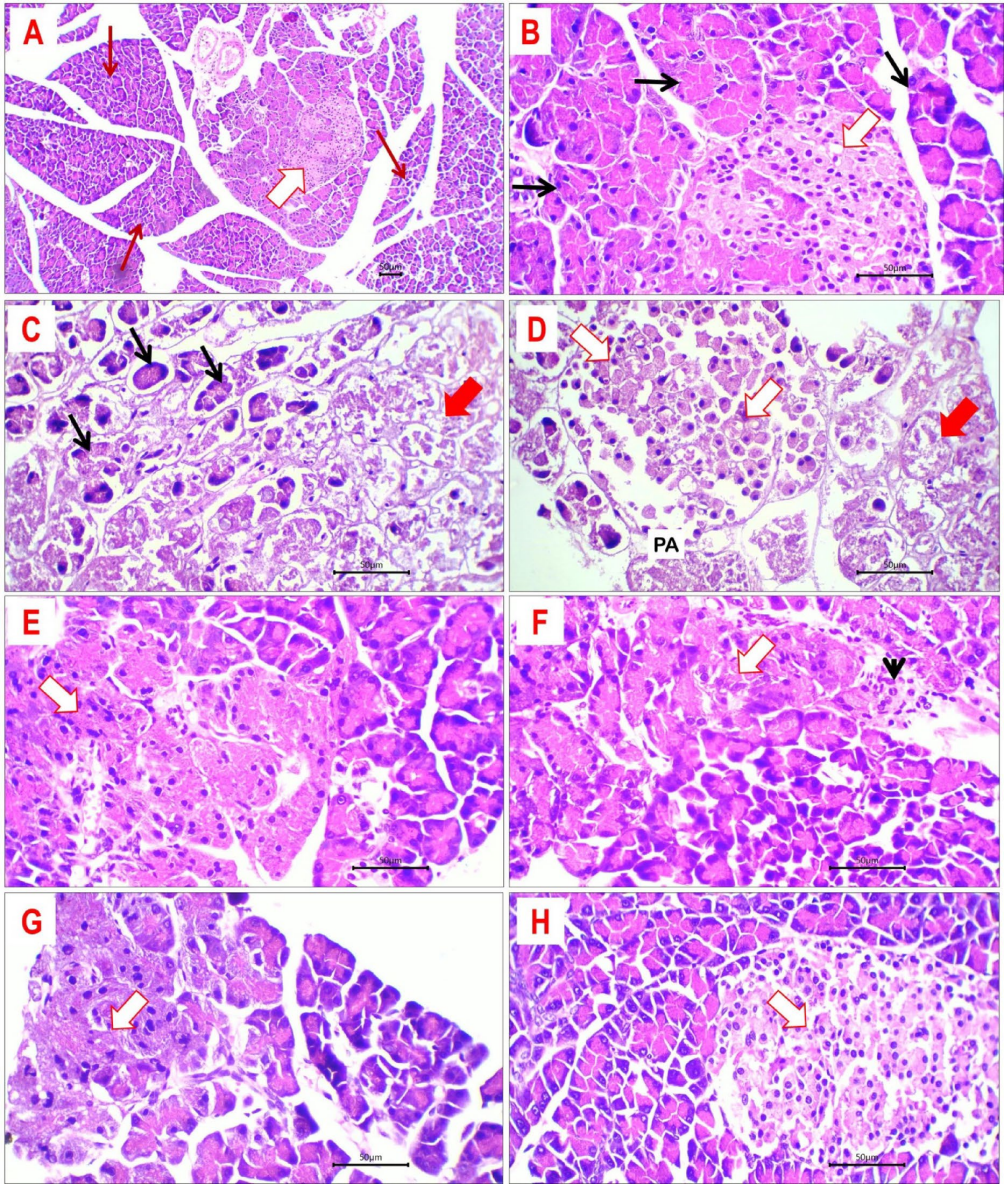
Histological sections of pancreatic tissue from different study groups: (**A**, **B**) control group showed normal pancreatic lobules (red arrows) and identified islets` cells (white arrow). Pancreatic acini have uniform size and shape with uniform cuboidal cell lining (black arrows). (**C**, **D**) pancreatic tissue of DEX-treated rats showed extensive necrosis of exocrine (red arrows) and endocrine (white arrow) component with residual degenerated exocrine pancreatic acini (black arrows). **E*:*** DEX + M; pancreatic tissue of M treated rats with degenerated islet`s cells (white arrow). (**F**, **G**) pancreatic tissue of DEX-LDE-treated rats with degenerated/hyalinized islet`s cells (white arrow) and residual stromal inflammation (arrow head. **H** DEX + HDE induced improvement of pancreatic tissue with retained exocrine and endocrine (white arrow) components. H&E-stained sections; magnification is × 100 for A and × 400 for others.

Sections of pancreatic tissue from the control group displayed preserved lobular architecture, with distinctly delineated exocrine and endocrine secretory components (Fig. 10A,B). The exocrine pancreatic acini exhibited homogeneous size and shape, encased by a monolayer of cuboidal cells characterized by eosinophilic cytoplasm with nuclei consistently located at the base. Numerous diminutive clusters of insulin-secreting islet cells were identified, distinguished by their oval morphology, pale granular cytoplasm, and homogeneous basal nuclei. The islet cells are interlaced with a fine network of capillaries. The administration of DEX caused significant damage and necrosis to both exocrine and endocrine pancreatic tissues. Necrotic regions manifested as formations of homogeneous eosinophilic tissue devoid of cellular or nuclear characteristics. Necrotic regions are locally encircled by areas of inflammatory response, predominantly consisting of histiocytes and lymphocytes. Foci of deteriorated acini and degraded islet cells were seen. The cells exhibit eosinophilic cytoplasm and pyknotic tiny nuclei (Fig. 10C,D).

Treatment with M, LDE, and HDE led to substantial enhancement in pancreatic tissue, with minimal necrosis seen (Fig. 10E–H). The pancreatic tissue from rats in all three treatment groups exhibited intact normal pancreatic lobulation and distinct identification of both exocrine and endocrine components. In the rats administered M and LDE, there was significant selective degeneration of islet cells (Fig. 10E,F). Conversely, the pancreas of HDE-treated rats had consistently viable islet cells characterized by pale eosinophilic cytoplasm and centrally positioned uniform nuclei (Fig. 10H). The key histological changes in pancreatic tissue among different groups are detailed in Table 6.

**Table 6:** Histopathological findings of pancreatic tissues in different groups.

Parameter	Study groups				
	C	DEX	DEX + M	DEX + LDE	DEX + HDE
Necrosis/degeneration of exocrine pancreas	−	+ + + +	−	−	−
Necrosis/degeneration of endocrine pancreas	−	+ + + +	+	+	−
Inflammation	−	+ +	+	+	−
Hyalinosis	−	−	+	+	−

Absent (−), minimal (+), mild (+ +), moderate (+ + +), sever (+ +  + +).

C, control group; DEX, dexamethasone-treated group; DEX + LDE, dexamethasone + low dose extract; DEX + HDE, dexamethasone + high dose extract; DEX + M, dexamethasone + metformin (Reference drug).

### Immunohistochemical investigation

#### Expression of TNF-α

**Fig. 11:**
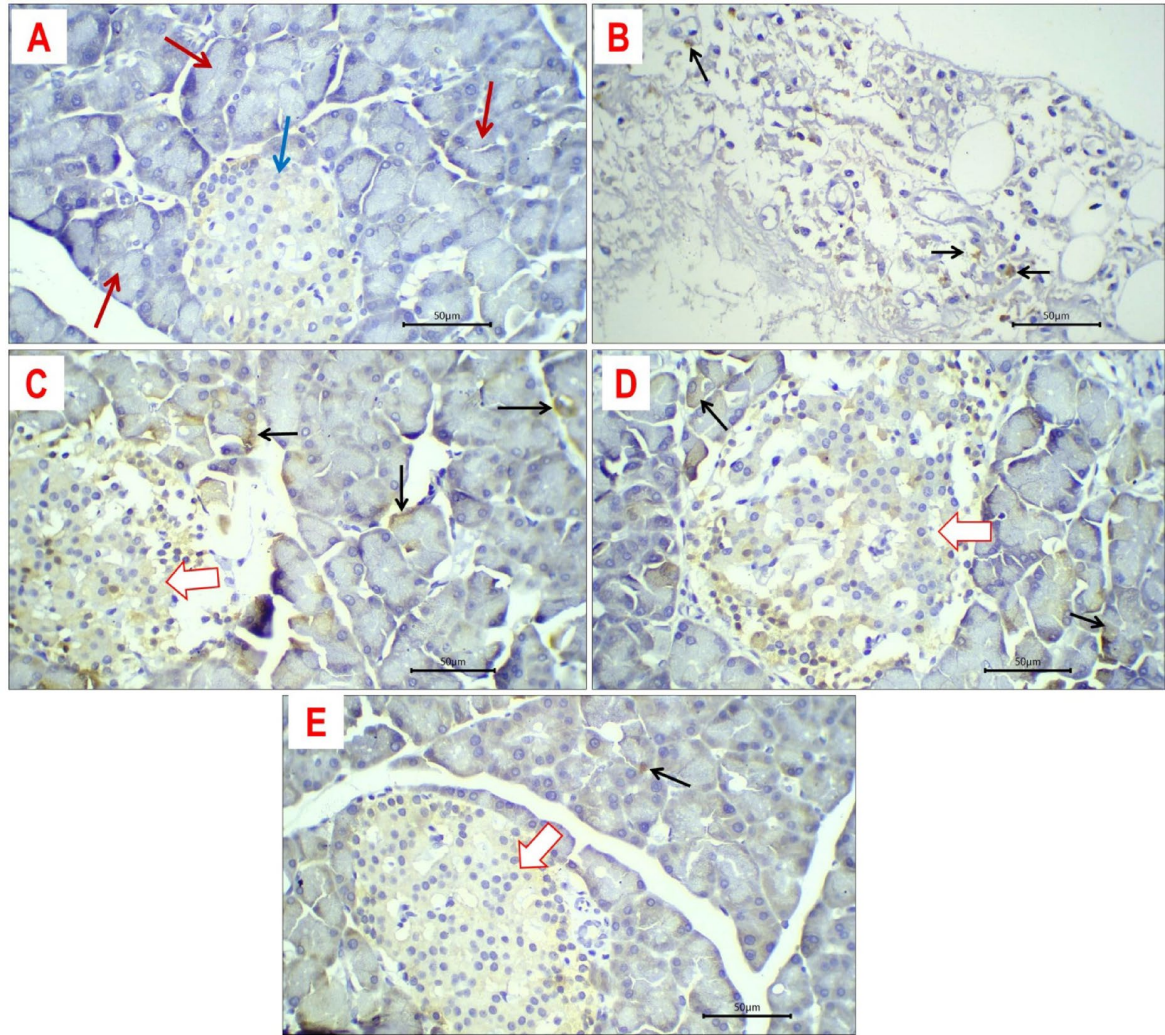
Expression of TNF in pancreatic tissue of different study groups: Negative expression of TNF in control rats (**A**) in exocrine (arrow) and endocrine (arrow) pancreatic cells. Cytoplasmic immune-staining of TNF in exocrine acini (black arrows) and islets` cells (white arrows) of pancreatic tissue obtained from DEX-treated rats (**B**) DEX + M-treated rats (**C**), DEX + LDE- treated rats (**D**) and DEX + HDE-treated rats (**E**). Immune-stained sections; magnification is × 400 for all.

The expression of TNF-α was identified as a brown, granular staining within the cytoplasm. Typically, TNF-α expression is minimal or subtle in the majority of positive cells throughout the examined groups (Fig. 11). Moreover, TNF-α concentrations were marginally increased in islet cells relative to exocrine pancreatic acini. The control group displayed no TNF-α expression in the pancreatic tissue, encompassing both the endocrine and exocrine components. The average percentage of TNF-α expression in exocrine pancreatic acini for the DEX, DEX + M, DEX + LDE, and DEX + HDE-treated groups is 12%, 5%, 5%, and 1%, respectively. The islet cells of the endocrine component exhibit full necrosis in DEX, accompanied by a lack of TNF-α production in the necrotic (non-viable) cells. The mean percentage of TNF-α expression in the DEX + M, DEX + LDE, and DEX + HDE treatment groups is 10%, 9%, and 4%, respectively, (Table 7).

**Table 7:** Average percentage of TNF-α expression in both exocrine pancreatic acini and endocrine portion from different studied groups.

	Exocrine pancreatic acini (%)	Endocrine portion (%)
C	–	–
DEX	12	–
DEX + LDE	5	9
DEX + HDE	1	4
DEX + M	5	10

C, control group; DEX, dexamethasone-treated group; DEX + LDE, dexamethasone + low dose extract; DEX + HDE, dexamethasone + high dose extract; DEX + M, dexamethasone + metformin (Reference drug).

#### Expression of PCNA

**Fig. 12:**
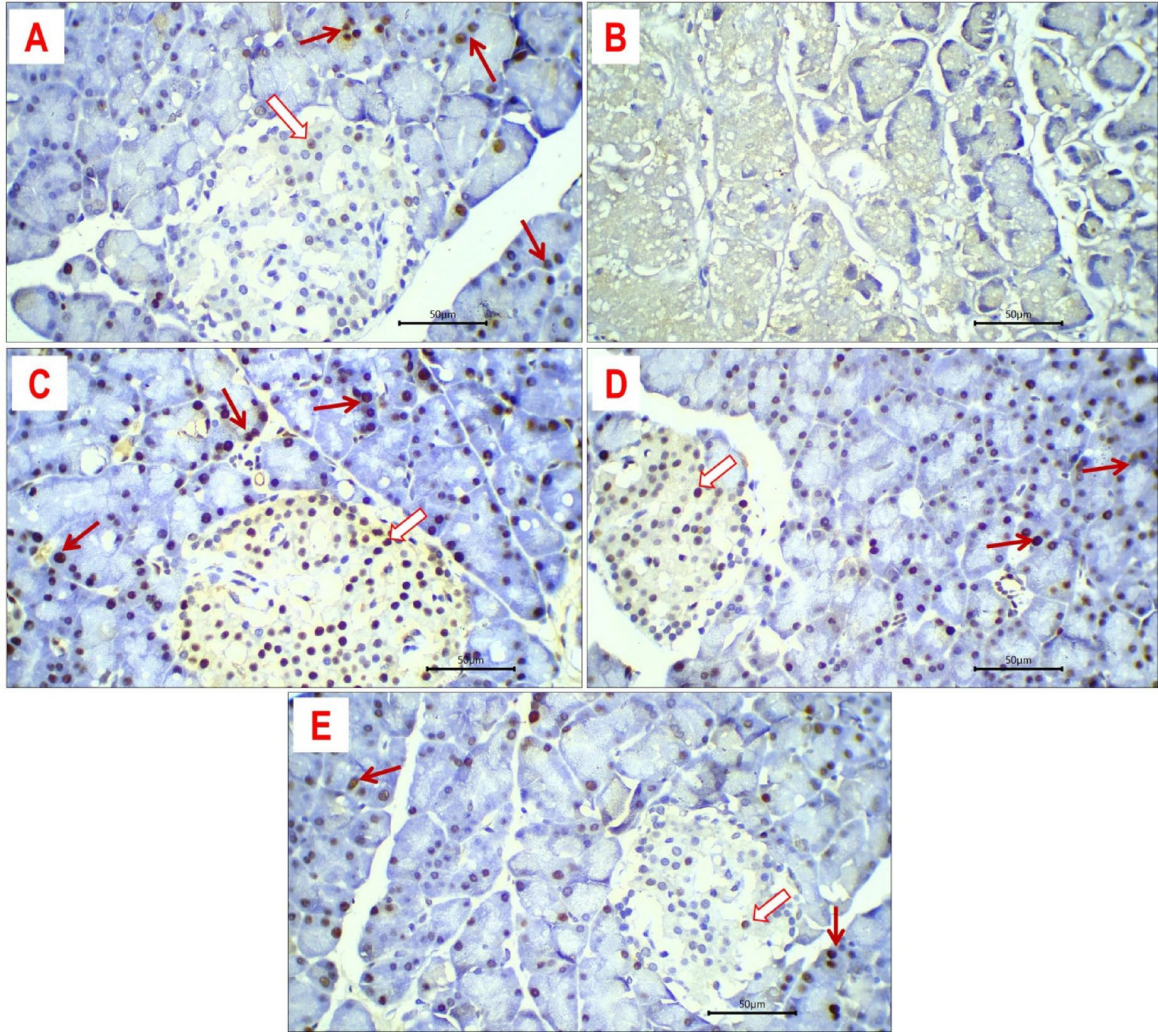
Expression of PCNA in pancreatic tissue of different study groups: Nuclear immune-staining of PCNA in exocrine acini (red arrows) and islet cells (white arrows) of pancreatic tissue obtained from control rats (**A**), DEX + M-treated rats (**C**), DEX + LDE-treated rats (**D**) and DEX + HDE- treated rats (**E**). Expression of PCNA in DEX-treated rats (**B**) was negative. Immune-stained sections; magnification is × 400 for all.

PCNA expression was identified as brown staining within the nucleus. Overall, PCNA expression was moderate to strong in the majority of positive cells across various study groups (Fig. 12). Moreover, both exocrine acini and islet cells demonstrated PCNA expression, with levels significantly elevated in the exocrine pancreatic acini in comparison to the islet cells. In the various experimental groups, DEX-treated rats had diminished PCNA expression in their pancreatic tissue due to extensive necrosis, signifying the absence of viable, proliferative cells. In control rats, the mean expression level of PNCA was 18% in exocrine acini and 6% in islet cells. In rats treated with DEX + M, DEX + LDE, and DEX + HDE, the average expression levels of PCNA were 45%, 25%, and 20% for exocrine pancreatic acini, respectively, and 30%, 20%, and 5% for endocrine islet cells, respectively.

## Discussion

In the present investigation, DEX injection induced insulin resistance, as evidenced by hyperglycemia, hyperinsulinemia, elevated HOMA-IR index, hypercholesterolaemia, hypertriglyceridemia, diminished insulin sensitivity index, and reduced GLUT4 expression in skeletal muscles. Geer et al. indicated that DEX treatment may promote insulin resistance in rats^23^. One hypothesized mechanism for the creation of insulin resistance by DEX is the inhibition of hepatic hexokinase, a reduction in hepatic glucose oxidation, and the activation of hepatic gluconeogenesis^24^. A potential mechanism by which DEX induces insulin resistance is the reduced expression of GLUT4 in both adipose tissue and skeletal muscle, leading to diminished glucose absorption and utilization, which results in observed hyperglycemia. Moreover, it has been shown that DEX elevated quantities of free fatty acids, potentially decreasing cell membrane expression of GLUT4, hence impairing cellular glucose absorption and diminishing its utilization in glucose disposal organs^25^. Insulin resistance in skeletal muscle is recognized as the principal problem, frequently emerging decades prior to the development of β-cell failure and notable hyperglycemia^26,27^. Growing data suggests that GLUT4 is essential for glucose uptake in skeletal muscle cells, with defective GLUT4 translocation being the primary abnormality linked to insulin resistance in these cells^28–30^. Therefore managing insulin resistance in skeletal muscle alone is sufficient to maintain systemic glucose homeostasis^27,31^.

The findings of this study indicated that HDE administration significantly increased GLUT4 expression and the insulin sensitivity index compared to M, the reference medication utilized in this work. Conversely, HDE exhibited a notable reduction in fasting insulin levels and HOMA-IR compared to M.

UPLC-MS/MS facilitated the identification of many flavonoids in the extract of *C. proximus*. The predominant subclass of flavonoids was flavones, with apigenin, luteolin, and tricin glycosides identified as the principal derivatives. Flavonoids were found to exert anti-diabetic benefits through many methods, including enhancing the capacity of insulin-resistant cells to use glucose and synthesize glycogen by activating GLUT4 and phosphorylating glycogen synthase kinase 3β (GSK-3β). Furthermore, they modestly elevated the expression levels of insulin receptor substrate-1 (IRS-1) and insulin receptor substrate-2 (IRS-2) in these resistant cells, in conjunction with the phosphatidylinositol 3-kinase (PI3K)/protein kinase B (Akt) signaling pathway^32^. Moreover, they enhance the expression of GLUT4 on the plasma membrane, elevating adipocyte GLUT4 and hepatic glucokinase levels via the activation of peroxisome proliferator-activated receptor gamma (PPAR γ)^33^. Furthermore, flavonoids inhibited phosphoenolpyruvate carboxykinase (PEPCK) and glucose-6-phosphatase^34^.

Phenolic acids were shown to enhance insulin secretion, block α-amylase and β-glucosidase activity, obstruct the sodium-dependent glucose transporter-1 in the intestine from absorbing glucose, and diminish hepatic glucose production. Furthermore, it facilitates insulin-dependent glucose uptake and stimulates adenosine monophosphate-activated protein kinase^35,36^. In addition, phenolic acids inhibit phosphoenolpyruvate carboxykinase and glucose-6 phosphatase, and controls β-cell and adipocyte GLUT4 activities, all of which contribute to managing diabetes mellitus^37^.

Anthocyanins are among the most crucial polyphenols in the management of diabetes^38^. They can inhibit PTP1B activity and promote glycogen synthesis through the IRS-1/PI3K/Akt/GSK3β pathways^39^. Moreover, anthocyanins have demonstrated efficacy in inhibiting the α-amylase and β-glucosidase enzymes, hence reducing postprandial glycemia^40^.

Coumarin derivatives were identified as potential treatments for diabetes and related complications due to their ability to repair pancreatic β-cells and improve insulin signaling^41^. They augment Akt and GSK-3β phosphorylation in C2C12 cells. Consequently, raises the possibility that the antihyperglycemic effect of coumarins may be associated with insulin sensitization^42^. Furthermore, coumarins enhance insulin secretion in rat insulinoma (INS-1) cells, indicating its potential as a diabetic treatment. Vinayagam and Xu assert that coumarin’s antioxidant and anti-apoptotic capabilities in INS-1 cells enhance its anti-diabetic benefits^43^.

Stilbenes (1,2-diarylethens), a significant category of naturally occurring polyphenolic chemicals, are recognized for their various biological actions^44,45^. These natural substances can prevent, mitigate, or reverse diabetes dysfunction via various pathways and molecular targets. Also, they inhibit α-amylase and α-glucosidase, enzymes that hydrolyze carbohydrates, so enhancing glycemic management alongside their antioxidant and anti-inflammatory qualities, thereby mitigating the effects of diabetes and its complications^46^.

According to our results; insulin resistance induced modifications in the lipid profile. These findings were consistent with the prior study conducted by Wego et al^10^. Insulin resistance increases the activity of hormone-sensitive lipase in adipose tissue while diminishing the activity of lipoprotein lipase. This results in enhanced mobilization of fatty acids from adipocytes and increased triglyceride synthesis in the liver, subsequently released into the circulation as VLDL cholesterol^47^. The present study indicates that the extract and M improved these changes in lipid metabolism. The results may indicate that *C. Proximus* extract has the potential to augment tissue sensitivity to insulin, hence diminishing the activity of hormone-sensitive lipase and elevating the activity of lipoprotein lipase. This indicates that the extract possesses hypolipidemic characteristics. The hypolipidemic effect of the extract may be ascribed to the presence of flavonoids. The former amplifies the elevation of fecal sterols, which subsequently results in a reduction of cholesterol absorption from dietary sources. Furthermore, an alternative mechanism for the cholesterol-lowering impact of the extract may entail the stimulation of cholesterol metabolism through the modulation of enzymes involved in cholesterol processing. The enzymes encompass HMG CoA reductase, lecithin cholesterol acyltransferase, cholesterol 7α-hydroxylase, and acyl-CoA: cholesterol acyltransferase, which are modulated by flavonoids and phenolic substances^48^. Moreover, it is established that flavonoid consumption reduces LDL-C and elevates HDL-Cholesterol, so facilitating the transfer of cholesterol from peripheral tissues to the liver for excretion and catabolism^49^.

One modification caused by DEX injection is hepatotoxicity, evidenced by a notable increase in serum ALT and AST activity, along with a considerable reduction in albumin levels. The results were consistent with prior research conducted by Hasona et al.^50^. Furthermore, the hepatotoxicity generated by DEX was indorsed by significant histological changes (Fig. 9B,C). The administration of both extract dosages and M substantially mitigated these biochemical and histological changes. The HDE shown greater improvement than the other treatments and successfully restored the normal liver tissue architecture, highlighting the significance of *C. proximus* extract in mitigating DEX-induced liver damage (Table 4). DEX administration elicited an oxidative stress. These results were consistent with a prior work by Hasona et al.^50^. The author indicated that oxidative stress is a primary cause of DEX-induced hepatotoxicity due to the excessive production of free radicals. The administration of the extracts and M substantially mitigated these anomalies. In this situation, the HDE demonstrated more significant effects compared to the LDE and M.

The hepatoprotective and antioxidant benefits of the extract are likely due to its abundant polyphenols and flavonoids, which are essential metabolites with antioxidant characteristics. These chemicals can stabilize and retain liver cell membrane integrity, increase hepatocyte regeneration, repair damaged and necrotic tissues, and enhance enzymatic activity and protein production inside hepatocytes^51^.

Flavonoids have demonstrated the ability to augment the production of proteins, including heme oxygenase 1 (HO-1) and glutamate cysteine ligase (GCL), together with its catalytic (GCLC) and modifier (GCLM) subunits. They also elevate intracellular glutathione (GSH) levels and boost the ratio of GSH to oxidized GSH. Furthermore, flavonoids can activate extracellular signal-regulated protein kinase 2 (ERK2), facilitate the nuclear translocation of nuclear factor erythroid 2-related factor 2 (Nrf2), and enhance the binding affinity of nuclear Nrf2 to the antioxidant responsive element (ARE).^52^.

Furthermore, coumarins have been widely utilized as alternative and complementary therapies. They have demonstrated the capacity to trigger Nrf2 signaling in many cells and experimental organisms. Moreover, coumarins can mitigate oxidative stress through their capacity to scavenge reactive oxygen species (ROS) and prevent neutrophil-mediated superoxide anion production and lipid peroxidation^53^.

Various chronic inflammatory and immune-mediated diseases have been shown to develop insulin resistance. Moreover, patients with T2DM have an increased levels of inflammatory markers in their bodies^54^. Moreover, TNF-α has been identified as a crucial factor in disrupting the insulin signaling system and promoting insulin resistance. It affects both the insulin receptor and the insulin receptor substrate (IRS), leading to a reduced response to insulin signals^55^. Moreover, TNF-α may elevate the levels of cytokine signal suppressors, which subsequently form complexes with insulin receptor substrates (IRS1 and IRS2), resulting in cellular damage. This mechanism negatively impacts insulin’s capacity to promote glucose uptake in adipose and muscle cells, ultimately leading to increased blood glucose levels^56^.

In the current work, the immunohistochemistry analysis revealed that the highest percentage of TNF-α expression was observed in the pancreatic tissue of the DEX-treated group. Conversely, the DEX-HDE group exhibited the lowest percentage of TNF-α expression, suggesting that the extract demonstrates considerable anti-inflammatory efficacy in this diabetic paradigm. These data indicate that *C. proximus* extract can reduce fasting blood glucose and insulin levels, while enhancing insulin sensitivity via decreasing TNF-α production levels. The anti-inflammatory properties of *C. proximus* extract are ascribed to the presence of flavonoids^17^, coumarins^57^, and stilbenes^44^ which are known for their several health benefits, (Table 1).

PCNA is a nuclear non-histone protein that plays a crucial function in DNA replication and repair processes. Its expression may serve as an indicator of cell proliferation, as cells remain in the G1/S phase for an extended duration during proliferation^58^. The present study indicates that PCNA expression in DEX + HDE is comparable to the control values, signifying preserved normal pancreatic lobulation and the presence of both exocrine and endocrine components^55,59^. The histological data corroborated this discovery, revealing that the pancreas of HDE-treated rats had uniformly viable islet cells characterized by pale eosinophilic cytoplasm and centrally located uniform nuclei (Fig. 12). Further investigation is necessary to elucidate the underlying mechanisms of the identified compounds.

## Conclusion

The results of this study indicate that *C. proximus* extract exhibits insulin-sensitizing properties in DEX-induced insulin-resistant rats via increasing GLUT4 levels in skeletal muscles and promoting islet cell regeneration. Human trials and mechanistic studies are recommended to investigate the efficacy of the extract in treating insulin resistance by evaluating fasting blood glucose, insulin levels, and HOMA-IR.

## Materials and methods

### Chemicals and drugs

The chemicals utilized in this work were of analytical grade and purchased from Sigma-Aldrich (St. Louis, MO, USA). Dexamethasone sodium phosphate injection was procured from Amriya Company for Pharmaceutical Industries, Alexandria, Egypt. M hydrochloride powder was acquired from MUP Pharmaceutical Company located in Ismailia, Egypt.

### Plant material

*C. proximus* (Hochst. ex A. Rich.) The aerial parts of Chiov (family: Poaceae) were purchased in a nearby Egyptian market. Prof. Dr. Abd El-Halim Abdel-Motjale, Chief Research Officer and Head of the Department of Flora and Phytotoxonomy Research at the Agriculture Museum, kindly authenticated the sample. The Herbarium of the Pharmacognosy and Medicinal Plants Department, Faculty of Pharmacy (for women), Al Azhar University, Cairo, Egypt, has voucher specimens (Cp, 2022).

### Plant extraction

The plant material was ground into a fine powder, yielding 300 g, and thereafter immersed in 70% MeOH for four cycles of 3L at ambient temperature (32 °C), with intermittent agitation. The solution was further filtered employing Whatman filter paper No. 1. A rotary evaporator (Buchi Co., Switzerland) was employed at a temperature of 50 °C to evaporate the filtrate, yielding a brown crude extract weighing 100 g. Multiple metabolites were provisionally identified in the extract following the exposure of 50 mg to UPLC-ESI–MS/MS analysis in both positive and negative modes.

### UPLC-ESI-QTOF-MS/MS analysis

The proteomics and metabolomics team at the Children’s Cancer Hospital Egypt (57357) in Cairo conducted the LC/MS study. A quadruple time-of-flight (QTOF) mass spectrometer (Triple TOF 5600+, SCIEX) was integrated with a standard HPLC interface (Exion LC, AB SCIEX, Concord, Canada) utilising a stock solution of the produced extract, which was instrumented and acquired for data processing. As per the preceding description^19,60,61^, The operation of the instrument, compilation of data, and processing of data were executed. Metabolite classes were identified by comparing retention periods, mass spectrometry data, and fragmentation patterns in both ion modes with the ReSpect-positive database (2737 records), ReSpect-negative database (1573 records), and previously published literature^62^.

### Animals

Male Wistar albino rats, aged 10 to 11 weeks and weighing 160 to 180 grammes, were utilized in this work. The rats were acquired from the animal facility at the Faculty of Science, Sohag University. They were accommodated in an environment with a humidity level of 50–55%, a temperature regulated at 25 ± 1 °C, and a light–dark cycle of 12 h, with illumination commencing at 0600 h. The rats were provided with unimpeded access to tap water and a conventional pellet diet. All experimental protocols received approval from the Institutional Animal Care and Use Committee (IACUC) of the Clinical Pharmacology department, Faculty of Medicine, Sohag University (Approval no. 5-5-2/2024-01), and adhered to the rules established by the National Institutes of Health. All methods were conducted in compliance with ARRIVE standards.

### Experimental design

Thirty-five (35) rats were weighed prior to the commencement of the experiment and thereafter classified into five groups of seven animals each. Animals had a 14-h overnight fast prior to dexamethasone administration, as per Mahendran and Devi^63^. The groups were classified as follows:

*Group 1*: served as normal control and kept on distilled water.

*Group 2: Dexamethasone-treated (DEX)*: received an intraperitoneal injection of DEX at a dose of 1 mg/kg^63^ for 14 days and served as an insulin-resistant group.

*Group 3: Dexamethasone + low dose of the extract-treated group (DEX + LDE)*: received an intraperitoneal injection of DEX at a dose of 1 mg/kg^63^, for 14 days. After the termination of DEX injection, rats were orally administered 100 mg of the extract /kg of body weight (according to El-Nezhawy et al.)^64^ by oral gavage by for another 14 days.

*Group 4: Dexamethasone + high dose of the extract-treated group (DEX + HDE)*: received an intraperitoneal injection of DEX at a dose of 1 mg/kg^63^, for 14 days. After the termination of DEX injection, rats were orally administered 200 mg of extract /kg of body weight (according to El-Nezhawy et al.)^64^ by oral gavage for another 14 days.

*Group 5: Dexamethasone + M (DEX + M)*: received an intraperitoneal injection of DEX at a dose of 1 mg/kg^63^, for 14 days. After the termination of DEX injection, rats were orally administered 40 mg of M (reference drug) /kg of body weight (according to Ukwenya et al.)^65^ by oral gavage for another 14 days.

Following 28 days of treatment, the animals were weighed, and following a 12-h fast, blood samples were extracted from the retro-orbital vein of each rat using a glass capillary tube. The blood specimens were permitted to coagulate and were centrifuged at 4000 rpm for 15 min. The serum was collected and stored at − 80 °C until subsequent analysis. Animals were euthanized via cervical decapitation under light anesthesia; Sodium phenobarbital (150 mg/kg, i.p.) was administered, and the liver, pancreas, and skeletal muscles were excised.

### Biochemical analysis

Serum albumin, ALT, AST activity, glucose, total cholesterol, triglycerides, and HDL-Cholesterol were estimated with commercial kits from Biodiagnostics, Cairo, Egypt. LDL-Cholesterol was calculated using Friedewald’s formula: LDL-C = TC - HDL-C - TAG/5 (Friedewald 1972)^66^. Insulin level was assessed by using Rat Insulin (INS) Elisa kit, Catalogue Number: MBS724709, China, rat GLUT4 was evaluated by Elisa kit, Catalog No: E-EL-R0430, Elabscience, China. HOMA-IR is calculated using the formula: (fasting glucose [mmol/L)] × fasting insulin [mIU/L]/22.5), and the insulin sensitivity index was calculated according to Mokiran et al.^67^.

Reduced glutathione (GSH) was measured in rat tissues using the Elisa Kit Catalogue Number: E02G0367 from Shanghai BlueGene Biotech Co., Ltd, China. Rat superoxide dismutase (SOD) in tissues was determined using the Elisa Kit Cat. No: MBS036924, while rat Malondialdehyde (MDA) was measured with Elisa Kit Cat No. MBS268427.

### Histopathological studies

Liver and pancreas tissue samples from various groups were fixed in 10% neutral buffered formaldehyde for 24 h at ambient temperature for histopathological analysis. Post-fixation, the samples underwent dehydration with ascending concentrations of ethyl alcohol and were subsequently cleared with xylene at room temperature before being embedded in molten paraffin at 70ºC. After preparing paraffin blocks for each sample, 5 µm thick sections were de-paraffinized in xylene, rehydrated with descending concentrations of ethyl alcohol, and thoroughly washed in running tap water. For staining, the tissue sections were immersed in haematoxylin for 7 min, rinsed in running water, and then stained with eosin for 2 min. The sections were washed again in running tap water to remove excess dye, dehydrated with ascending concentrations of ethyl alcohol, cleared in xylene, and mounted using DPX. All staining procedures were performed at ambient temperature.

### Immunohistochemical assessment

#### TNF-α protein expressional assessment

TNF-α, a crucial cytokine and inflammatory mediator, is produced during inflammation. Immunohistochemical staining was employed to evaluate the expression pattern of TNF-α, utilizing a previously reported protocol with slight alterations. Formalin-fixed paraffin-embedded tissue blocks were sectioned into 5 μm thick serial slices. Phosphate-buffered saline was employed to rinse, rehydrate, and deparaffinize the sections. To suppress endogenous peroxidase activity, antigen retrieval was conducted utilizing the microwave method in a citrate buffer (pH 6.0), followed by a 20-min incubation in 3% H_2_O_2_. Following one hour at ambient temperature in a blocking agent, the sections were incubated overnight with TNF-α primary antibodies at 4°C. Following one hour of application of the secondary biotinylated antibody, slices were washed in phosphate-buffered saline and subsequently incubated for 45 min with streptavidin peroxidase. Subsequently, slices were counterstained with haematoxylin and diaminobenzidine (DAB), and the results were examined under a light microscope^68^.

### PCNA expressional assessment

After dewaxing, the pancreas specimen sections were treated for 30 min with a 1:100 dilution of a monoclonal antibody against mouse PCNA (Dako A/S, Grostrup, Denmark). The avidin–biotin complex method was employed to stain the specimens after three washings. The quantity of pancreatic cells exhibiting PCNA staining was enumerated to evaluate pancreatic proliferation. By counting more than 1000 pancreatic cells in each specimen, the PCNA labeling index which is equal to the number of stained pancreatic cells divided by the total number of stained plus unstained pancreatic cells was determined^69^.

### Statistical analysis

Data were presented as a mean ± standard error of the mean (SEM). Statistical analysis was conducted utilizing one-way Analysis of Variance (ANOVA) succeeded by the Tukey multiple comparison test. *P* values below 0.05 were considered significant. Data analysis was conducted with SPSS software version 23.0.

## Electronic supplementary material

Below is the link to the electronic supplementary material.

Supplementary Material 1

## Data Availability

Data is provided within the manuscript and supplementary information files.
