# Supplementary material for: Cymbopogon proximus Chiov’s extract improves insulin sensitivity in rats with dexamethasone-induced insulin resistance and underlying mechanisms
Source: Sci Rep. 2025 Jun 20;15:20096. doi: 10.1038/s41598-025-02340-0 (PMC12181259; doi:10.1038/s41598-025-02340-0)
Supplement: Supplementary file 1 — Supplementary Material 1 [file 41598_2025_2340_MOESM1_ESM.docx]

**Supplementary 1:**

**UPLC-ESI-MS/MS analysis of bioactive compounds in *C. proximus* extract**

**1) Flavonoids**

The major identified apigenin flavone is 8-C-xylosyl-6-C-glucosyl apigenin that revealed [M-H]^−^ at m/z 563.1409 and [M+H]^+^ at m/z 565.1567 for the molecular formula C_26_H_28_O_14_ and prominent fragment at 473.11195 [M−H−C_3_H_6_O_3_]^−^ and 433.1017 [M−H−C_5_H_6_O_4_]^−^. While the most abundant luteolin flavone is isoorientin-2''-O-rhamnoside which showed [M-H] ^−^ at m/z 593.1537 and [M+H] ^+^ at m/z 595.1686 for the molecular formula C₂₇H₃₀O₁₅ and outstanding fragments at 449.1081 [M+H−rha]^+^, 431.0989 [M+H−rha−H2O]^+^, 395.0806 [M + H − 2H_2_O − C_2_H_4_]^+^, 353.0635 [M + H − 2H_2_O − 2CH_2_O]^+^, 329.0662 [M+H−rha−C_4_H_8_O_4_]^+^, 299.0558 [M+H− rha−C_5_H_10_O_5_]+, 287.0549 for aglycone.

As an illustration of luteolin di-C-glycosides, a carlinoside with the chemical formula C_26_H_28_O_15_ produced the [M-H]^−^ ion at m/z 579.1692. Beyond 561.0975 [M-H-H_2_O]^-^, 519.1299 [M-H-60]^-^, 489.1029 [M-H-90]^-^, 459.0943 [M-H-120]^-^, 417.1059 [M-H-162]^-^ (-hexose), it displayed the typical di-C-glycoside fragments at m/z 369 [Agl+83]^−^ and m/z 399 [Agl+113]^−^ [^1^](#_ENREF_1).

Tricin aglycone was the most prevalent among its derivatives. It is present in both ionization modes as m/z 331.0813 for [M+H]^+^ and m/z 329.0648 for [M-H]^−^, with the molecular formula C_17_H_14_O_7_.

Kaempferol-3-*O*-rutinoside displayed a deprotonated molecular ion at *m*/*z* 593.1465 for the molecular formula C_27_H_30_O_15_ and a product ion at *m/z* 447.0982 and 285.0326, corresponding to kaempferol aglycone following the loss of 146 amu (a rhamnose) and 308 amu (a rutinose), respectively [^2^](#_ENREF_2).

naringenin presented the highest abundant flavanones followed by phlorizin. Naringenin gave a molecular ion peak [M – H]^−^ at m/z 271.1596 assigned to the formula C_15_H_12_O_5_ and prominent fragments at 151.1158 [M – H − C_8_H_8_O]^-^ that confirmed by peak at 273.1673 for positive ion mode with its fragment 153.1237 [^3^](#_ENREF_3).

The most prevalent isoflavone was daidzin, which displayed molecular ion peaks at m/z 417.1142 [M+H]^+^. Its distinctive fragments at m/z 137.1251, 199.0968, and 255.11548 account for the existence of ions [M+H-B-ring-CO] ^+^, [M+H-2CO]^+^, and daidzein aglycone[^4^](#_ENREF_4) .

cyanidin 3, 5-di-O-glucoside is the major anthocyanin found in the plant, followed by malvidin-3-O-glucoside. MS/MS of compound cyanidin 3, 5-di-O-glucoside gave, among the cyanidin aglycone ion at *m*/*z* 287, a fragmentation ion *m*/*z* 449, corresponding to the loss of 162 (hexose) from the molecular parent ion *m*/*z* 611.3603.

Malvidin-3-O-glucoside displayed a deprotonated molecular ion at *m*/*z* 491.1209 for the molecular formula C_23_H_25_O_12_ and a product ion at *m/z* 329.9082 malvidin aglycone following the loss of 162 (hexose). The existence of an aglycone fragment with the molecular ion 331.07843 at m/z 493.1345 in positive ion mode served as confirmation for this.

**2) Phenolic, carboxylic, and fatty acids**

Two sub-groups of phenolic acids, mostly classified as hydroxybenzoic and hydroxycinnamic acids, were identified by our research. Hydroxycinnamic acid derivatives, such as caffeic, ferulic, sinapic acids, or *p*-coumaric acid, are frequently produced by the interaction of quinic acid with certain trans-cinnamic acids. The greatest quantity of hydroxycinnamic acid in our extract was p-coumaric acid; it revealed a molecular ion peak [M + H]^+^ at m/z 165.0919 for the molecular formula C_9_H_8_O_3_ and major fragments at 119.08661 [M + H − HCOOH] ^+^ and 147.0787 [M + H − H_2_O]^+^. Additionally, it displayed a deprotonated molecular ion at m/z 163.0404 and a product ion at 119.0504 due to loss of CO2 [M-H-44]. Chlorogenic acid and caffeic acid were the next most common hydroxycinnamic acids. The presence of caffeic acid was confirmed by the observation of molecular ion peaks and mass fragments in both negative and positive modes. It has molecular ion peak at m/z 181.0882 [M+H]^+^ and fragment ion peaks at m/z 163 [M + H − H_2_O]^+,^ 135 [M + H − HCOOH]+, in the positive mode, while the observations of the mass peaks at m/z 179.0573 [M − H]^−^, 161 [M − H − H_2_O]^−^, and 150 [M − H − CO]^−^ in the negative mode mass confirmed the caffeic acid structure. Similarly, chlorogenic acid was proved in both modes, where it showed a deprotonated molecular ion at m/z 353.0876 and fragment ions at m/z 191 [M-H-162]^-^ and 179 [M-H-174]^-^ for quinic acid and caffeoyl moieties. While in positive mode, it showed [M + H]^+^ at m/z 355.1035 with fragment ions 163.0373 [M + H − C_7_H_12_O_6_]^+^, 145.0282 [M + H − C_7_H_12_O_6_ − H_2_O]^+^, 117.0306 [M + H − C_7_H_12_O_6_ − HCOOH]^+^.

Conversely, hydroxybenzoic acids share a C6-C1 structural element. Protocatechuic, vanillic, syringic, and p-hydroxybenzoic acids are the four types of hydroxybenzoic acids that are frequently encountered. Gallic acid was the most prevalentbenzoic acid, it showed [M + H]^+^ at m/z 171.0636 with fragment ions at 127.0352 [M + H − CO_2_]^+^, 125.9856 [M + H − HCOOH]^+^, 109.0275 [M + H − CO_2_ − H_2_O]^+^.

**Carboxylic acids** easily lose neutral fragments of CO (28   Da), H_2_O (18   Da), and CO_2_ (44   Da). Taking Succinic acid as an example, the large peak area with the quasimolecular ion m/z 117.0189 [M-H]^−^ and formula of C_4_H_6_O_4_ with fragment ions at m/z 99.0100 [M-H-H_2_O]^−^,  73.0301(BP) due to loss of a H_2_O and CO_2_ molecules, respectively. Malic acid was the second abundant one with precursor ion [M – H]^−^ at m/z 133.0143 assigned for C_4_H_6_O_5_ and daughter ions at 115.0038 (BP) [M-H-H_2_O] ^−^ and 71.0139[M-H-H_2_O-CO_2_] ^−^. Jasmonic acid, a polyunsaturated fatty acid, was the main fatty acid in *C. proximus*. It is designated as [M + H]^+^ at m/z 211.0945, and is the daughter ion of [M -44(CO2) ^+^ H]^+^ at 167.06361.

**3) Coumarins**

In accordance with their estimated masses, molecular formulae (MFs), and weights (MWs), coumarins, like all other phenolic kinds, displayed experimental molecular ion peaks. Five coumarins with both positive and negative ionization modes were included in the mass spectrum. According to peak area esculetin, is the most abundant coumarin, it exhibited a molecular ion peak at m/z 179.0339 [M+H]^+^ and its MS2 spectrum revealed an aglycone fragment ion at m/z 151.04105 [M+H-CO]^+^.

**Supplementary 2:**

**Mass spectra of bioactive compounds in *C. proximus* using LC-ESI-MS/MS**

|   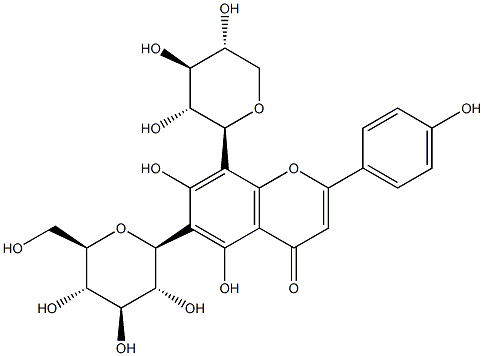  **Fig. A1 :** Mass spectra of 8-C-xylosyl-6-C-glucosyl apigenin |
| --- |
| ****  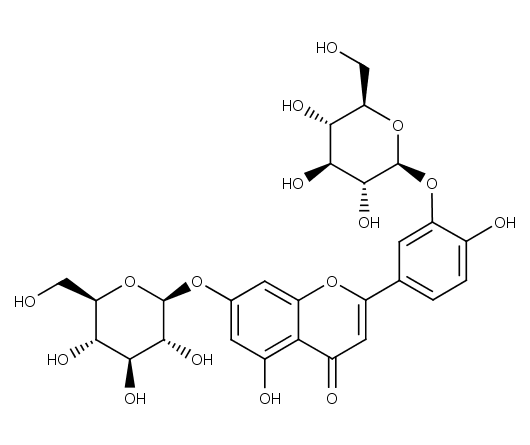  **Fig. A2 :** Mass spectra of Luteolin 3', 7-di-O-glucoside |
|   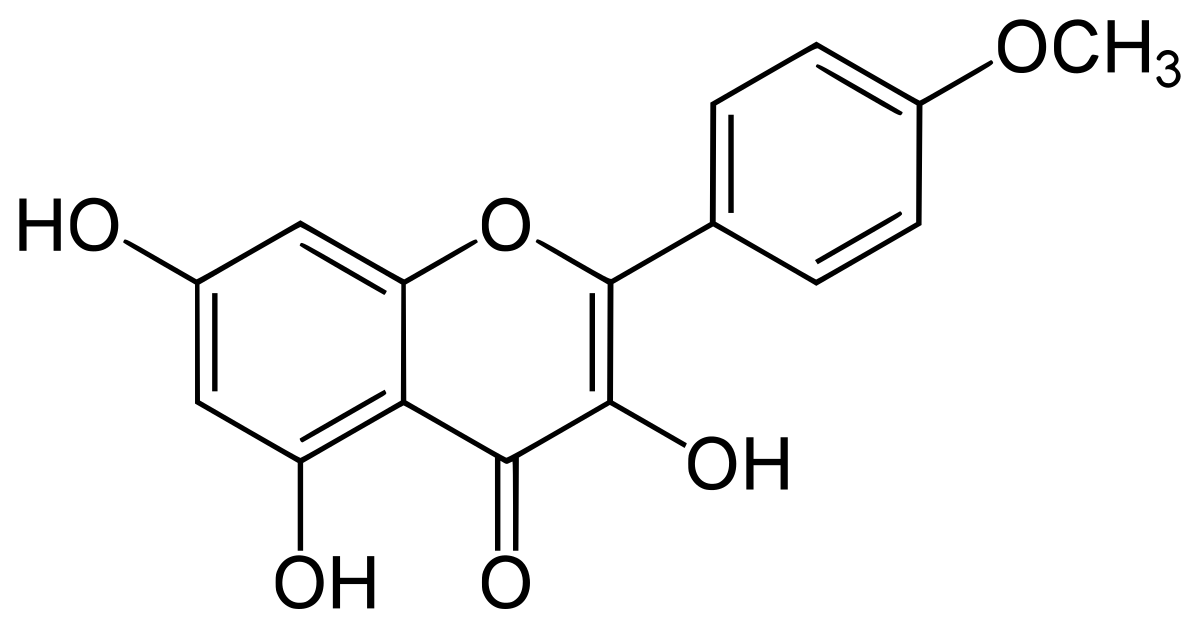  **Fig. A3 :** Mass spectra of Kaempferide, |
|   **Fig. A4 :** Mass spectra of Naringenin |
|   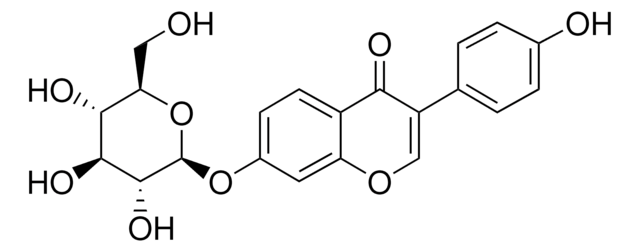  **Fig. A5 :** Mass spectra of Daidzin |
|   **Fig. A6 :** Mass spectra of Taxifolin |
|   **Fig. A7 :** Mass spectra of Cyanidin 3, 5-di-*O*-glucoside |
|   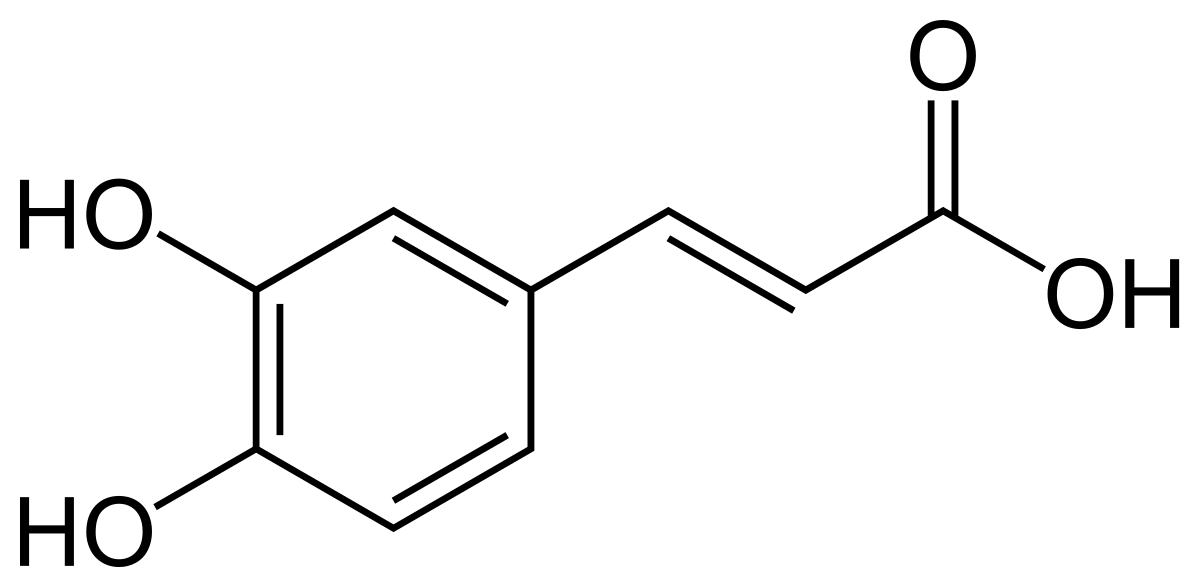  **Fig. A8 :** Mass spectra of Caffeic acid |
|   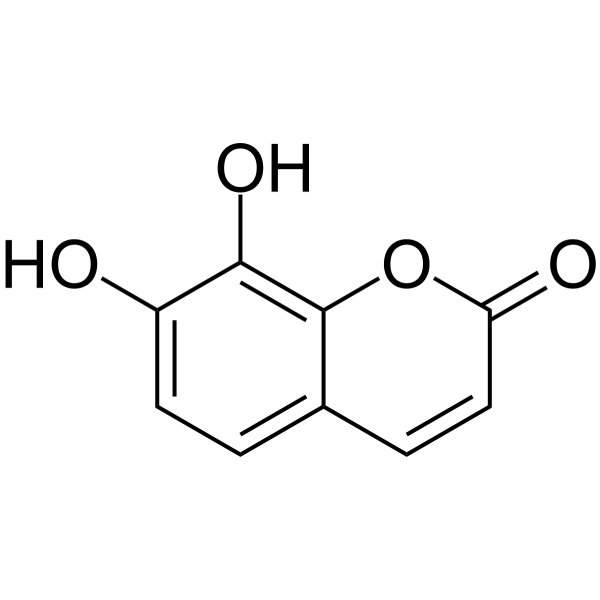  **Fig. A9 :** Mass spectra of Daphnetin |
|   **Fig. A10 :** Mass spectra of Astringin |

**Supplementary 3: Mass spectra of metabolites identified in *C. proximus* extract in both positive and negative ionization modes**

|   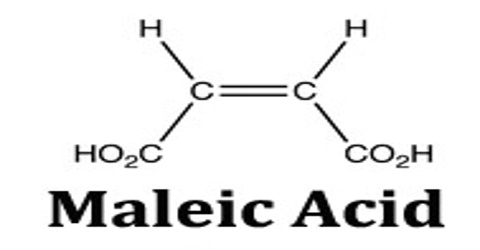  **Fig. S1 :** Mass spectra of maleic acid |
| --- |
|   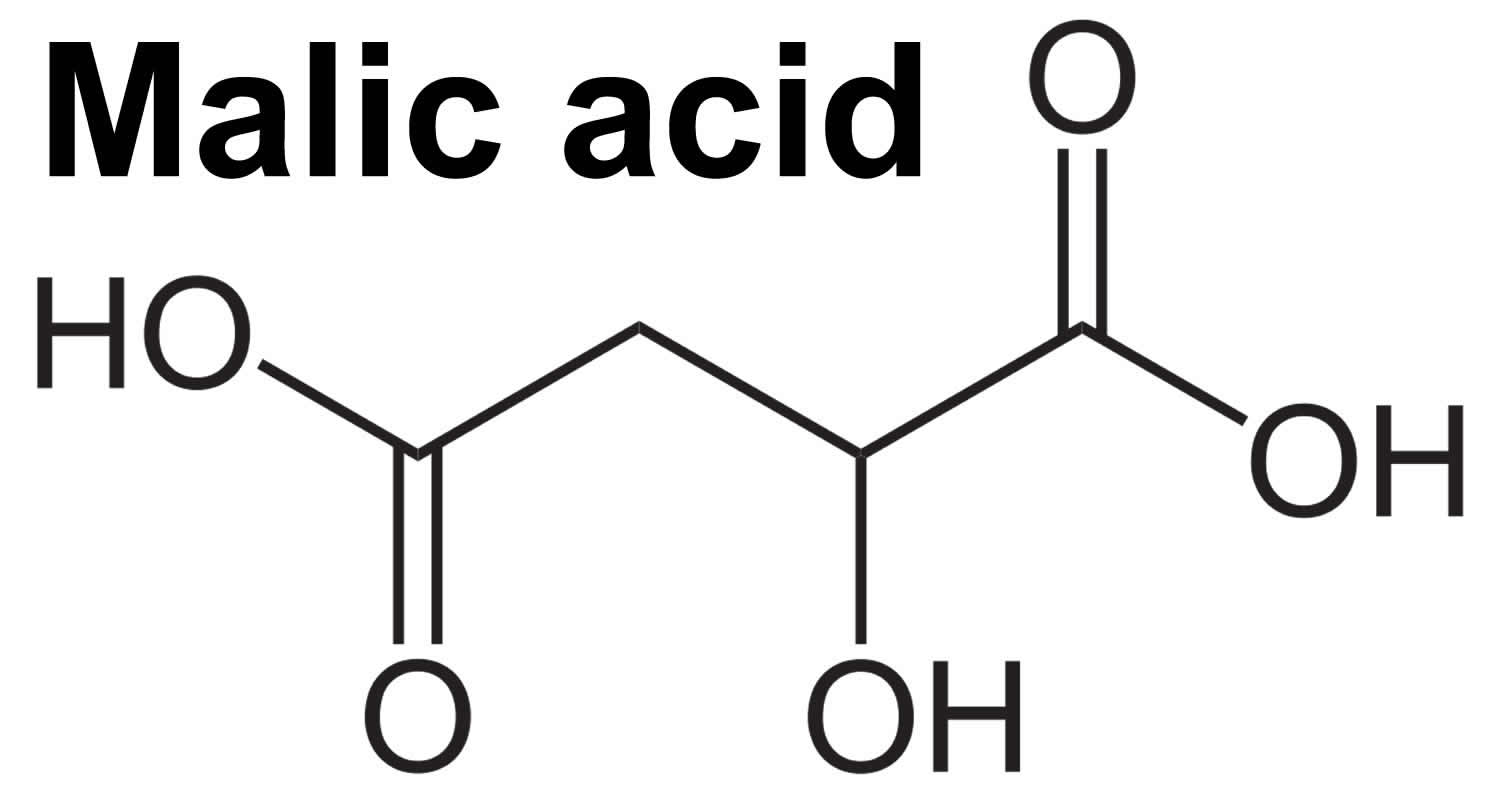  **Fig. S2 :** Mass spectra of malic acid |
|   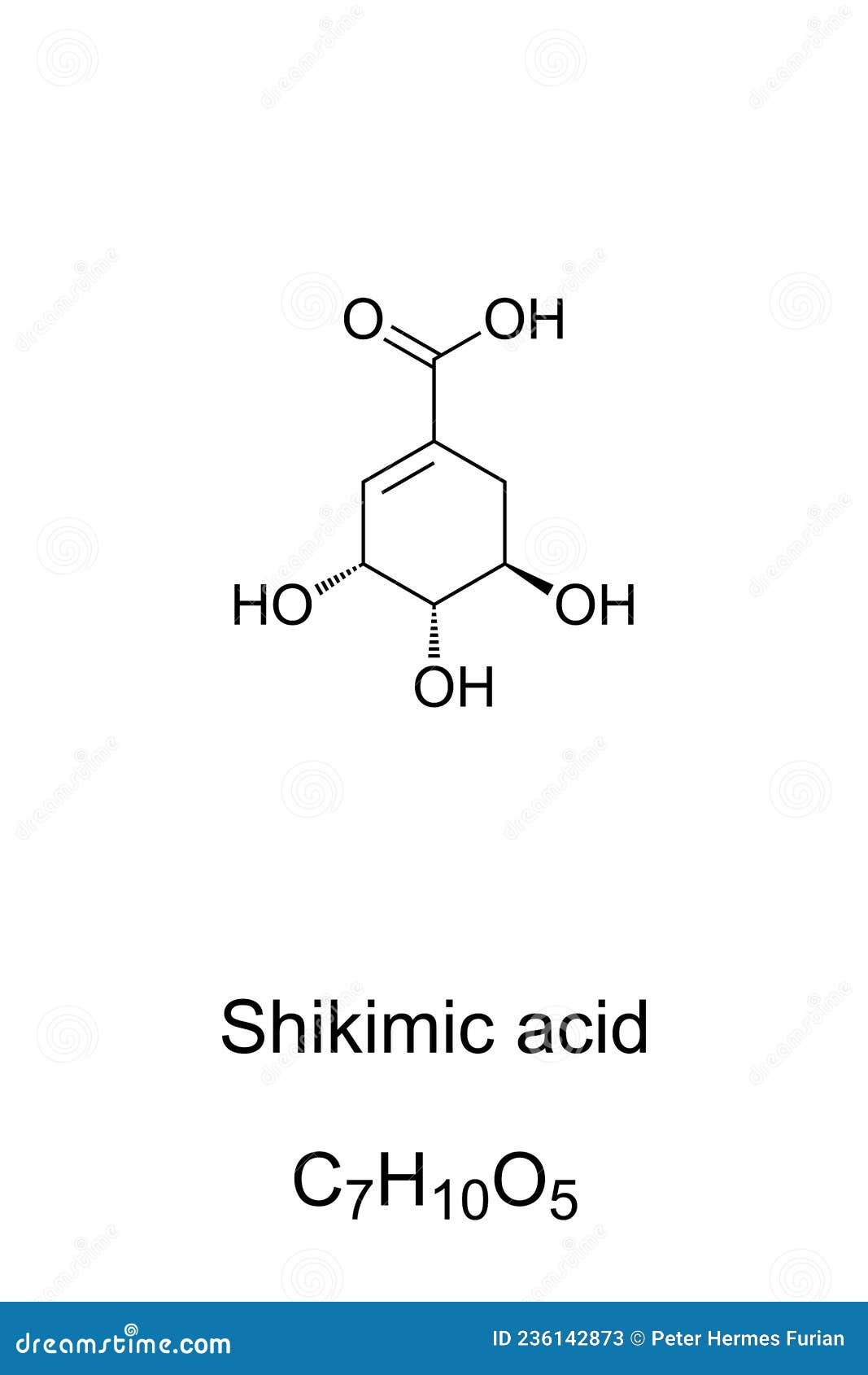  **Fig. S3 :** Mass spectra of shikimic acid |
|   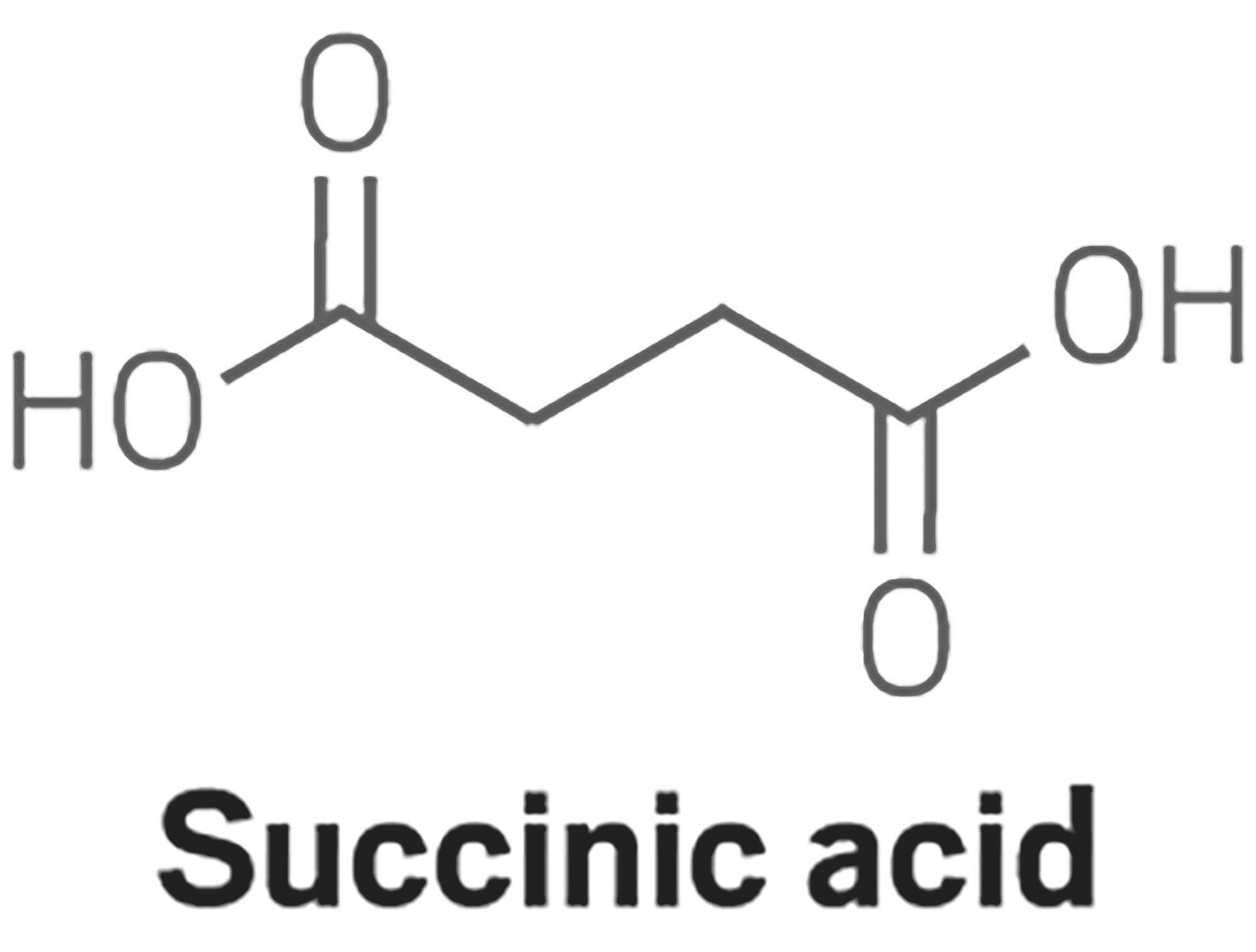  **Fig. S4:** Mass spectra of succinic acid |
|   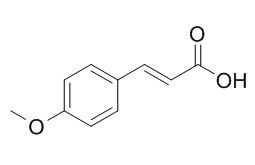  **Fig. S5 :** Mass spectra of 4-methoxycinnamic acid |
|   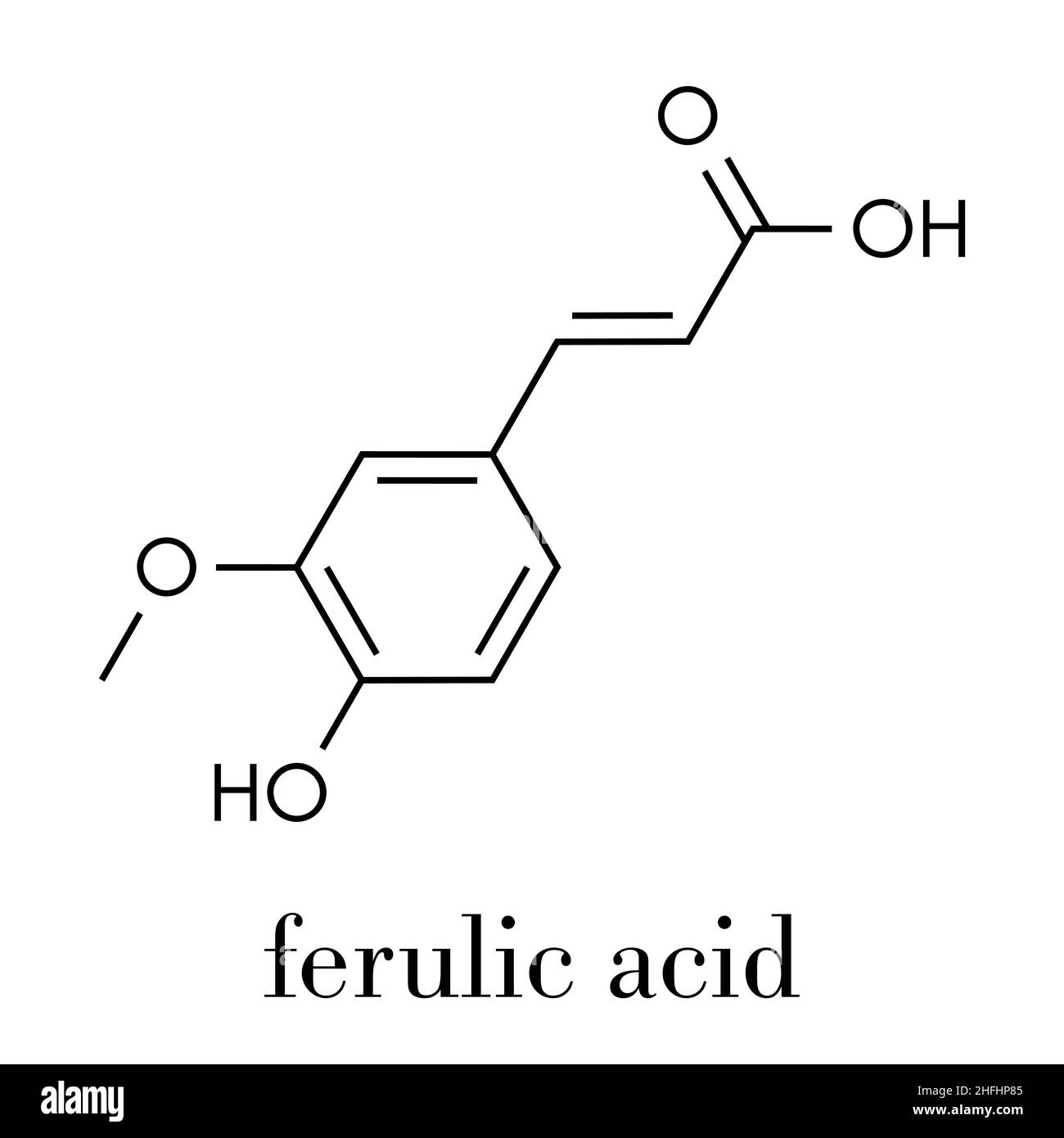  **Fig. S6 :** Mass spectra of ferulic Acid |
|   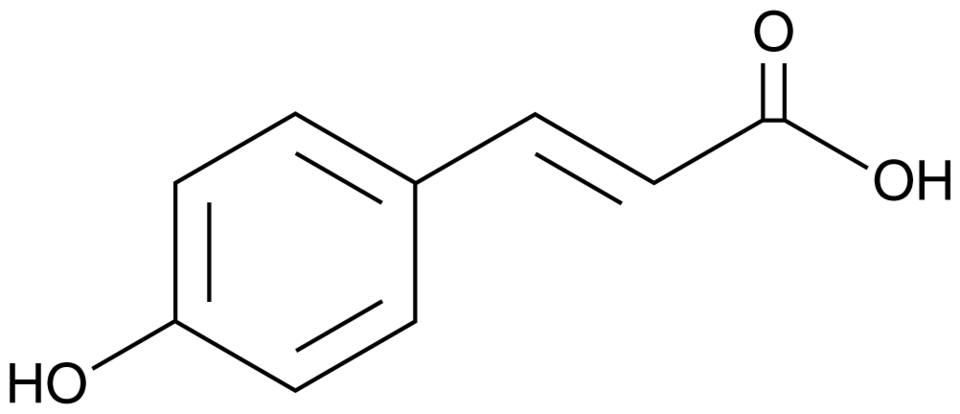  **Fig. S7 :** Mass spectra of *p*-Coumaric acid |
|   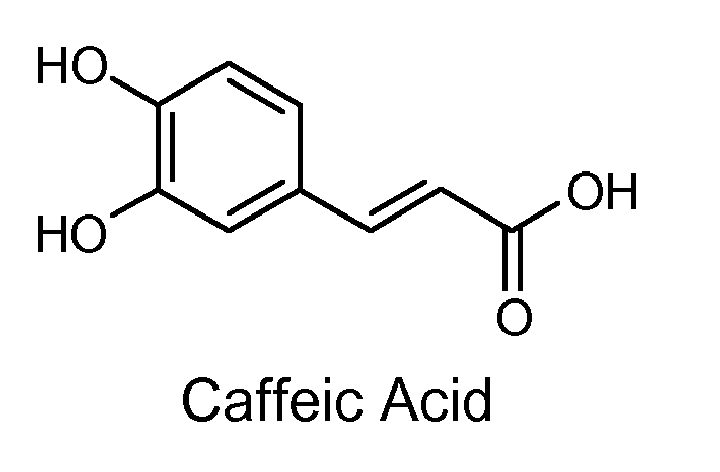  **Fig. S8:** Mass spectra of caffeic acid |
|   **Fig. S9:** Mass spectra of caffeic acid-*O*-hexoside |
|   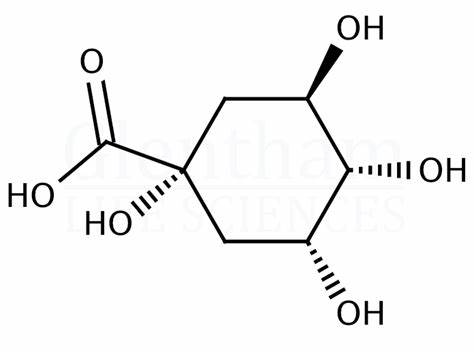  **Fig. S10 :** Mass spectra of quinic acid |
|   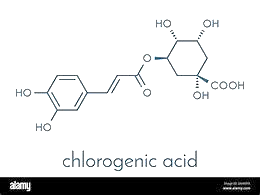  **Fig. S11 :** Mass spectra of chlorogenic acid |
|   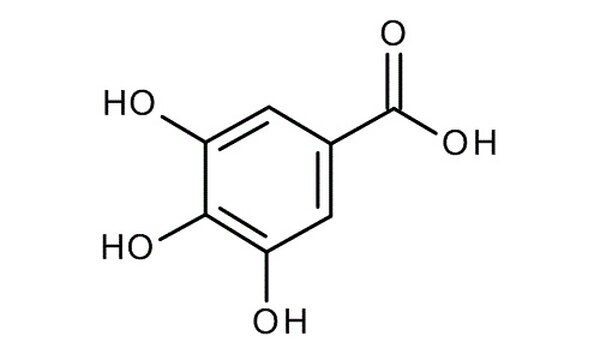  **Fig. S12a :** Mass spectra of gallic acid (-)mode    **Fig. S12b :** Mass spectra of gallic acid(+) mode |
|   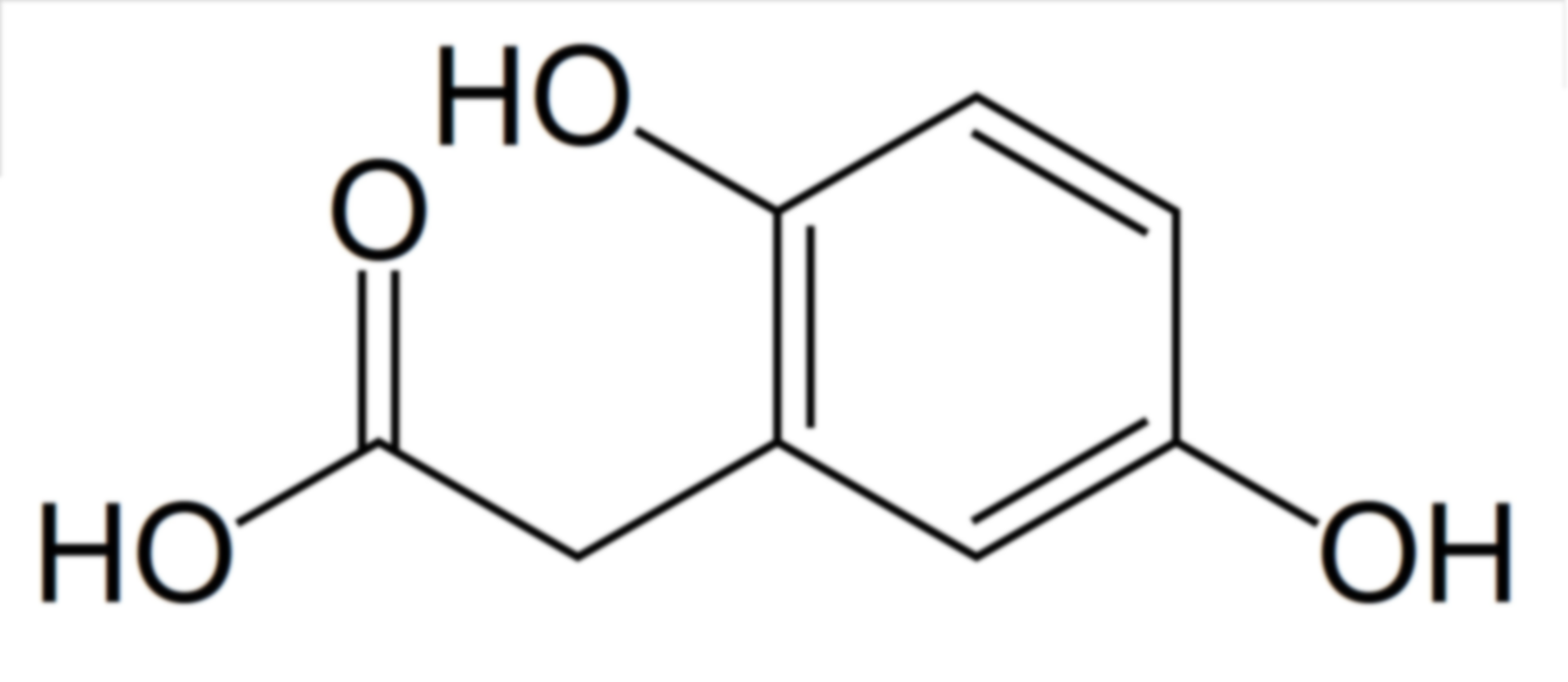  **Fig. S13 :** Mass spectra of homogentisic acid |
|   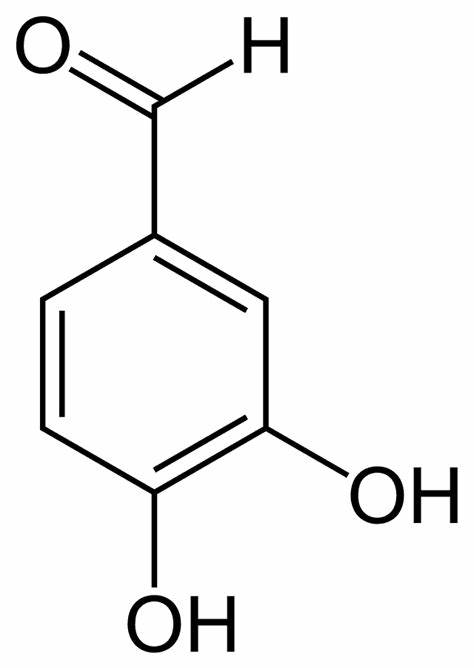  **Fig. S14 :** Mass spectra of protocatechuic aldehyde |
|   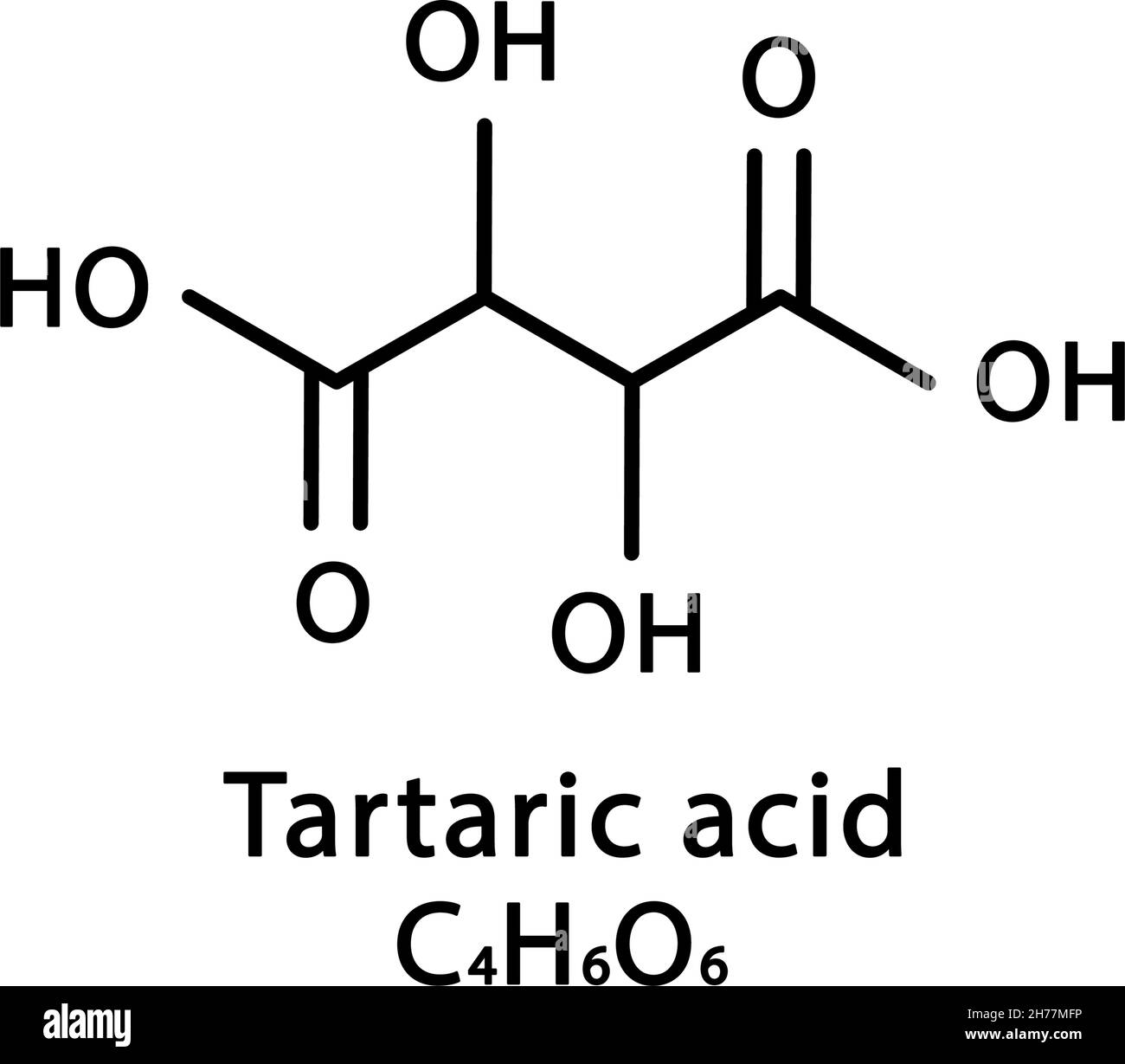  **Fig. S15 :** Mass spectra of tartaric acid |
|   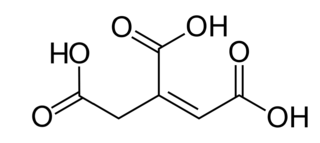  **Fig. S16 :** Mass spectra of aconitic acid |
|   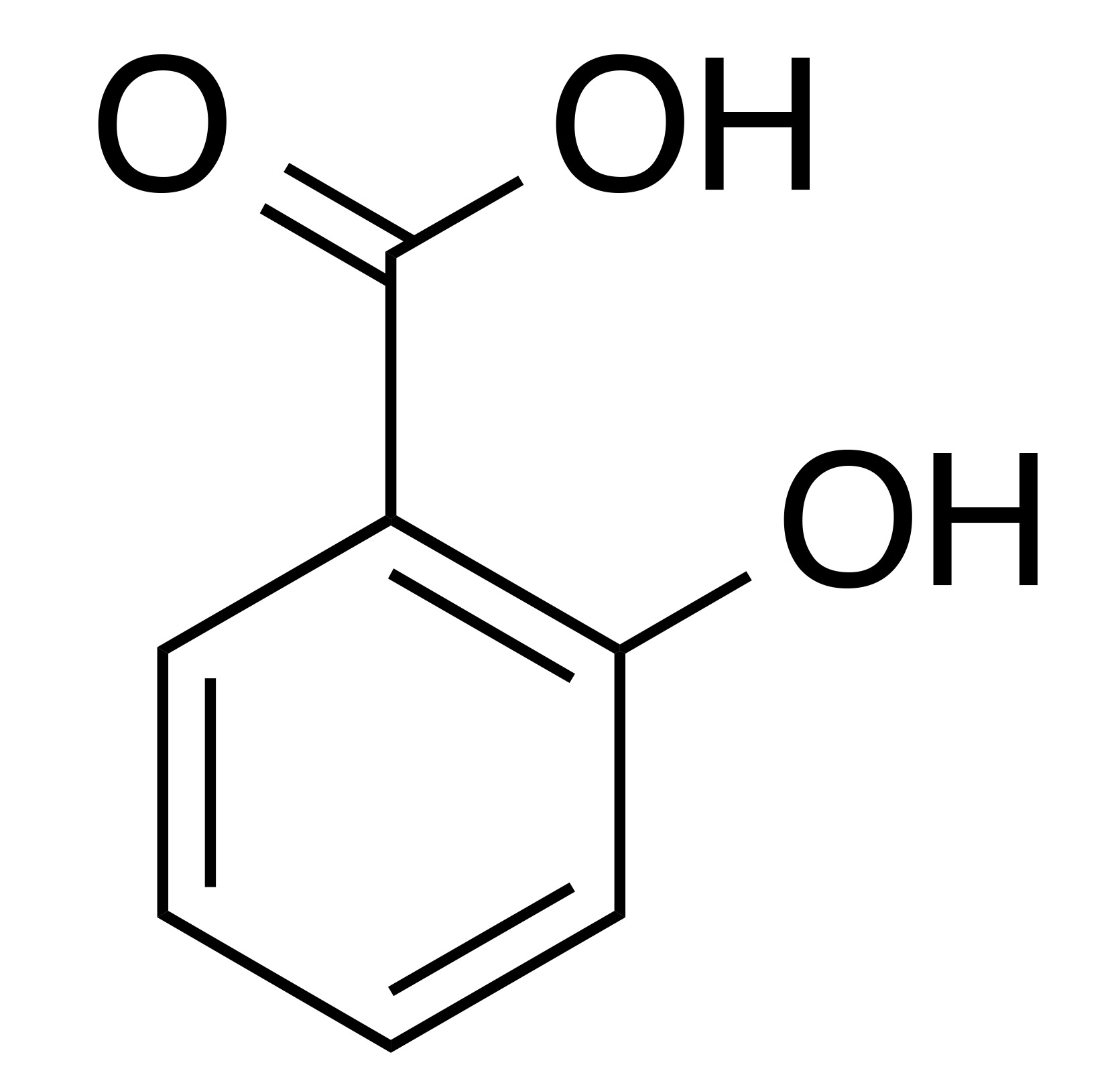  **Fig. S17 :** Mass spectra of salicylic acid |
|   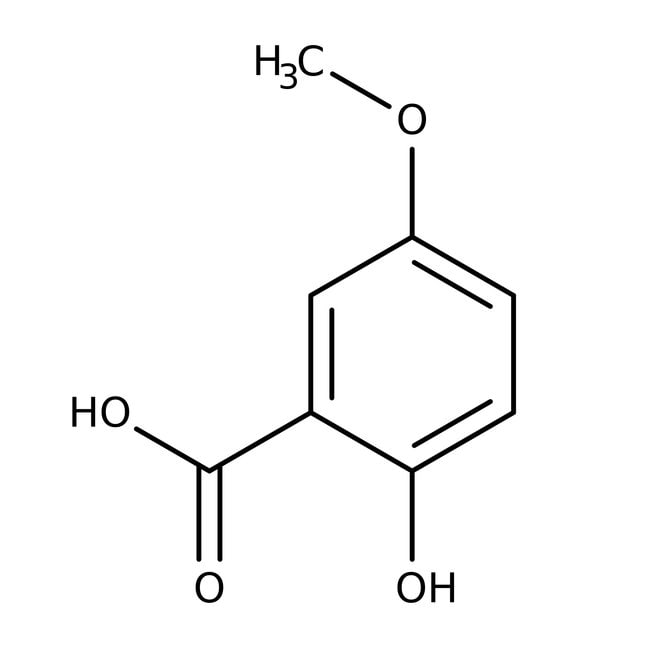  **Fig. S18 :** Mass spectra of 5-methoxysalicylic acid |
|   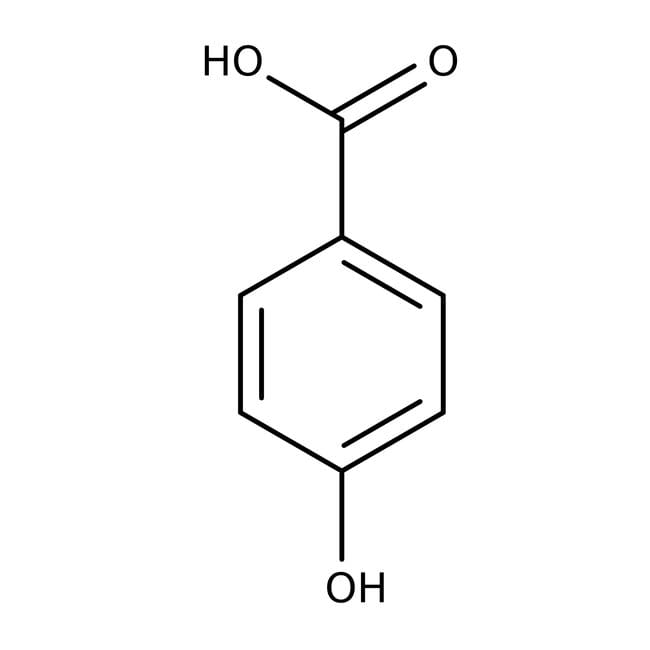  **Fig. S19 :** Mass spectra of 4-hydroxybenzoic acid |
|   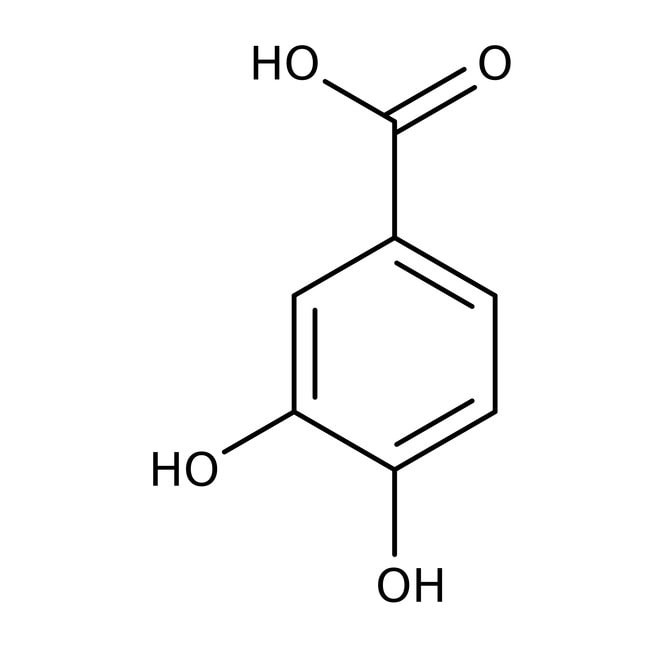  **Fig. S20:** Mass spectra of 3,4-dihydroxybenzoic acid |
|   **Fig. S21 :** Mass spectra of protocatechuic acid 4-*O*-hexoside |
|   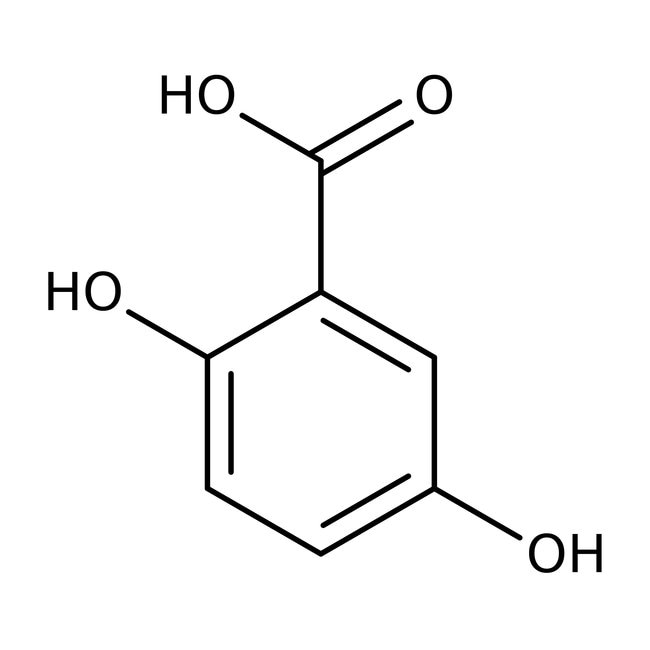  **Fig. S22 :** Mass spectra of 2,5-dihydroxybenzoic acid |
|     **Fig. S23a :** Mass spectra of vanillic acid (-)mode    **Fig. S23b :** Mass spectra of vanillic acid (+) mode |
|   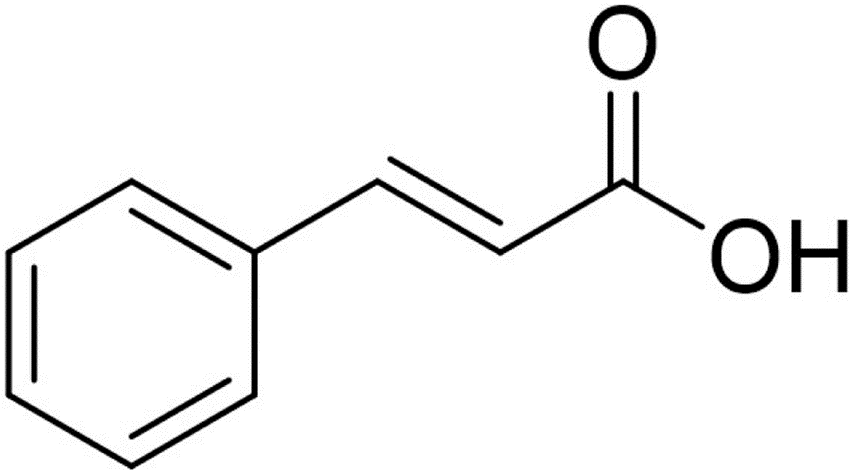  **Fig. S24 :** Mass spectra of cinnamic acid |
|   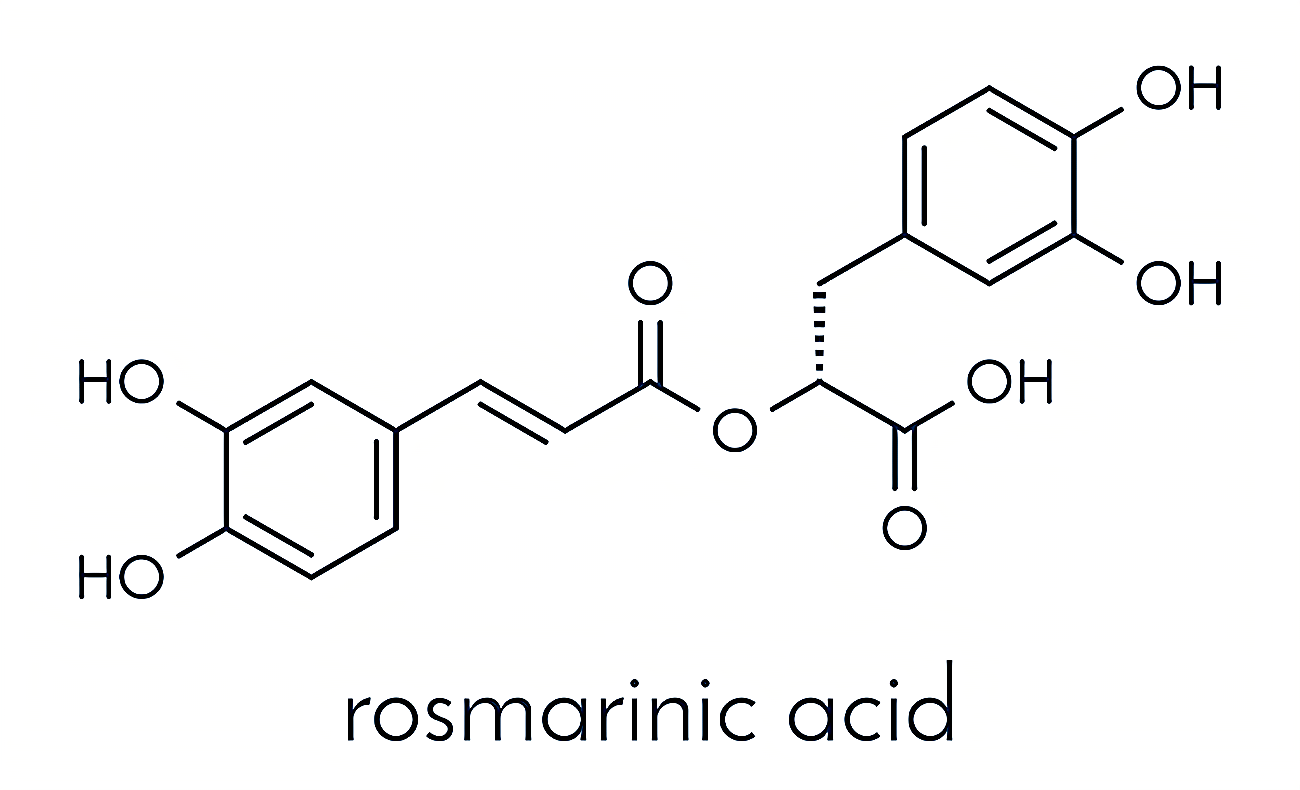  **Fig. S25 :** Mass spectra of rosmarinic acid |
|   **Fig. S26:** Mass spectra *of* 1-O-β-D-glucopyranosyl sinapate |
|   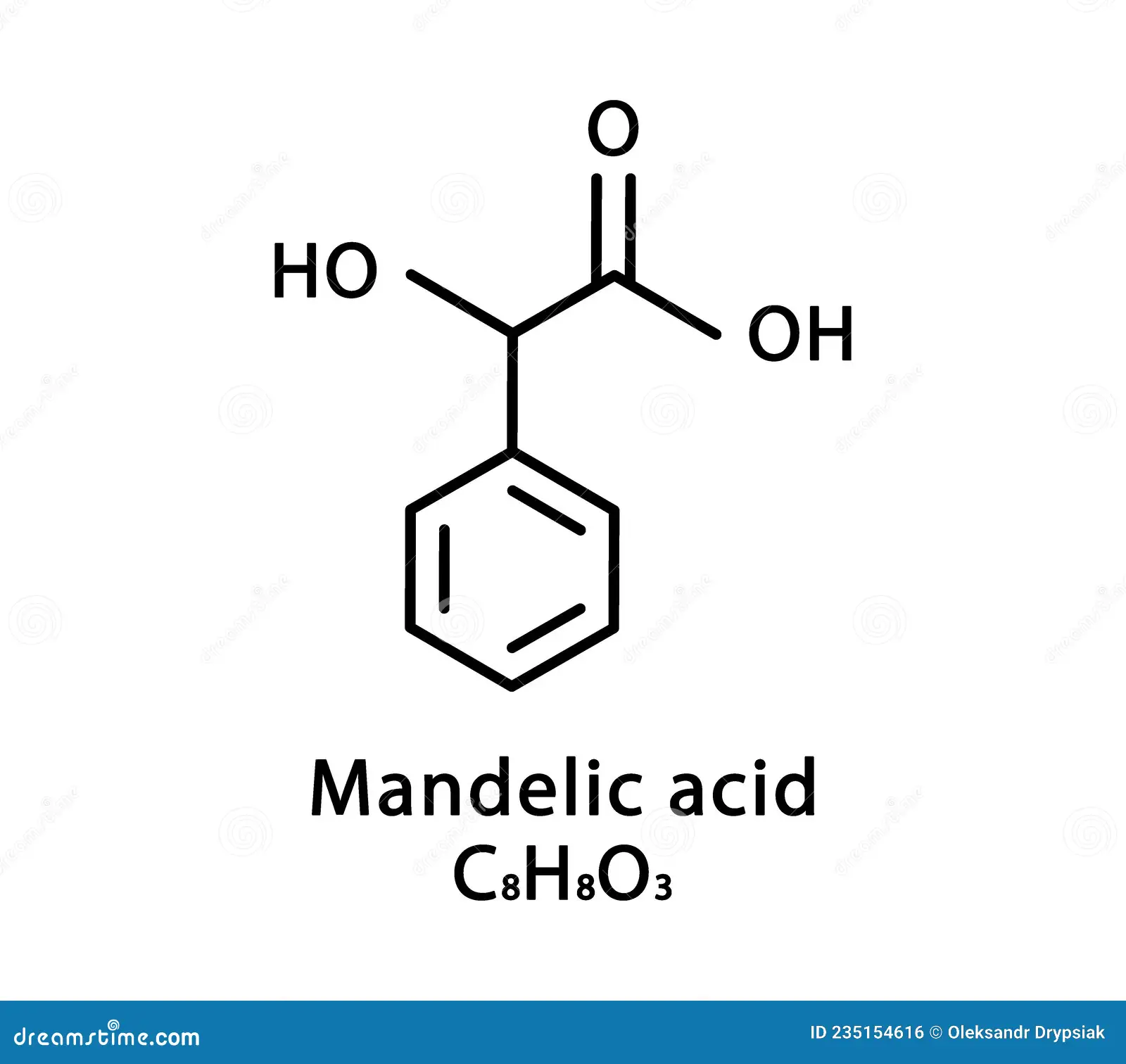  **Fig. S27 :** Mass spectra of mandelic acid |
|   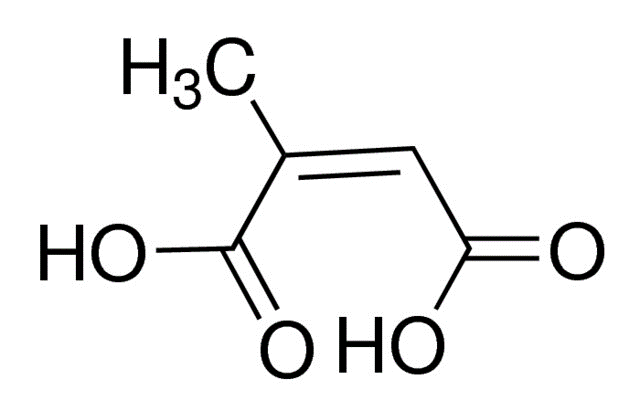  **Fig. S28 :** Mass spectra of citraconic acid |
|   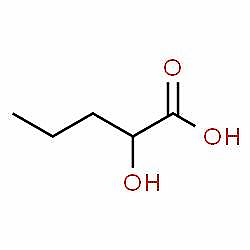  **Fig. S29:** Mass spectra of 2-hydroxyvaleric acid |
|   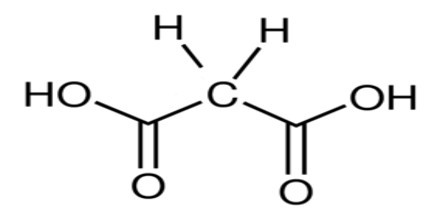  **Fig. S30:** Mass spectra of malonic acid |
|   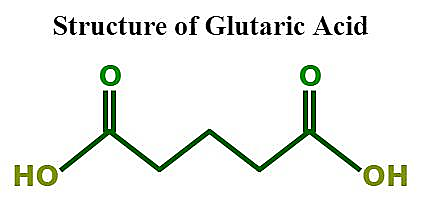  **Fig. S31 :** Mass spectra of glutaric acid |
|   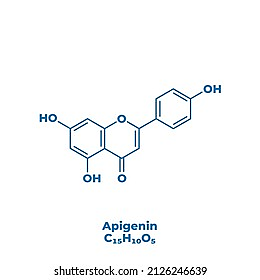  **Fig. S32a :** Mass spectra of apigenin (-)mode    **Fig. S32b :** Mass spectra of apigenin(+)mode |
|   **Fig. S33 :** Mass spectra of apigenin-7-*O*-hexose |
|   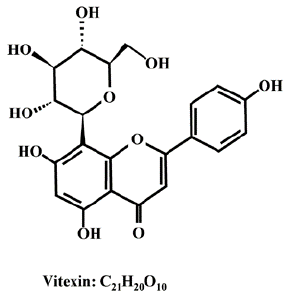  **Fig. S34:** Mass spectra of vitexin |
|   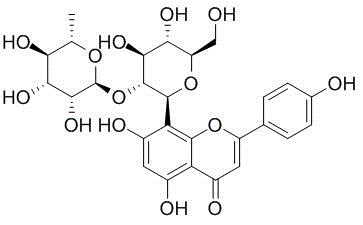  **Fig. S35 :** Mass spectra of vitexin-2''-*O*-rhamnoside |
|   **Fig. S36:** Mass spectra of 8-C-xylosyl-6-C-glucosyl apigenin |
|   **Fig. S37:** Mass spectra of Di-C, C-hexosyl-apigenin |
|   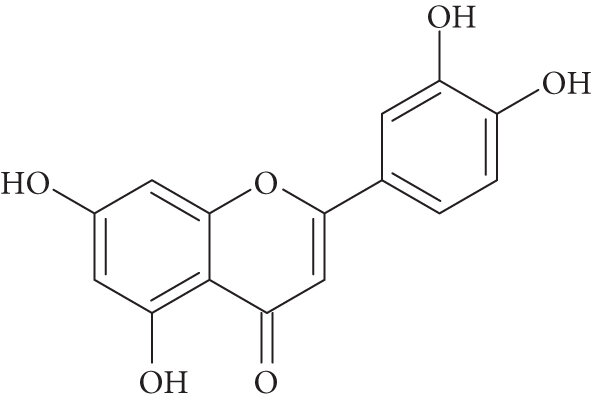  **Fig. S38a :** Mass spectra of luteolin (-)mode    **Fig. S38b :** Mass spectra of luteolin(+)mode |
|     **Fig. S39 :** Mass spectra of 6-C-glucosyl luteolin (isoorientin) |
|  |
|   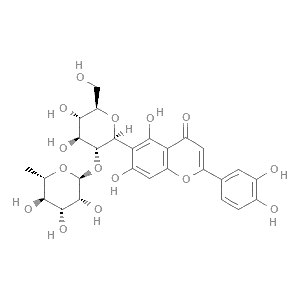  **Fig. S40a:** Mass spectra of isoorientin-2''-O-rhamnoside(-)mode    **Fig. S40b:** Mass spectra of isoorientin-2''-O-rhamnoside(+)mode |
|   **Fig. S41a:** Mass spectra of luteolin 7-*O*-hexoside (-)mode    **Fig. S41b:** Mass spectra of luteolin 7-*O*-hexoside (+)mode |
|   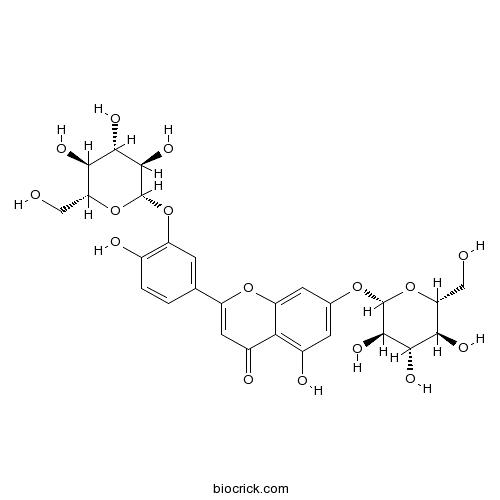  **Fig. S42:** Mass spectra of luteolin-3', 7-di-O-glucoside |
|   **Fig. S43:** Mass spectra of luteolin-6-C-pentosyl-8-C-pentoside |
|   **Fig. S44:** Mass spectra of luteolin-C-pentosyl-O-hexoside-methyl ether |
|   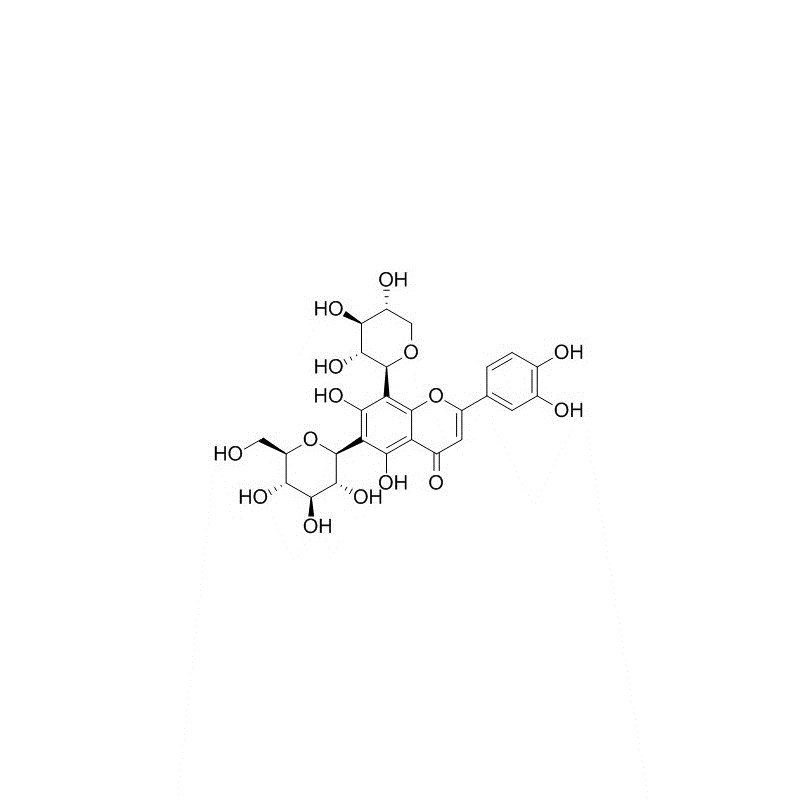  **Fig. S45 :** Mass spectra of carlinoside |
|   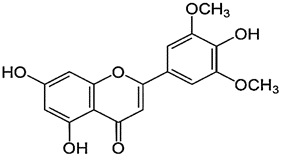  **Fig. S46a :** Mass spectra of tricin    **Fig. S46b :** Mass spectra of tricin |
|   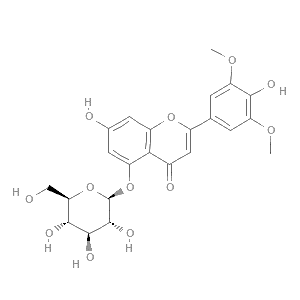  **Fig. S47a :** Mass spectra of tricin-5-O-glucoside (-)mode    **Fig. S47b :** Mass spectra of tricin-5-O-glucoside (+)mode |
|   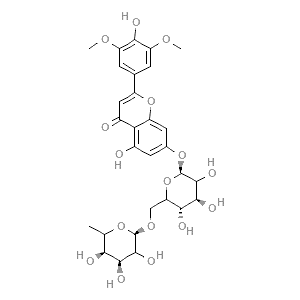  **Fig. S48:** Mass spectra of tricin-7-O-rutinoside |
|   **Fig. S49:** Mass spectra of tricin 4'-O-(erythro-β-guaiacylglyceryl) ether |
|   **Fig. S50:** Mass spectra of tricin 4'-O-(erythro-β-guaiacylglyceryl) ether7-O-glucoside |
|   **Fig. S51:** Mass spectra of Tricin 4'-O-(threo-β-guaiacylglyceryl) ether7-O-glucoside |
|   **Fig. S52 :** Mass spectra of tricin-*O*-deoxyhexosyl*- O*-hexosyl |
|   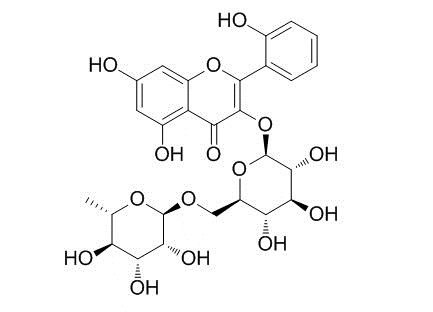  **Fig. S53:** Mass spectra of datiscin |
|   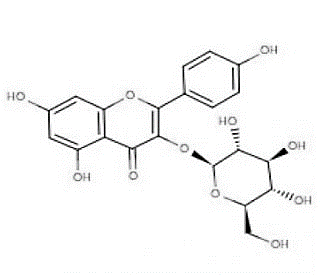  **Fig. S54:** Mass spectra of kaempferol-3-*O*-glucoside (astragalin) |
|  |
|   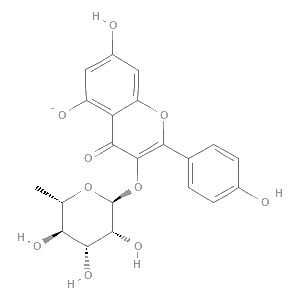  **Fig. S55:** Mass spectra of kaempferol-3-*O*-alpha-L-rhamnoside |
|   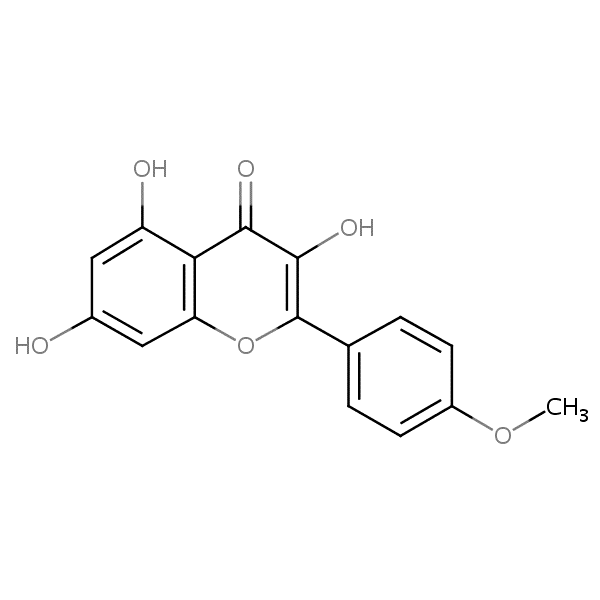  **Fig. S56:** Mass spectra of kaempferide |
|   **Fig. S57:** Mass spectra of isorhamnetin-3-O-hexoside |
|   **Fig. S58:** Mass spectra of syringetin-3-O-hexoside |
|   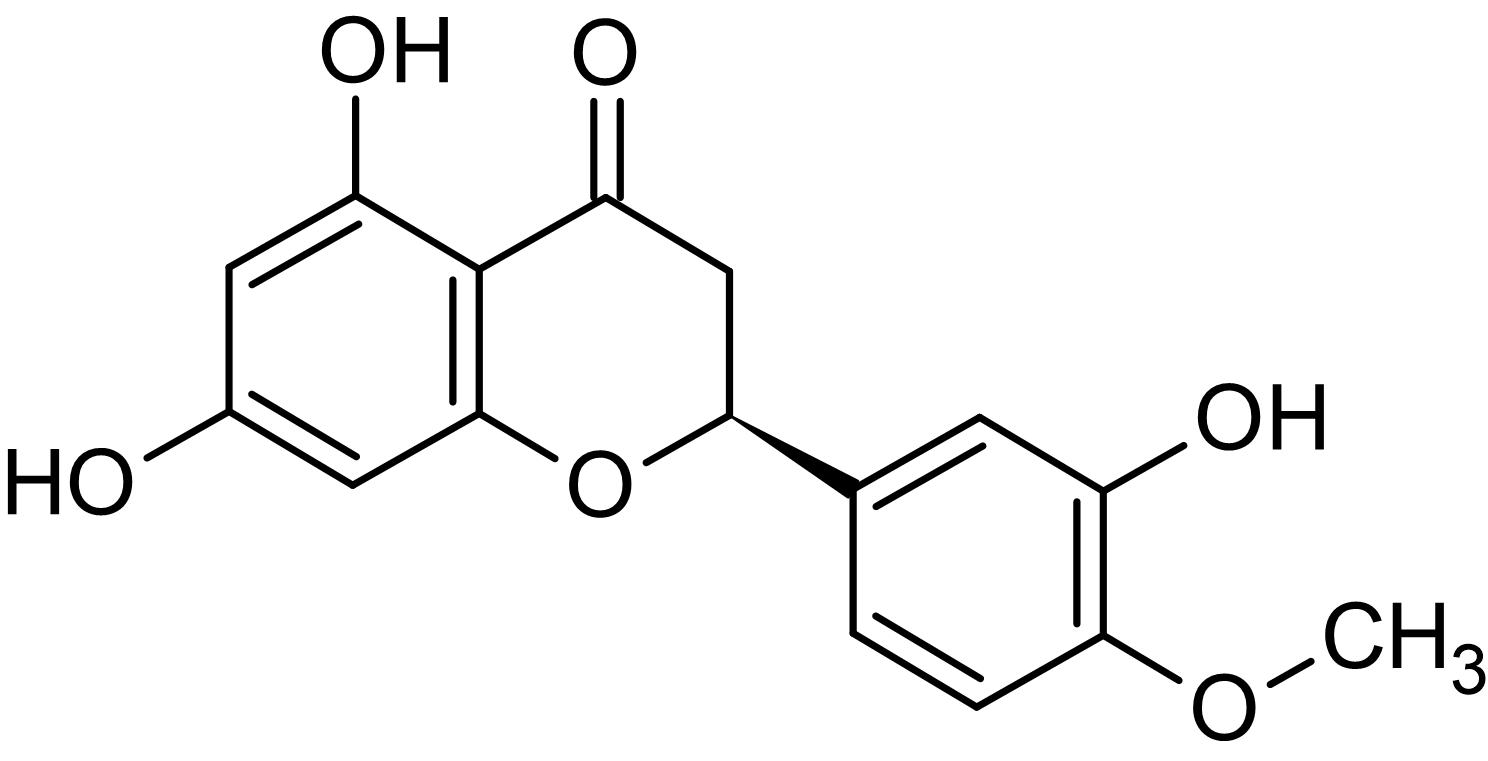  **Fig. S59:** Mass spectra of hesperetin |
|   **Fig. S60:** Mass spectra of okanin-4'-*O*- hexoside |
|   **Fig. S61:** Mass spectra of isookanin-7-hexoside |
|   **Fig. S62a:** Mass spectra of daidzein (-)mode    **Fig. S62b:** Mass spectra of daidzein (+)mode |
|   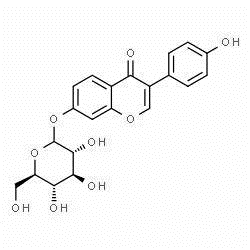  **Fig. S63:** Mass spectra of daidzein-7-*O*-glucoside |
|   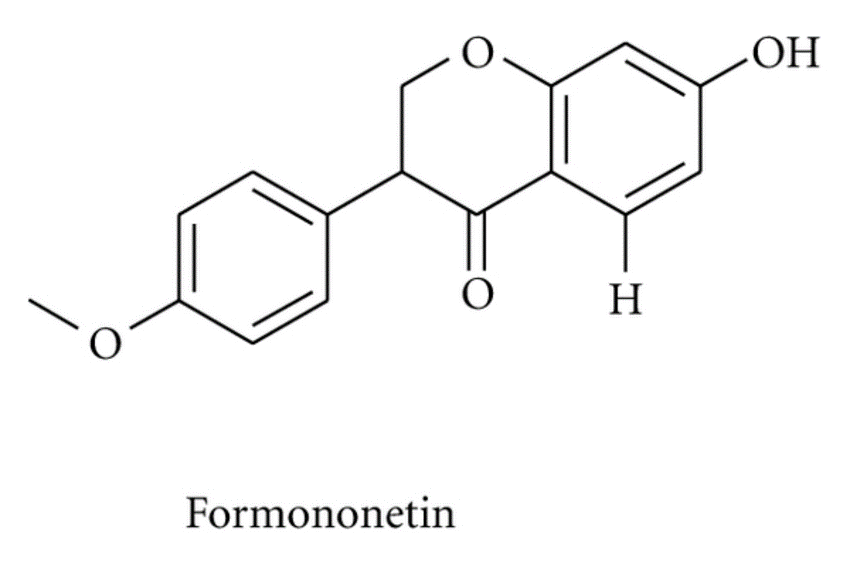  **Fig. S64:** Mass spectra of formononetin |
|   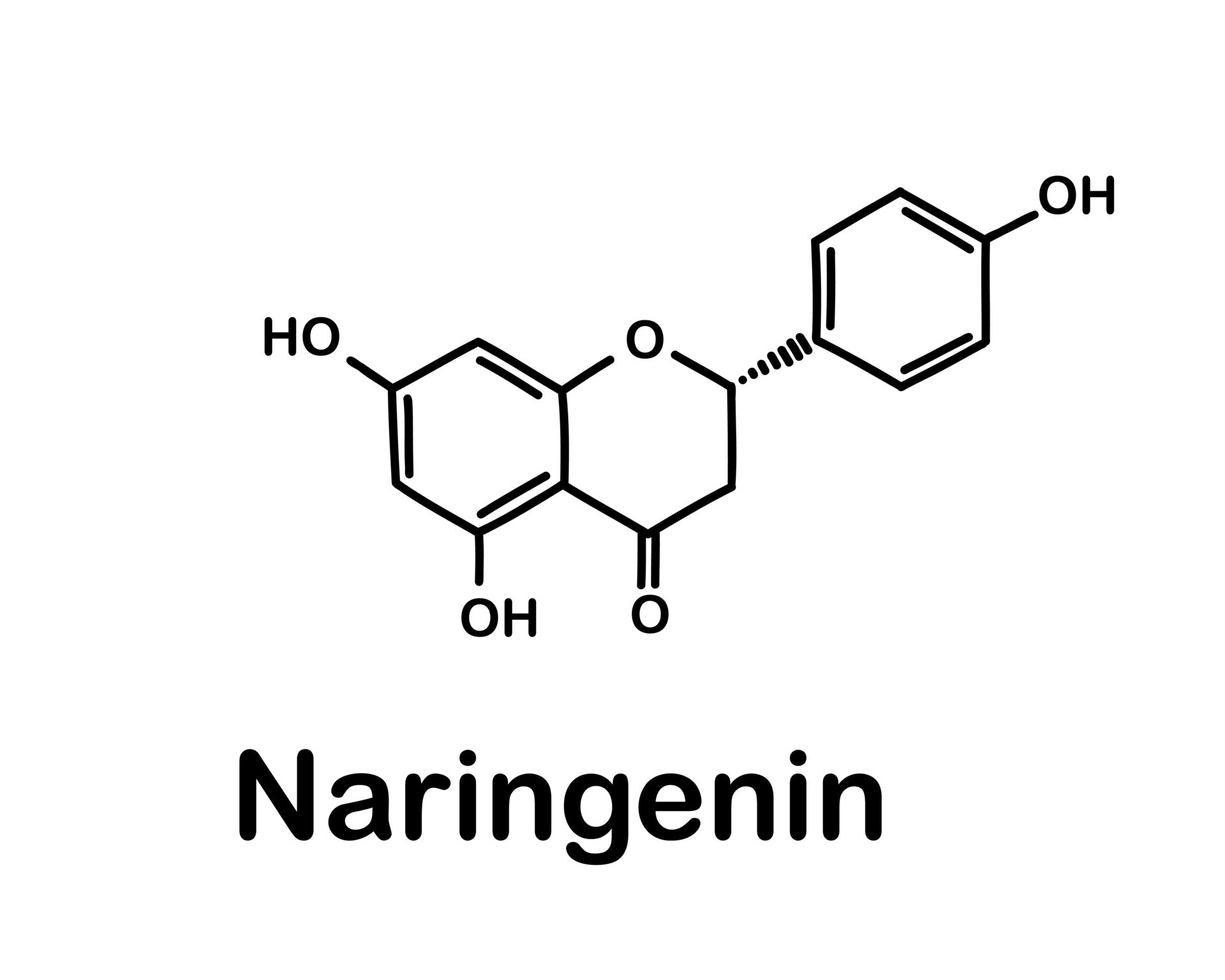  **Fig. S65 :** Mass spectra of naringenin |
|   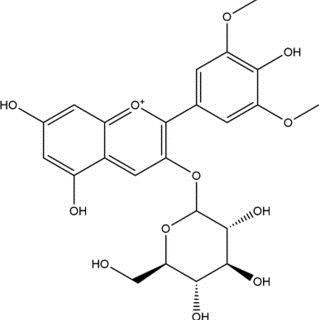  **Fig. S66 :** Mass spectra of malvidin-3-*O*-glucoside |
|   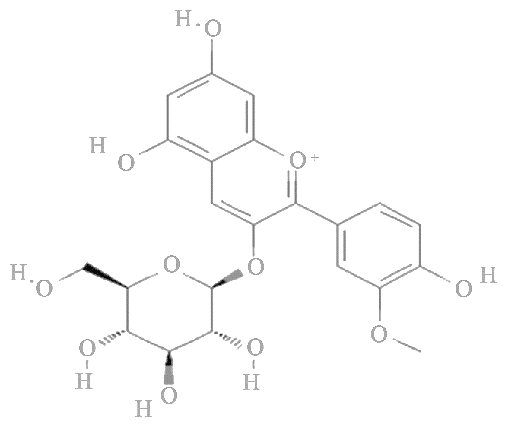  **Fig. S67:** Mass spectra of peonidine-3-*O*-glucoside |
|   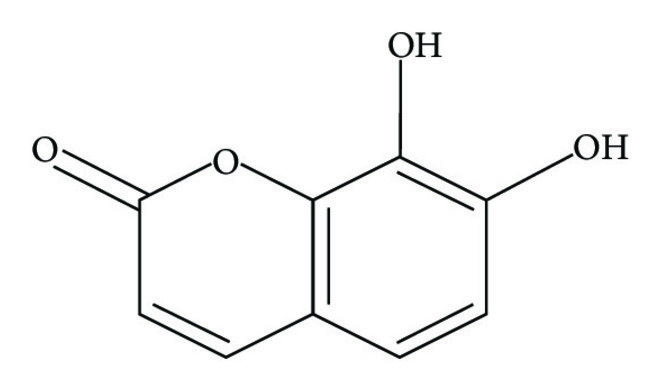  **Fig. S68:** Mass spectra of daphnetin |
|   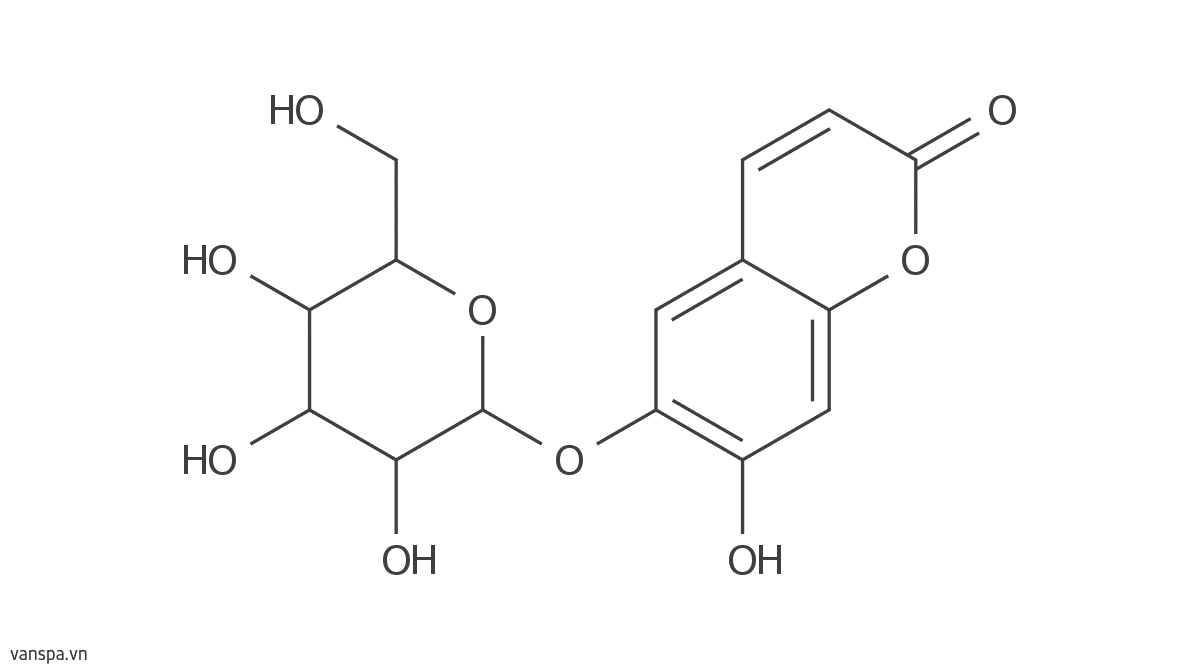  **Fig. S69:** Mass spectra of esculin |
|   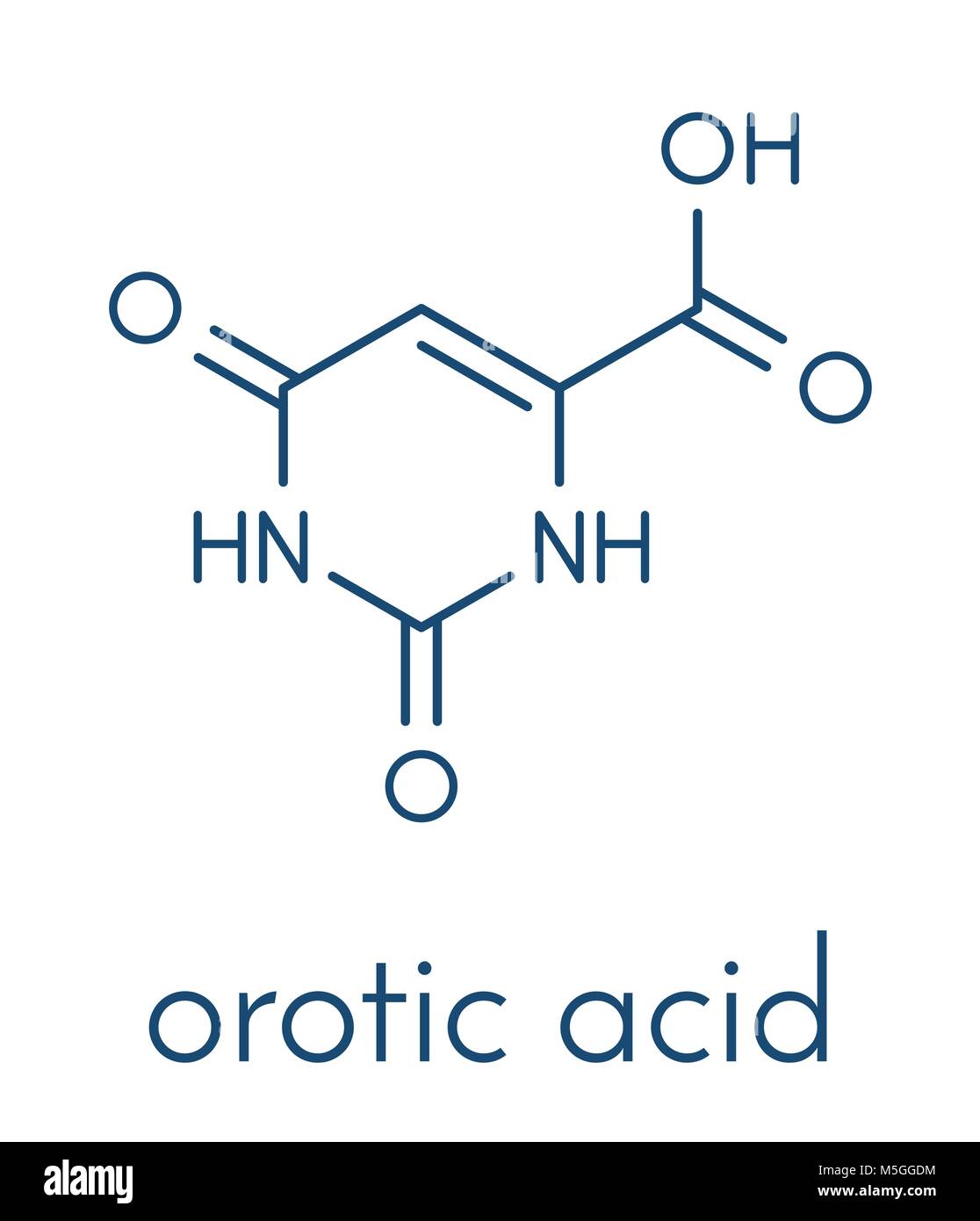  **Fig. S70:** Mass spectra of orotic acid |
|   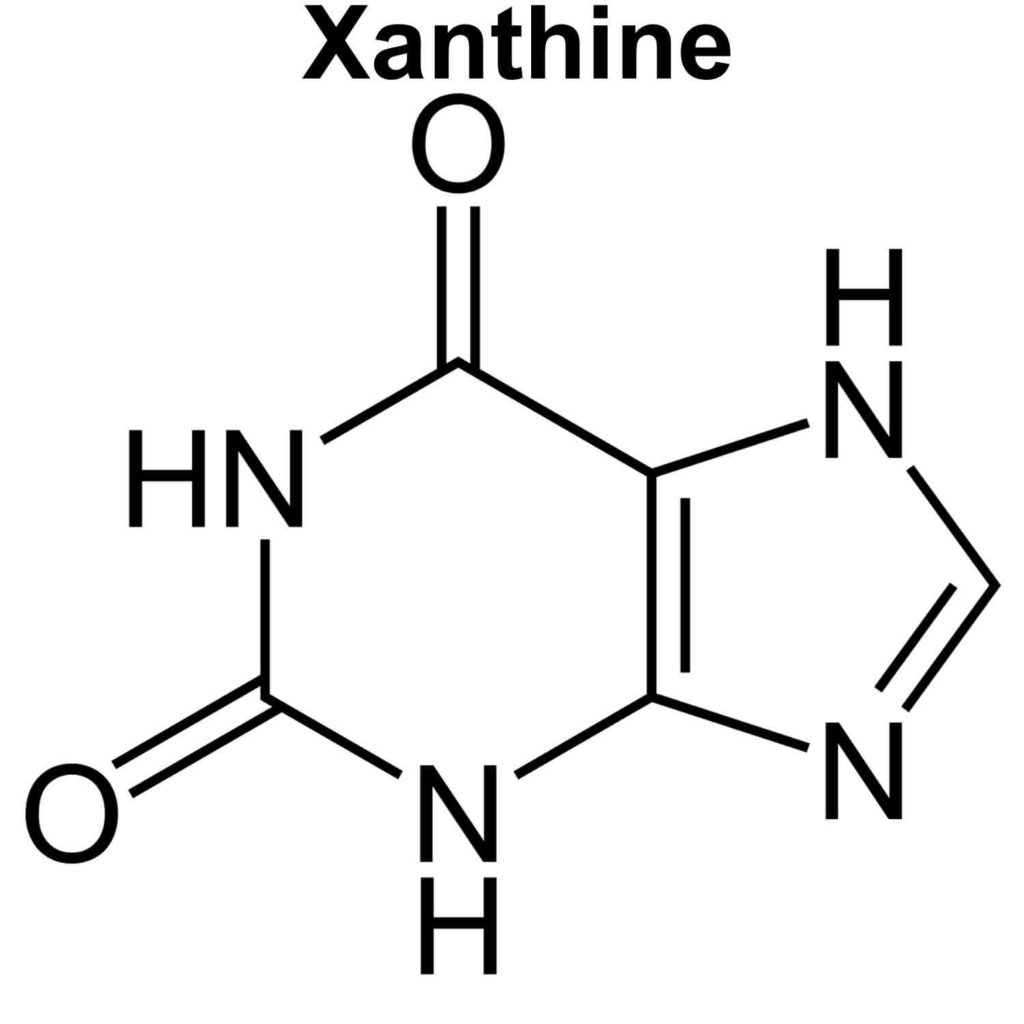  **Fig. S71:** Mass spectra of xanthine |
|   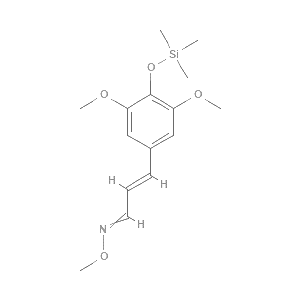  **Fig. S72:** Mass spectra of sinapyl aldehyde |
|   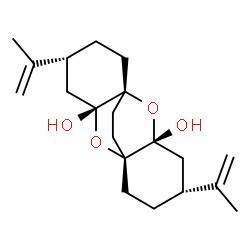  **Fig. S73 :** Mass spectra of cymbodiacetal |
|   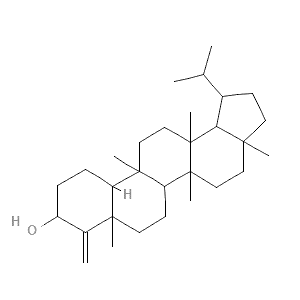  **Fig. S74:** Mass spectra of cymbopogonol |
|   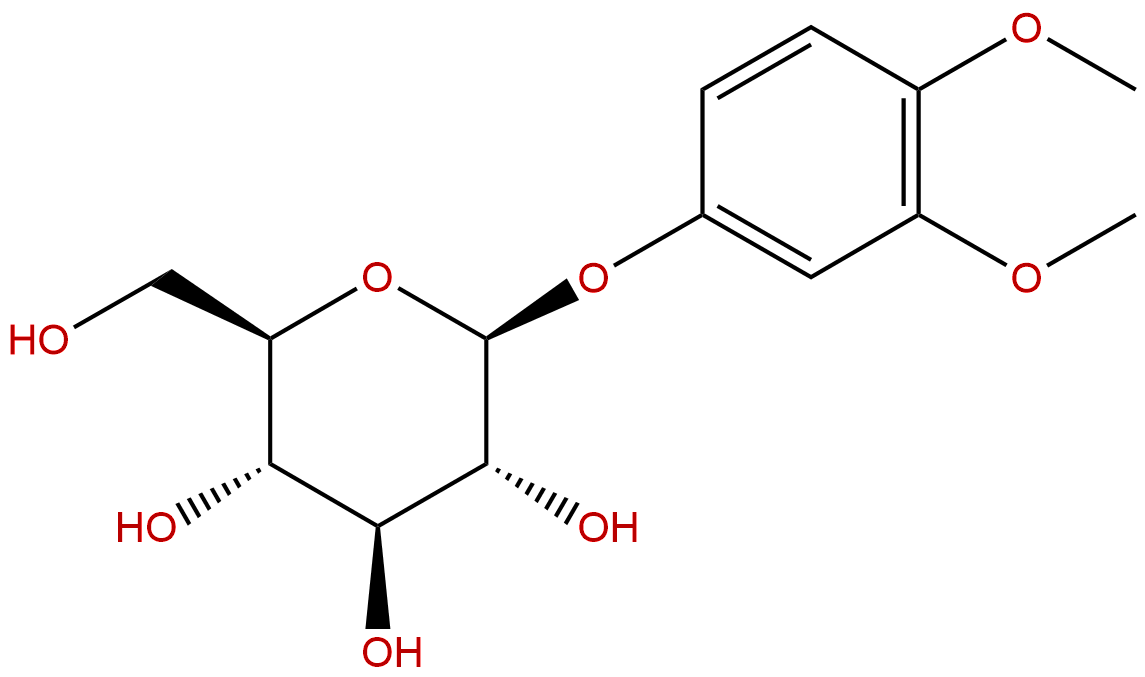  **Fig. S75 :** Mass spectra of 3,4-Dimethoxyphenyl β-D glucopyranoside |
| 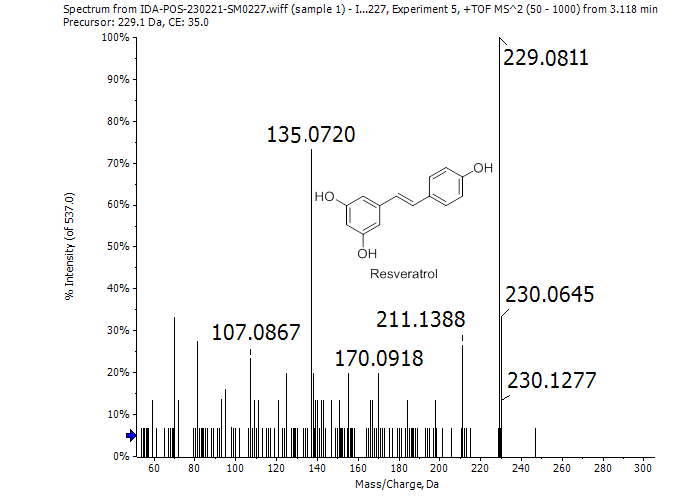  **Fig. S76 :** Mass spectra of resveratrol |

**Supplementary 4:**

**Table S1**: Mass data of compounds tentatively identified in *C. proximus* extract using LC /MS in negative and positive ion modes.

| No. | R_t_ min. | Peak area | MassError (ppm) | Adduct | [M-H]^-^/[M+H]^+^/ [M]^+^  *m/z* | MS/MS major fragments | Molecular Formula | Tentatively identified metabolites | References* |
| --- | --- | --- | --- | --- | --- | --- | --- | --- | --- |
| 1- Flavonoids  1.1. Flavones | | | | | | | | | |
| 1.1.1. Apigenin derivatives | | | | | | | | | |
| 1 | 4.23 | 132711.984 | −1.2 | [M-H]^-^ | 563.1409 | 473.11195, 433.1017, 383.0805,353.0691 | C_26_H_28_O_14_ | 8-C-xylosyl-6-C-glucosyl apigenin | [**^5^**](#_ENREF_5) |
|  | 5.70 | 2264209.5 | -0.9 | [M+H] ^+^ | 565.1567 | 566.1511, 565.1515, 547.1423, 529.1315,445.1058, 379.0790, 325.0689 |  |  | [**^6^**](#_ENREF_6) |
| 2 | 5.79 | 1954639.88 | 5.8 | [M+H] ^+^ | 595.1653 | 457.1137, 427.1046, 379.0836, 325.0724 | C_27_ H_30_ O_15_ | Di-C, C-hexosyl-apigenin | [^6^](#_ENREF_6) |
| 3 | 5.81 | 828816.813 | 0.5 | [M-H]- | 577.1545 | 457.1235, 413.0880, 353.0507, 341.0566, 311.0571, 293.0457, 283.0645 | C_27_H_30_O_14_ | Vitexin 2''-*O*-rhamnoside | [^7^](#_ENREF_7) |
|  | 7.99 | 100015.93 | -0.1 | [M+H] ^+^ | 579.1785 | 579.35376, 519.2340, 433.108, 415.1067, 343.0678, 351.9754, 333.0667, 313.0618 |  |  | [^8^](#_ENREF_8) |
| 4 | 6.01 | 98091.7734 | -0.1 | [M-H]- | 431.1002 | 341.0727, 311.0575, 283.0610 | C_21_H_20_O_10_ | Apigenin 8-C-glucoside (Vitexin) | [^7^](#_ENREF_7) |
|  | 6.62 | 77194.8281 | 1.1 | [M+H] ^+^ | 433.1138 | 433.20831, 415.10141, 373.15158, 367.09344, 343.07761, 313.06604 |  |  | [^9^](#_ENREF_9) |
| 5 | 7.70 | 397713.84 | 1.9 | [M-H]- | 269.1767 | 251.1661, 193.1239, 151.1105 | C_15_H_10_O_5_ | Apigenin | [^10^](#_ENREF_10) |
|  | 3.56 | 109913.086 | 6.3 | [M+H] ^+^ | 271.0988 | 253.0640, 189.1675, 153.0954 |  |  | [^5^](#_ENREF_5) |
| 1.1.2. Luteolin derivatives | | | | | | | | | |
| 6 | 5.05 | 176672.656 | -0.3 | [M-H]- | 609.1438 | 563.1334, 447.0916, 429.1023, 357.0694, 327.0494, 298.0764 | C_27_H_30_O_16_ | Luteolin 3', 7-di-*O*-glucoside | [^11^](#_ENREF_11) |
|  | 1.44 | 20684.3867 | 0.9 | [M+H]+ | 611.1749 | 610.1776, 609.1438, 447.0913, 327.0494 |  |  | [^12^](#_ENREF_12) |
| 7 | 5.42 | 47301.38 | 1.2 | [M-H]- | 447.1071 | 429.0852, 357.0621, 327.0514, 285.0385 | C_21_H_20_O_11_ | Luteolin-6-C-glucoside (Isoorientin) | [^13^](#_ENREF_13) |
|  | 5.88 | 134102.172 | 0.9 | [M+H]^+^ | 449.1079 | 449.2137, 389.1449, 359.06708, 329.07269, 311.05017 |  |  | [^14^](#_ENREF_14) |
| 8 | 6.28 | 49370.0469 | 4.3 | [M-H]^-^ | 549.1224 | 531.1327, 489.0986, 459.0893, 441.0843, 399.0692, 369.0680 | C_25_H_26_O_14_ | Luteolin-6-C-pentosyl-8-C-pentoside | [^15^](#_ENREF_15) |
| 9 | 6.99 | 26715.12 | 4.1 | [M-H]^-^ | 579.1692 | 561.0975, 519.1299, 489.1029, 459.0943, 417.1059, 399.0770, 369.0519 | C_26_H_28_O_15_ | luteolin-6-*C*-glucoside-8-*C*-arabinoside (carlinoside) | [^15^](#_ENREF_15) |
| 10 | 7.31 | 158652.047 | 1.3 | [M-H]^-^ | 447.0923 | 285.0412, 242.9575, 174.9560 | C_21_H_20_O_11_ | Luteolin 7-*O*-hexoside  (Cynaroside) | [^16^](#_ENREF_16) |
|  | 6.97 | 18450.4531 | -0.6 | [M+H]^+^ | 449.1025 | 287.0537, 286.1208 |  |  | [^5^](#_ENREF_5) |
| 11 | 9.70 | 20786.669 | 8.8 | [M-H]^-^ | 593.1293 | 447.0827, 429.0791, 357.0614, 327.0520, 285.0412 | C_27_H_30_O_15_ | Isoorientin-2''-O-rhamnoside | [^1^](#_ENREF_1) |
|  | 5.73 | 77944.6953 | -6.7 | [M+H]^+^ | 595.1686 | 595.1686, 449.1081, 431.0989, 395.0806, 353.0635, 329.0662, 299.0558, 287.0549 |  |  | [^5^](#_ENREF_5) |
| 12 | 20.26 | 41133.87 | -1 | [M-H]^-^ | 285.0428 | 257.0517, 241.0487 | C_15_H_10_O_6_ | Luteolin | [^15^](#_ENREF_15) |
| 13 | 27.07 | 91464.8438 | 3.1 | [M-H]^-^ | 593.1467 | 579, 575, 533, 503, 473.0987, 327.0498,  285.0237 | C_27_H_30_ O_15_ | Luteolin-C-pentosyl-O-hexoside-methyl ether | [^15^](#_ENREF_15) |
| 1.1.3. Tricin derivatives | | | | | | | | | |
| 14 | 7.32 | 157374.328 | 8.5 | [M-H]^-^ | 491.1241 | 445.1509, 329.1400, 160.0503 | C_23_H_24_O_12_ | Tricin-5-O-hexoside | [^1^](#_ENREF_1) |
|  | 7.15 | 699381.688 | 10 | [M+H]^+^ | 493.1281 | 331.0785, 316.0563, 315.0529, 270.0524 |  |  | [^6^](#_ENREF_6) |
| 15 | 7.42 | 190387.797 | 7.8 | [M-H]^-^ | 637.1837 | 491.1256, 329.0647, 270.0195 | C_29_H_34_O_16_ | Tricin-7-O-rutinoside | [^1^](#_ENREF_1) |
| 16 | 7.58 | 170785.984 | −3.6 | [M+H]^+^ | 639.1946 | 493.1095, 331.0832, 315.0425, 270.0567 | C_29_H_34_O_16_ | Tricin-O-deoxyhexosyl- O-hexosyl | [^6^](#_ENREF_6) |
| 17 | 8.11623 | 73133.1875 | 0.2 | [M-H]^-^ | 687.1930 | 525.1474, 491.1173m 329.0576, 314.0362, 195.0706, 165.0449 | C_33_H_36_O_16_ | Tricin 4'-O-(erythro-b-guaiacylglyceryl) ether7-O-glucoside | [^1^](#_ENREF_1) |
| 18 | 8.406317 | 263089.469 | 0.25 | [M-H]^-^ | 687.1931 | 525.1508, 491.1050, 329.0682, 314.0541, 299.0192, 195.0670, 165.0561 | C_33_H_36_O_16_ | Tricin 4'-O-(threo-b-guaiacylglyceryl) ether7-O-glucoside | [^1^](#_ENREF_1) |
| 19 | 10.33938 | 682294.563 | 6.6 | [M-H]^-^ | 525.1357 | 477.1282, 329.0654, 315.0407, 299.0263, 195.0648, 165.0541 | C_27_H_26_O_11_ | Tricin 4'-O-(erythro-b-guaiacylglyceryl) ether | [^1^](#_ENREF_1) |
| 20 | 10.35188 | 1313417.38 | 6.2 | [M-H]^-^ | 525.1359 | 477.1282, 329.0654, 315.0407, 299.0263, 195.0648, 165.0541 | C_27_H_26_O_11_ | Tricin 4'-O-(threo-b-guaiacylglyceryl) ether | [^1^](#_ENREF_1) |
| 21 | 10.36 | 7863532.5 | 5.8 | [M-H]- | 329.0653 | 314.0413, 299.0195, 271.0247, 270.1971 | C_17_H_14_O_7_ | Tricin | [^1^](#_ENREF_1) |
|  | 11.10 | 2766313.5 | 0.8 | [M+H]+ | 331.0798 | 315.0500, 270.0521, 258.0522, 153.0183 |  |  | [^6^](#_ENREF_6) |
| 1.1.4. Other flavone derivatives | | | | | | | | | |
| 22 | 5.149 | 250381.656 | 2.4 | [M+H]+ | 609.1762 | 609.18512, 594.15125, 463.05225, 301.13379, 286.08228 | C_28_H_32_O_15_ | Diosmin | [^17^](#_ENREF_17) |
| 23 | 6.505 | 176344.688 | 10 | [M+H]+ | 447.1618 | 447.18066, 429.16122, , 355.13535, 271.13306,225.18701 | C_21_H_18_O_11_ | Baicalein 7-*O*-glucuronide | [^18^](#_ENREF_18) |
| 1.2. Flavonols | | | | | | | | | |
| 24 | 1.55 | 179604.547 | -2.4 | [M+H]+ | 319.102 | 319.09634, 301.13776, 245.06659, 153.06377,  137.04264 | C_15_H_10_O_8_ | Myricetin | [^19^](#_ENREF_19) |
| 25 | 2.00 | 303823.688 | 0.8 | [M+H]+ | 303.1072 | 303.10654, 285.09738, 153.05743, 148.95508, 137.03788 | C_15_H_10_O_7_ | Quercetin | [^19^](#_ENREF_19) |
| 26 | 4.17 | 66099.9375 | -2.5 | [M+H]+ | 419.1587 | 419.1577, 287.1123, 286.8761, 269.93489, 241.91847, 153.92346 | C_20_H_18_O_10_ | Kaempferol 3-*O*-pentoside | [^20^](#_ENREF_20) |
| 27 | 5.26 | 34185.50 | 0.4 | [M-H]- | 477.101 | 315.0719 | C_22_H_22_O_12_ | Isorhamnetin-3-O-hexoside | [^2^](#_ENREF_2)^,^[^21^](#_ENREF_21) |
| 28 | 6.71 | 198371.313 | -3.6 | [M-H]- | 507.1682 | 345.1162 | C_23_H_24_O_13_ | Syringetin-3-O-hexoside | [^22^](#_ENREF_22) |
| 29 | 8.51 | 346394.81 | 3.5 | [M-H]- | 593.1465 | 447.0982, 285.0326 | C_27_H_30_O_15_ | Kaempferol-3-*O*-rutinoside | [^2^](#_ENREF_2)^,^[^21^](#_ENREF_21) |
| 30 | 9.833 | 86383.1484 | -7.2 | [M+H]^+^ | 317.0617 | 317.0361, 303.0353, 299.1999, 285.0447, 275.0544 | C_16_H_12_O_7_ | Isorhamnetin | [^23^](#_ENREF_23) |
| 31 | 10.41 | 371473.31 | -0.2 | [M-H]^-^ | 299.0987 | 284.0337, 283.0658, 256.0347 | C_16_H_12_O_6_ | Kaempferide  (3, 5, 7-trihydroxy-4'-methoxyflavone) | [^24^](#_ENREF_24) |
|  | 11.20 | 25831.8105 | 0.1 | [M+H]^+^ | 301.0698 | 301.2114, 286.04956, 269.1111, 259.05521,133.09967, 159.11581 |  |  | [^9^](#_ENREF_9) |
| 32 | 27.00 | 2473243 | -0.6 | [M-H]- | 593.1497 | 447.0874, 285.0381 | C_27_H_30_O_15_ | Datiscin | [^25^](#_ENREF_25) |
| 1.3. Flavanones | | | | | | | | | |
| 33 | 3.99 | 171521.46 | -10 | [M-H]^-^ | 271.1553 | 253.1368, 151.1158***,*** 125.1017, 109.0282 | C_15_H_12_O_5_ | Naringenin | [^26^](#_ENREF_26) |
|  | 7.302 | 951111.813 | 0.8 | [M+H]^+^ | 273.1703 | 203.1795, 147.1175 |  |  | [^27^](#_ENREF_27) |
| 34 | 5.013 | 137626.781 | -5.4 | [M-H]^-^ | 449.1043 | 287.0674, 268.8969, 180.0771 | C_21_H_22_O_11_ | Okanin-4'-O- hexoside  (Marein) | [^28^](#_ENREF_28) |
| 35 | 5.051 | 521119.125 | -1.7 | [M+H]^+^ | 289.1634 | 289.1633, 271.19675, 179.07623, 163.04478, 153.09888 | C_15_H_12_O_6_ | 3', 4', 5, 7-tetrahydroxyflavanone  (Eriodictyol) | [^12^](#_ENREF_12) |
| 36 | 6.698 | 46275.5 | -4.7 | [M-H]^-^ | 449.104 | 287.0538, 151.0053 | C_21_H_22_O_11_ | Isookanin-7- hexoside | [^29^](#_ENREF_29) |
| 37 | 11.35 | 925120.313 | 0.3 | [M+H]^+^ | 437.1198 | 437.28561, 275.07791, 153.08881,145.0274, 139.1161 | C_21_H_24_O_10_ | Phlorizin | [^30^](#_ENREF_30) |
| 38 | 13.94 | 77457.01 | 2 | [M-H]^-^ | 301.1440 | 286.0592, 151.0033 | C_16_H_14_O_6_ | Hesperetin | [^31^](#_ENREF_31) |
| 1.4. Isoflavones | | | | | | | | | |
| 39 | 4.570 | 138329.516 | 0 | [M+H]^+^ | 271.1146 | 271.11646, 243.16539, 215.11896, 153.06526, 145.10316 | C_15_H_10_O_5_ | Genistein | [^32^](#_ENREF_32) |
| 40 | 6.02 | 302356.06 | 4.4 | [M-H]^-^ | 267.1586 | 252.0608, 160.0160 | C_16_H_12_O_4_ | Formononetin | [^26^](#_ENREF_26) |
| 41 | 10.21 | 323155.06 | 4.6 | [M-H]^-^ | 253.2162 | 235.1698, 135.0447, 132.0865, 91.0195 | C_15_H_10_O_4_ | Daidzein | [^4^](#_ENREF_4) |
|  | 15.76 | 201706.188 | 10 | [M+H]^+^ | 255.1565 | 199.0975, 137.1340, 91.0548 |  |  | [^4^](#_ENREF_4) |
| 42 | 12.29 | 721082.81 | 5.1 | [M-H]^-^ | 415.1579 | 253.0728 | C_21_H_20_O_9_ | Daidzein-7-*O*-glucoside  (Daidzin) | [^33^](#_ENREF_33) |
|  | 6.898 | 50777.4297 | 3.4 | [M+H]^+^ | 417.1142 | 417.19864, 255.11548, 227.08372, 199.0968, 145.06143, 137.1251 |  |  | [^32^](#_ENREF_32) |
| 1.5. Flavanol | | | | | | | | | |
| 43 | 10.37 | 76875.8594 | 6.5 | [M+H]^+^ | 291.0468 | 291.05341, 273.14771, 165.0883, 151.10545, 139.08467 | C_15_H_14_O_6_ | Epicatechin | [^34^](#_ENREF_34) |
| 1.6. Flavanonols | | | | | | | | | |
| 44 | 1.593 | 961342.125 | -3.3 | [M+H]^+^ | 305.0871 | 305.08176, 287.12082, 259.08212, 195.02209, 179.0237 | C_15_H_12_O_7_ | Taxifolin | [^35^](#_ENREF_35) |
| 1.7. Anthocyanins | | | | | | | | | |
| 45 | 1.863 | 5045.70459 | 2.6 | [M]^+^ | 595.3809 | 595.3807, 449.05429, 287.05472, 234.02026, 193.09009 | C_27_H_31_O_15_ | Cyanidin 3-*O*-rutinoside | [^20^](#_ENREF_20) |
| 46 | 3.359 | 203865.656 | 0.9 | [M]^+^ | 625.2092 | 625.07721, 463.15454, 301.11777, 286.07117, 205.03976 | C_28_H_33_O_16_ | Peonidin 3,5-*O*-di-beta-glucopyranoside | [^36^](#_ENREF_36) |
| 47 | 4.966 | 81769.8906 | 1.8 | [M+H]^+^ | 579.1651 | 579.17059, 427.20093, 409.11533, 291.16156, 139.14182 | C_30_H_26_O_12_ | Procyanidin B2 | [^37^](#_ENREF_37) |
| 48 | 5.967 | 22026.0195 | 0.3 | [M]^+^ | 655.1933 | 655.1956, 640.18622, 635.17145, 493.13531, 331.07852, 315.05792, 301.10667, | C_29_H_35_O_17_ | Malvidin 3, 5-di-*O*-glucoside | [^30^](#_ENREF_30) |
| 49 | 6.310 | 807244.688 | 0.8 | [M-H]^-^ | 491.1209 | 445.2070, 354.9279, 329.9082, 286.9385, 218.9490 | C_23_H_25_O_12_ | Malvidin-3-*O*-glucoside | [^38^](#_ENREF_38) |
|  | 7.843 | 377724.188 | 0.3 | [M]^+^ | 493.1345 | 493.1353, 331.0825, 316.05624, 301.03534, 308.1100, 287.06299, 169.1227 |  |  | [^30^](#_ENREF_30) |
| 50 | 7.889 | 69036.9609 | 0.3 | [M]^+^ | 463.1235 | 463.12411, 301.07892, 295.15387, 286.05414 | C_22_H_23_O_11_ | Peonidine 3-*O*-glucoside | [^36^](#_ENREF_36) |
| 51 | 7.302 | 62757792 | -0.2 | [M]^+^ | 611.1684 | 593.1504, 491.1141, 431.0971, 299.0526, 259.1178 | C_27_H_31_O_16_ | Cyanidin 3, 5-di-*O*-glucoside | [^36^](#_ENREF_36) |
| 52 | 7.544 | 46245.3008 | 1.5 | [M]^+^ | 609.3479 | 609.3455, 463.15421, 431.14651, 301. 0731, 286.08011 | C_31_H_29_O_13_ | Peonidin 3-*O*-(6"-*P*-coumaroyl) glucoside | [^20^](#_ENREF_20) |
| 53 | 7.579 | 17078.9844 | 4.6 | [M]^+^ | 639.1951 | 639.1885, 603.30121, 493.10953, 331.08325, 301.03458, 287.06665 | C_32_H_31_O_14_ | Malvinidin 3-*O*-(6"-P-coumaroyl) glucoside | [^20^](#_ENREF_20) |
| 54 | 7.796 | 51245.2695 | 3.7 | [M]^+^ | 450.2431 | 450.24966, 287.0591, 271.10236,127.02818, 119.04661 | C_21_H_21_O_11_ | Cyanidin 3-*O*-glucoside | [^20^](#_ENREF_20) |
| 55 | 8.000 | 28889.373 | 6.3 | [M]^+^ | 579.1793 | 579.1793, 433.30368, 271.21234, 251.18253, 113.07073 | C_27_H_31_O_14_ | Pelargonidin 3-*O*-rutinoside | [^20^](#_ENREF_20) |
| 2- Acids | | | | | | | | | |
| 2.1. Phenolic acids | | | | | | | | | |
| 56 | 1.12 | 607816.68 | -3.1 | [M-H]^-^ | 191.0572 | 191.0571,173.0459, 127.0412, 85.0292 | C_7_H_12_O_6_ | Quinic acid | [^21^](#_ENREF_21) |
| 57 | 1.27 | 360880.12 | 0.5 | [M-H]^-^ | 315.072  0 | 153.0195, 135.0299, 109.0300 | C_13_H_16_O_9_ | Protocatechuic acid 4-O-hexoside | [^39^](#_ENREF_39) |
| 58 | 1.361 | 312822.90 | 5.1 | [M-H]^-^ | 167.0337 | 152.0114, 108.0231 | C_8_H_8_O_4_ | 5-Methoxysalicylic acid | [^40^](#_ENREF_40) |
| 59 | 1.71 | 15059863 | -0.8 | [M-H]^-^ | 163.0404 | 119. 0504, 93.0347 | C_9_H_8_O_3_ | *P*-Coumaric acid | [^15^](#_ENREF_15) |
|  | 5.13 | 118754 | -5.7 | [M+H]^+^ | 165.0919 | 165.09204, 147.0787, 121.06702, 119.08661, 103.04188, 91.04698, 77.04075 |  |  | [^41^](#_ENREF_41) |
| 60 | 1.84 | 659334.438 | 0.7 | [M-H]^-^ | 193.0501 | 178.0270, 134.0364, 133.02628, 106.0423 | C_10_H_10_O_4_ | Ferulic Acid | [^42^](#_ENREF_42) |
| 61 | 2.03 | 7477941 | -1.6 | [M-H]^-^ | 179.0573 | 161.8714, 150.0543, 135.0446 | C_9_H_8_O_4_ | Caffeic acid | [^15^](#_ENREF_15) |
|  | 3.52 | 85659.6875 | 8.5 | [M+H]^+^ | 181.0882 | 163.0840, 135.0916 |  |  | [^5^](#_ENREF_5) |
| 62 | 2.66 | 100197.54 | -2.5 | [M-H]^-^ | 137.0252 | 93.0350, 65.0419 | C_7_H_6_O_3_ | *P*- Hydroxybenzoic acid | [^10^](#_ENREF_10) |
|  | 10.40 | 252269.969 | -4 | [M+H]^+^ | 139.1123 | 139.11284, 121.09556, 95.08503, 77.0435, 67.05243 |  |  | [^41^](#_ENREF_41) |
| 63 | 2.68 | 67803 | 0.9 | [M-H]^-^ | 353.0876 | 353.0804, 191.0537, 179.0348, 135.0459, 111.0478 | C_16_H_18_O_9_ | Chlorogenic acid | [^15^](#_ENREF_15) |
|  | 4.60 | 45276.3906 | −2.9 | [M+H]^+^ | 355.1035 | 193.0499, 163.0373, 145.0282, 117.0306 |  |  | [^5^](#_ENREF_5) |
| 64 | 2.84 | 485992.21 | 0.2 | [M-H]^-^ | 146.9611 | 102.9721, 100.9587 | C_9_H_8_O_2_ | Cinnamic acid | [^7^](#_ENREF_7) |
| 65 | 3.55 | 264150.406 | -0.5 | [M-H]^-^ | 167.0369 | 123.0439, 91.0205 | C_8_H_8_O_4_ | Vanillic acid | [^5^](#_ENREF_5) |
| 66 | 3.91 | 374750.90 | 3.2 | [M-H]- | 359.052 | 197.0431, 161.0234 | C_18_H_16_O_8_ | Rosmarinic acid | [^10^](#_ENREF_10) |
| 67 | 3.92 | 223643.14 | 6.3 | [M-H]^-^ | 341.0879 | 179.0356, 135.0461 | C_15_H_18_O_9_ | Caffeic acid-*O*-hexoside | [^43^](#_ENREF_43) |
| 68 | 5.82 | 11545.9854 | -4.9 | [M+H]^+^ | 138.0568 | 138.05206, 121.07365, 66.02695 | C_7_H_7_NO | *P* - amino benzoic acid | [^41^](#_ENREF_41) |
| 69 | 10.08 | 486977.78 | 5.2 | [M-H]- | 177.0203 | 133.0292, 117.0346 | C_10_H_10_O_3_ | 4-Methoxycinnamic acid | [^44^](#_ENREF_44) |
| 70 | 27.50 | 177230.93 | -1.5 | [M-H]^-^ | 169.0135 | 125.0233, 107.0145, 97.0313,79.0206 | C_7_H_6_O_5_ | Gallic acid | [^43^](#_ENREF_43) |
|  | 14.95 | 66940.2188 | 9.3 | [M+H]^+^ | 171.1535 | 156.0885, 141.0693,127.0352, 125.9856, 109.0275 |  |  | [^5^](#_ENREF_5) |
| 2.2. Carboxylic acid | | | | | | | | | |
| 71 | 1.04 | 957324.813 | 0.5 | [M-H]^-^ | 117.0189 | 99.0100, 73.0301 | C_4_H_6_O_4_ | Succinic acid | [^45^](#_ENREF_45) |
| 72 | 1.101 | 756708.93 | 0.6 | [M-H]^-^ | 133.0143 | 115.0038, 72.9935, 71.0139 | C_4_H_6_O_5_ | Malic acid | [^46^](#_ENREF_46) |
| 73 | 1.103 | 514920.65 | 0 | [M-H]^-^ | 173.0088 | 129.0198, 85.0301 | C_6_H_6_O_6_ | Aconitic acid | [^10^](#_ENREF_10) |
| 74 | 1.13 | 269514.75 | 8.5 | [M-H]^-^ | 149.0433 | 103.0397, 87.0367, 73.0296, 59.0137 | C_4_H_6_O_6_ | Tartaric acid | [^47^](#_ENREF_47) |
| 75 | 1.350 | 167045.625 | 0.8 | [M-H]^-^ | 155.0336 | 127.0439, 111.0519 | C_5_H_4_N_2_O_4_ | Orotic acid | [^48^](#_ENREF_48) |
| 76 | 3.72 | 255399.734 | 0.8 | [M-H]^-^ | 151.04 | 121.0288, 107.0511, 105.0345 | C_8_H_8_O_3_ | Mandelic acid | [^49^](#_ENREF_49) |
| 77 | 4.38 | 81720.11 | 0 | [M-H]^-^ | 173.0817 | 154.9481, 136.9358 | C_7_H_10_O_5_ | Shikimic acid | [^50^](#_ENREF_50) |
| 78 | 26.92 | 247047.59 | -7.9 | [M-H]^-^ | 114.9967 | 99.9266,70.9990 | C_4_H_4_O_4_ | Maleic acid | [^29^](#_ENREF_29) |
| 79 | 27.09 | 88074.2813 | 3.2 | [M-H]^-^ | 130.9673 | 113.9587, 86.9952, 69.0231 | C_5_H_8_O_4_ | Glutaric acid | [^51^](#_ENREF_51) |
| 80 | 27.52 | 66039.7109 | -9.4 | [M-H]^-^ | 102.9560 | 88.9747, 59.0012 | C_3_H_4_O_4_ | Malonic acid | [^51^](#_ENREF_51) |
| 3. Coumarins | | | | | | | | | |
| 81 | 3.603 | 1167204.13 | -1.9 | [M-H]^-^ | 339.0723 | 178.0274, 177.0208, 133.0307 | C_15_H_16_O_9_ | Esculin | [^37^](#_ENREF_37) |
| 82 | 4.545 | 57409.7461 | -1.7 | [M+H]^+^ | 193.0500 | 193.05066, 178.0266, 165.11424, 150.93846, 132.0918 | C_10_H_8_O_4_ | Scopoletin | [^52^](#_ENREF_52) |
| 83 | 5.310 | 673800.563 | -0.5 | [M+H]^+^ | 179.0339 | 179.03079, 151.04105, 135.02811, 117.03687, 99.03849 | C_9_H_6_O_4_ | Esculetin | [^53^](#_ENREF_53) |
| 84 | 7.90 | 3358247.5 | 5.1 | [M-H]^-^ | 177.0547 | 149.0242, 133.0297, 121.0298, 105.0348 | C_9_H_6_O_4_ | Daphnetin | [^54^](#_ENREF_54) |
| 85 | 13.258 | 453199.469 | 2 | [M+H]^+^ | 177.1619 | 177.15709, 149.02031, 133.11095, 115.06686 | C_10_H_8_O_3_ | 7- Hydroxy-4-methyl coumarin | [^52^](#_ENREF_52) |
| 4. Stilbenes | | | | | | | | | |
| 86 | 1.790 | 521593.625 | 8.2 | [M+H]^+^ | 407.1125 | 407.11295, 245.06194, 227.12402, 209.06758, 191.05307 | C_20_H_22_O_9_ | E-3,4,5'-Trihydroxy-3'-glucopyranosylstilbene  (Astringin) | [^55^](#_ENREF_55) |
| 87 | 10.37 | 68351.4609 | 0.4 | [M+H]^+^ | 229.0811 | 211.1388, 170.0918, 135.0720, 107.0867 | C_14_H_12_O_3_ | Resveratrol | [^55^](#_ENREF_55) |
| 5. Others | | | | | | | | | |
| 88 | 1.312 | 32917.3906 | 1.7 | [M-H]^-^ | 315.1099 | 153.01839, 123.0440 | C_14_H_20_O_8_ | Dimethoxyphenylβ-D glucopyranoside | [^5^](#_ENREF_5) |
| 89 | 2.61 | 695260.25 | -2.5 | [M-H]^-^ | 137.0252 | 93.0329, 65.0419 | C_7_H_6_O_3_ | Protocatechuic aldehyde | [^10^](#_ENREF_10) |
| 90 | 5.335 | 686222.063 | 0.2 | [M+H]^+^ | 215.1252 | 215.12195, 197.07848, 173.0909, 169.0826 | C_13_H_14_N_2_O | Harmaline | [^56^](#_ENREF_56) |
| 91 | 5.37 | 377321.62 | -1 | [M-H]^-^ | 385.188 | 266.0517, 247.0642, 223.1367, 205.1276, 153.0921 | C_17_H_22_O_10_ | 1-*O*-*β*-D-glucopyranosyl sinapate | [^57^](#_ENREF_57) |
|  | 3.51 | 419336.75 | 2.1 | [M+H]^+^ | 387.1610 | 387.16653, 225.87259, 210.44547, 207.99802, 195.05479 |  |  | [^41^](#_ENREF_41) |
| 92 | 5.824 | 96805.804 | 0.1 | [M+H]^+^ | 138.0552 | 111.0083 | C_7_H_7_NO_2_ | Trigonelline | [^58^](#_ENREF_58) |
| 93 | 10.53 | 1167528.25 | 5.2 | [M-H]^-^ | 207.0652 | 192.0431, 163.0860, 148.0509, 134.0352, 118.9617 | C_11_H_12_O_4_ | Sinapyl aldehyde | [^59^](#_ENREF_59) |
| 94 | 13.23 | 163989.89 | -10 | [M]^+^ | 334.2031 | 316.2051, 298.1872, 166.0872, 100.1114 | C_20_H_30_O_4_ | Cymbodiacetal | [**^60^**](#_ENREF_60) |
| 95 | 17.22 | 1344679.25 | 0.7 | [M-H]^-^ | 151.0401 | 108.0209, 80.0368 | C_5_H_4_N_4_O_2_ | Xanthine | [^61^](#_ENREF_61) |

*** References used for conformation of the obtained mass data of each compound**

1 Hussein, S. R. *et al.* Spectrometric analysis, phenolics isolation and cytotoxic activity of Stipagrostis plumosa (Family Poaceae). *Chemical Papers* **72**, 29-37 (2018).

2 Abd Ghafar, S. Z., Mediani, A., Ramli, N. S. & Abas, F. Antioxidant, α-glucosidase, and nitric oxide inhibitory activities of Phyllanthus acidus and LC–MS/MS profile of the active extract. *Food Bioscience* **25**, 134-140 (2018).

3 Fang, T. *et al.* A rapid LC/MS/MS quantitation assay for naringin and its two metabolites in rats plasma. *Journal of pharmaceutical and biomedical analysis* **40**, 454-459 (2006).

4 Zhao, W. *et al.* Rapid screening and identification of daidzein metabolites in rats based on UHPLC-LTQ-orbitrap mass spectrometry coupled with data-mining technologies. *Molecules* **23**, 151 (2018).

5 Liu, M.-H. *et al.* Chemical profiles, antioxidant and anti-obesity effects of extract of Bambusa textilis McClure leaves. *Journal of Functional Foods* **22**, 533-546 (2016).

6 Hoyweghen, L. V., Beer, T. D., Deforce, D. & Heyerick, A. Phenolic compounds and anti‐oxidant capacity of twelve morphologically heterogeneous bamboo species. *Phytochemical Analysis* **23**, 433-443 (2012).

7 Zhang, W., Xu, M., Yu, C., Zhang, G. & Tang, X. Simultaneous determination of vitexin-4 ″-O-glucoside, vitexin-2 ″-O-rhamnoside, rutin and vitexin from hawthorn leaves flavonoids in rat plasma by UPLC–ESI-MS/MS. *Journal of Chromatography B* **878**, 1837-1844 (2010).

8 Ying, X., Lu, X., Sun, X., Li, X. & Li, F. Determination of vitexin-2 ″-O-rhamnoside in rat plasma by ultra-performance liquid chromatography electrospray ionization tandem mass spectrometry and its application to pharmacokinetic study. *Talanta* **72**, 1500-1506 (2007).

9 He, Z.-H., Liu, M., Ren, J.-X. & Ouyang, D.-W. Structural characterization of chemical compounds based on their fragmentation rules in Sophorae Fructus by UPLC-QTOF-MS/MS. *Pharmaceutical Fronts* **4**, e162-e178 (2022).

10 Gedük, A. Ş. & Atsız, S. LC-MS/MS phenolic composition of peach (Prunus persica (L.) Batsch) extracts and an evaluation of their antidiabetic, antioxidant, and antibacterial activities. *South African Journal of Botany* **147**, 636-645 (2022).

11 van Der Klift, E., Villela, A., Derksen, G. C., Lankhorst, P. P. & van Beek, T. A. Microextraction of reseda luteola-dyed wool and qualitative analysis of its flavones by uhplc-uv, nmr and ms. *Molecules* **26**, 3787 (2021).

12 Tsimogiannis, D., Samiotaki, M., Panayotou, G. & Oreopoulou, V. Characterization of flavonoid subgroups and hydroxy substitution by HPLC-MS/MS. *Molecules* **12**, 593-606 (2007).

13 Figueirinha, A., Paranhos, A., Pérez-Alonso, J. J., Santos-Buelga, C. & Batista, M. T. Cymbopogon citratus leaves: Characterization of flavonoids by HPLC–PDA–ESI/MS/MS and an approach to their potential as a source of bioactive polyphenols. *Food chemistry* **110**, 718-728 (2008).

14 Mekky, R. H., Abdel-Sattar, E., Segura-Carretero, A. & Contreras, M. d. M. Metabolic profiling of the oil of Sesame of the Egyptian cultivar ‘Giza 32’employing LC-MS and tandem ms-based untargeted method. *Foods* **10**, 298 (2021).

15 Madi, Y. F., Choucry, M. A., El-Marasy, S. A., Meselhy, M. R. & El-Kashoury, E.-S. A. UPLC-Orbitrap HRMS metabolic profiling of Cymbopogon citratus cultivated in Egypt; neuroprotective effect against AlCl3-induced neurotoxicity in rats. *Journal of Ethnopharmacology* **259**, 112930 (2020).

16 Jaiswal, R., Müller, H., Müller, A., Karar, M. G. E. & Kuhnert, N. Identification and characterization of chlorogenic acids, chlorogenic acid glycosides and flavonoids from Lonicera henryi L.(Caprifoliaceae) leaves by LC–MSn. *Phytochemistry* **108**, 252-263 (2014).

17 Wang, P. *et al.* Chemical and genetic discrimination of commercial Guangchenpi (Citrus reticulata ‘Chachi’) by using UPLC-QTOF-MS/MS based metabolomics and DNA barcoding approaches. *RSC advances* **9**, 23373-23381 (2019).

18 Zhi, H. *et al.* Exploring the effective materials of flavonoids-enriched extract from Scutellaria baicalensis roots based on the metabolic activation in influenza A virus induced acute lung injury. *Journal of pharmaceutical and biomedical analysis* **177**, 112876 (2020).

19 Huck, C., Buchmeiser, M. & Bonn, G. Fast analysis of flavonoids in plant extracts by liquid chromatography–ultraviolet absorbance detection on poly (carboxylic acid)-coated silica and electrospray ionization tandem mass spectrometric detection. *Journal of Chromatography A* **943**, 33-38 (2002).

20 Bationo, R. K. *et al.* Major anthocyanin quantification, free radical scavenging properties and structural identification in Cymbopogon giganteus extracts. *African Journal of Pure and Applied Chemistry* **17**, 32-46 (2023).

21 M. Elkady, W., M. Ayoub, I., Abdel-Mottaleb, Y., ElShafie, M. F. & Wink, M. Euryops pectinatus L. flower extract inhibits P-glycoprotein and reverses multi-drug resistance in cancer cells: A mechanistic study. *Molecules* **25**, 647 (2020).

22 De Rosso, M. *et al.* Identification of new flavonols in hybrid grapes by combined liquid chromatography–mass spectrometry approaches. *Food chemistry* **163**, 244-251 (2014).

23 Gu, D., Yang, Y., Abdulla, R. & Aisa, H. A. Characterization and identification of chemical compositions in the extract of Artemisia rupestris L. by liquid chromatography coupled to quadrupole time‐of‐flight tandem mass spectrometry. *Rapid Communications in Mass Spectrometry* **26**, 83-100 (2012).

24 Jiang, Z. *et al.* Simultaneous determination of kaempferide, kaempferol and isorhamnetin in rat plasma by ultra-high performance liquid chromatography-tandem mass spectrometry and its application to a pharmacokinetic study. *Journal of the Brazilian chemical society* **29**, 535-542 (2018).

25 Abdel-Hamed, A. R. *et al.* Plicosepalus acacia extract and its major constituents, methyl gallate and quercetin, potentiate therapeutic angiogenesis in diabetic hind limb ischemia: HPTLC quantification and LC-MS/MS metabolic profiling. *Antioxidants* **10**, 1701 (2021).

26 Guo, P. *et al.* Simultaneous determination of linarin, naringenin and formononetin in rat plasma by LC‐MS/MS and its application to a pharmacokinetic study after oral administration of Bushen Guchi Pill. *Biomedical Chromatography* **29**, 246-253 (2015).

27 Zeng, X. *et al.* Uflc-q-tof-ms/ms-based screening and identification of flavonoids and derived metabolites in human urine after oral administration of exocarpium citri grandis extract. *Molecules* **23**, 895 (2018).

28 Peng, Y., Chen, S. H., Liu, X. N. & Sun, Q. Y. Efficacy of different antidiabetic drugs based on metformin in the treatment of type 2 diabetes mellitus: a network meta‐analysis involving eight eligible randomized‐controlled trials. *Journal of Cellular Physiology* **234**, 2795-2806 (2019).

29 Alqahtani, M. J., Elekhnawy, E., Negm, W. A., Mahgoub, S. & Hussein, I. A. Encephalartos villosus Lem. Displays a strong in vivo and in vitro antifungal potential against Candida glabrata clinical isolates. *Journal of Fungi* **8**, 521 (2022).

30 Buzgaia, N., Lee, S. Y., Rukayadi, Y., Abas, F. & Shaari, K. Antioxidant activity, α-glucosidase inhibition and UHPLC–ESI–MS/MS profile of shmar (Arbutus pavarii Pamp). *Plants* **10**, 1659 (2021).

31 Ćirić, A., Prosen, H., Jelikić-Stankov, M. & Đurđević, P. Evaluation of matrix effect in determination of some bioflavonoids in food samples by LC–MS/MS method. *Talanta* **99**, 780-790 (2012).

32 Nakata, R. *et al.* A fragmentation study of isoflavones by IT-TOF-MS using biosynthesized isotopes. *Bioscience, Biotechnology, and Biochemistry* **82**, 1309-1315 (2018).

33 Prasain, J. K. *et al.* Identification of isoflavone glycosides in Pueraria lobata cultures by tandem mass spectrometry. *Phytochemical Analysis: An International Journal of Plant Chemical and Biochemical Techniques* **18**, 50-59 (2007).

34 Chang, C.-L. & Wu, R.-T. Quantification of (+)-catechin and (−)-epicatechin in coconut water by LC–MS. *Food Chemistry* **126**, 710-717 (2011).

35 Yang, P. *et al.* Detection of 191 taxifolin metabolites and their distribution in rats using HPLC-ESI-IT-TOF-MSn. *Molecules* **21**, 1209 (2016).

36 Singh, H. P., Singh, T. G. & Singh, R. Evaluation of the renoprotective effect of syringic acid against nephrotoxicity induced by cisplatin in rats. *Journal of Applied Pharmaceutical Science* **11**, 080â€“085 (2021).

37 Li, K. *et al.* Rapid identification of anthocyanin from the epicarp of kadsura coccinea (lem.) AC Smith by UHPLC-Q-exactive orbitrap mass spectrometry. *Food Analytical Methods* **14**, 2545-2555 (2021).

38 Sun, J., Lin, L. z. & Chen, P. Study of the mass spectrometric behaviors of anthocyanins in negative ionization mode and its applications for characterization of anthocyanins and non‐anthocyanin polyphenols. *Rapid Communications in Mass Spectrometry* **26**, 1123-1133 (2012).

39 Grati, W. *et al.* HESI-MS/MS analysis of phenolic compounds from Calendula aegyptiaca fruits extracts and evaluation of their antioxidant activities. *Molecules* **27**, 2314 (2022).

40 Sruthi, D. & Zachariah, T. Phenolic profiling of Piper species by liquid chromatography-mass spectrometry. (2016).

41 El-sayed, M., Abbas, F. A., Refaat, S., El-Shafae, A. M. & Fikry, E. UPLC-ESI-MS/MS profile of the ethyl acetate fraction of aerial parts of Bougainvillea'Scarlett O'Hara'cultivated in Egypt. *Egyptian Journal of Chemistry* **64**, 793-806 (2021).

42 Fang, N., Yu, S. & Badger, T. M. Characterization of triterpene alcohol and sterol ferulates in rice bran using LC-MS/MS. *Journal of Agricultural and Food Chemistry* **51**, 3260-3267 (2003).

43 Abdurehman, D. *et al.* Optimization of preparation method of hepatoprotective active components from Coreopsis tinctoria Nutt. and its action mechanism in vivo. *Biomedicine & Pharmacotherapy* **167**, 115590 (2023).

44 Tang, K. S., Konczak, I. & Zhao, J. Phenolic compounds of the Australian native herb Prostanthera rotundifolia and their biological activities. *Food Chemistry* **233**, 530-539 (2017).

45 Fiori, J. *et al.* Cellular and mitochondrial determination of low molecular mass organic acids by LC–MS/MS. *Journal of Pharmaceutical and Biomedical Analysis* **150**, 33-38 (2018).

46 Alotaibi, B., Tousson, E., El‐Masry, T. A., Altwaijry, N. & Saleh, A. Ehrlich ascites carcinoma as model for studying the cardiac protective effects of curcumin nanoparticles against cardiac damage in female mice. *Environmental toxicology* **36**, 105-113 (2021).

47 Higginson, E., Lloyd, N., Kravchuk, O., Ford, C. & Thomas, M. R. A high‐throughput UHPLC MS/MS method for evaluation of tartaric and malic acid concentration in individual grapevine berries. *Australian Journal of Grape and Wine Research* **22**, 16-23 (2016).

48 Marca, G. l., Casetta, B. & Zammarchi, E. Rapid determination of orotic acid in urine by a fast liquid chromatography/tandem mass spectrometric method. *Rapid communications in mass spectrometry* **17**, 788-793 (2003).

49 Zahoor, M., Shafiq, S., Ullah, H., Sadiq, A. & Ullah, F. Isolation of quercetin and mandelic acid from Aesculus indica fruit and their biological activities. *BMC biochemistry* **19**, 1-14 (2018).

50 Noh, K., Back, H.-M., Shin, B. S. & Kang, W. Pharmacokinetics of shikimic acid following intragastric and intravenous administrations in rats. *Pharmaceutics* **12**, 824 (2020).

51 Bylund, D., Norström, S. H., Essén, S. A. & Lundström, U. S. Analysis of low molecular mass organic acids in natural waters by ion exclusion chromatography tandem mass spectrometry. *Journal of Chromatography A* **1176**, 89-93 (2007).

52 Wang, K. *et al.* Identification of components in Citri Sarcodactylis Fructus from different origins via UPLC-Q-Exactive Orbitrap/MS. *ACS omega* **6**, 17045-17057 (2021).

53 Kim, J.-S., Ha, T.-Y., Ahn, J. & Kim, S. Analysis and distribution of esculetin in plasma and tissues of rats after oral administration. *Preventive nutrition and food science* **19**, 321 (2014).

54 Su, X. L., Lin, R. C., Wong, S. K., Tsui, S. K. & Kwan, S. Y. Identification and characterisation of the Chinese herb Langdu by LC‐MS/MS analysis. *Phytochemical Analysis: An International Journal of Plant Chemical and Biochemical Techniques* **14**, 40-47 (2003).

55 Wang, H. *et al.* Comprehensive analysis of pterostilbene metabolites in vivo and in vitro using a UHPLC-Q-exactive plus mass spectrometer with multiple data-mining methods. *ACS omega* **7**, 38561-38575 (2022).

56 Thompson, A. F. *Forensic analysis of the psychoactive alkaloids harmine and harmaline in peganum harmala seeds*, Boston University, (2013).

57 Son, S.-W. *et al.* Anti-melanoma activity of Cynanchi atrati Radix is mediated by regulation of NF-kappa B activity and pro-apoptotic proteins. *Journal of Ethnopharmacology* **153**, 250-257 (2014).

58 Lang, R., Wahl, A., Stark, T. & Hofmann, T. Urinary N‐methylpyridinium and trigonelline as candidate dietary biomarkers of coffee consumption. *Molecular nutrition & food research* **55**, 1613-1623 (2011).

59 Li, L., Popko, J. L., Umezawa, T. & Chiang, V. L. 5-Hydroxyconiferyl aldehyde modulates enzymatic methylation for syringyl monolignol formation, a new view of monolignol biosynthesis in angiosperms. *Journal of Biological Chemistry* **275**, 6537-6545 (2000).

60 Bottini, A. T. *et al.* Isolation and crystal structure of a novel dihemiacetal bis-monoterpenoid from Cymbopogon martinii. *Phytochemistry* **26**, 2301-2302 (1987).

61 Rukdee, N., Rojsanga, P. & Phechkrajang, C. M. Development and validation of LC-MS/MS method for quantitative determination of adenosine, guanosine, xanthine and uric acid in widely consumed vegetables in Thailand. *Natural Product Communications* **10**, 1934578X1501000831 (2015).

**Supplementary 5: Ethical approval certificate**


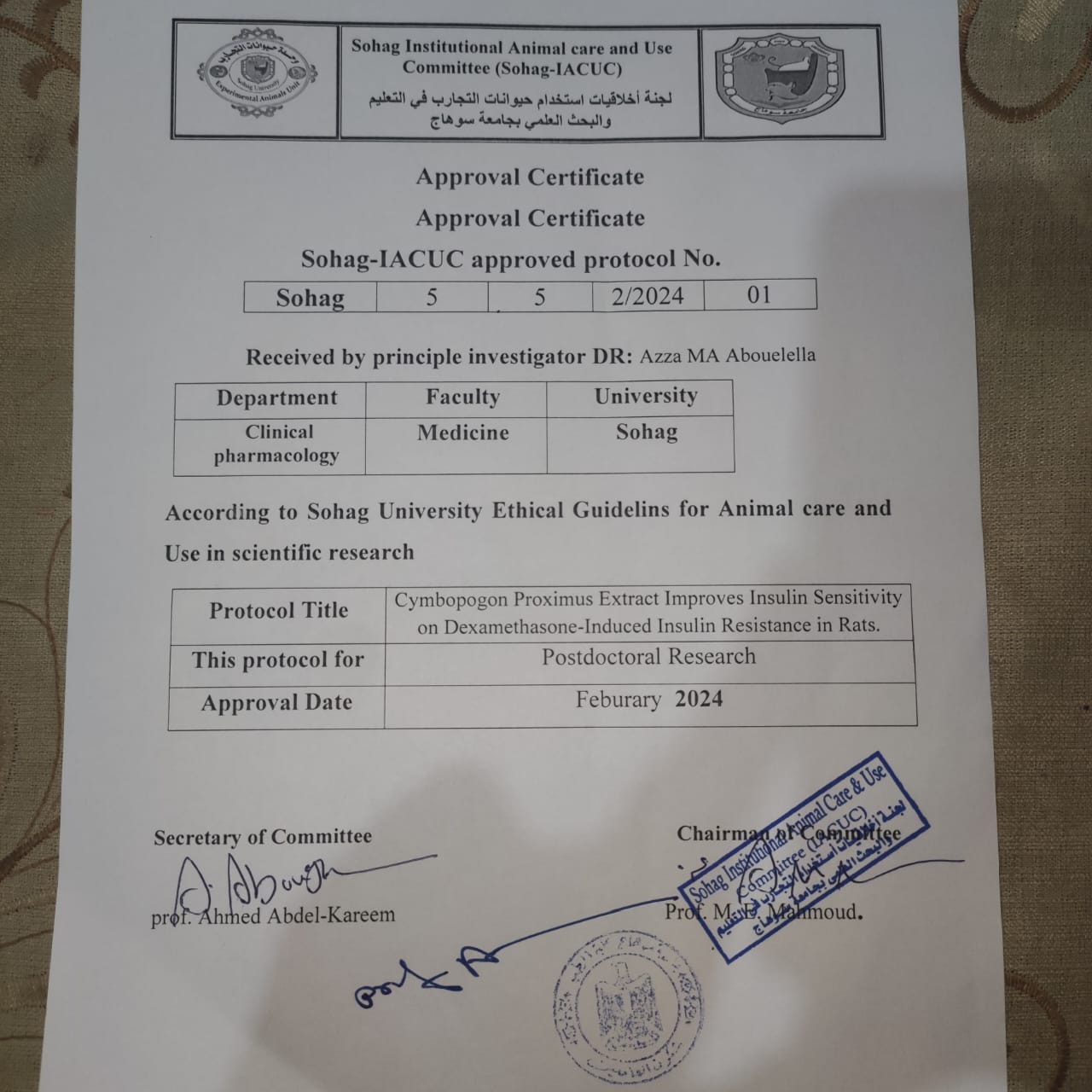


**Fig S: ethical approval certificate**

**Supplementary 6: ARRIVE Essential form**

**Fig. Y: ARRIVE Essential form**
